# Constraints on mountain building in the northeastern Tibet: Detrital zircon records from synorogenic deposits in the Yumen Basin

**DOI:** 10.1038/srep27604

**Published:** 2016-06-09

**Authors:** Weitao Wang, Peizhen Zhang, Jingxing Yu, Yizhou Wang, Dewen Zheng, Wenjun Zheng, Huiping Zhang, Jianzhang Pang

**Affiliations:** 1 State Key Laboratory of Earthquake Dynamics, Institute of Geology, China Earthquake Administration, Beijing, China; 2 School of Earth Science and Geological Engineering, Sun Yan-Sen University,Guangzhou, China

## Abstract

The Cenozoic basins and ranges form the high topography of the northeastern Tibet that resulted from the India-Eurasia collision. Sedimentary rocks in the basins provide direct insight into the exhumation history of the ranges and the tectonic processes that led to the northeastward growth of the Tibetan Plateau. In this study, we analyzed and compared detrital zircon U-Pb ages from sands of modern rivers draining the Bei Shan, and North Qilian Shan and sandstones from the Yumen Basin. The zircon age distributions indicate that the strata dated to 24.2-16.7 Ma in the basin were derived from the Bei Shan, and the basin provenance changed rapidly to the North Qilian Shan terrane at ~16 Ma. These results suggest that an early stage of deformation along the Bei Shan at ~24 Ma was replaced by the growth of the North Qilian Shan at ~16 Ma. We conclude that the far-field effect associated with the Indo-Asian collision may result from Oligocene deformation in the Bei Shan, but the emergence of the North Qilian Shan at ~16 Ma could reflect the most recent outward growth of the Tibetan Plateau that may have been caused by the removal of some lithospheric mantle beneath central Tibet.

The upward growth histories of the margins of the Tibetan Plateau to its present elevation remain at the center of the debate about the dynamics of continental deformation^1^,^2^,^3^ and climate change in the Cenozoic^4^,^5^. In the northeastern Tibetan Plateau, recent studies have called attention to two hypotheses that illuminate different frameworks of lithospheric rheology and deformation mechanisms for the growth of the Tibetan Plateau^6^. First, a constant bulk strain model^7^ suggests a constant average stress has been applied to the plateau from the south to the north since the India -Asian collision in the early Cenozoic. This model infers simultaneous deformation occurred across a vast region of the Tibetan Plateau, such as the northeastern part of the plateau. Second, an oblique stepwise continental subduction model^3^ predicts that the plateau grew progressively from south to north, and the northeastern part of the plateau has risen most recently (Pliocene-Pleistocene). The Qilian Shan and the Bei Shan, located in the outermost parts of northeastern Tibetan Plateau (Fig. 1A), play a central role in testing these ideas.

Despite numerous recent studies^8^,^9^, the timing of active and Cenozoic deformation in the Qilian Shan-Bei Shan region remains ambiguous, with ages ranging from the Oligocene to the early Pleistocene. This situation may arise because of differences in the proxy data that have been used to infer the timing of mountain building, such as subsidence history from the sedimentary records around the ranges^9^,^10^, and low-temperature thermochronology from the hanging wall blocks of thrust systems in the Qilian Shan^11^ where cooling is inferred to reflect exhumation of the mountain range. In the basin, sediments eroded from the nearby mountains and transported by the rivers can record precise information on the unroofing history of the range, based on provenance analysis. Single-grain U-Pb dating of the detrital zircons preserved in sediments is a powerful method to trace provenance because it can fingerprint source areas with distinctive zircon age populations^12^,^13^,^14^. Comparing the distributions of zircon U-Pb ages from the sedimentary succession to U-Pb ages of modern river sands from the Qilian Shan and the Bei Shan can illuminate the processes of deformation in the northeastern Tibet if the depositional age of the sediments in the basin is well defined.

Here, we present ~1000 single-grain U-Pb zircon ages from the modern streams draining the North Qilian Shan, the Bei Shan and from late Oligocene to late Pliocene sediment sequence in the Yumen Basin that were dated using magnetostratigraphy. These data clearly record late Oligocene deformation in the Bei Shan and the growth of the North Qilian Shan at ~16 Ma, represented by a distinct change in the detrital-age signature as rocks began to erode from the North Qilian Shan.

## Geological Setting and Sampling

**Figure 1:**
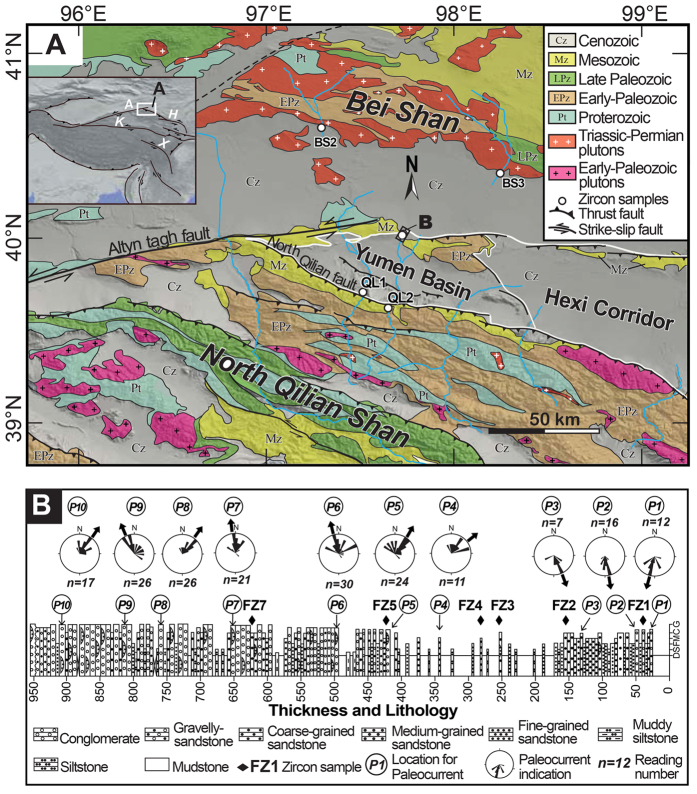
(**A**) Geologic map of the northeastern Tibetan Plateau and the locations of the detrital zircon samples from the Bei Shan, the North Qilian Shan and the Yumen Basin. Inset shows the location of (**A**), which was created by ArcGIS 9.2 (http://www.esri.com/software/arcgis/arcgis-for-desktop). In the inset map, A- Altyn Tagh Fault, H-Haiyuan Fault, K-Kunlun Fault, X-Xianshuihe Fault, HL-Honghe Fault, Q-Qaidam Basin. (**B**) Stratigraphic column of the Caogou section with the detailed locations of the detrital zircon samples and paleocurrent measurements within the section. (**B**) was created by CorelDRAW X7 (http://www.coreldraw.com/us/pages/free-download).

Along the northeastern Tibetan Plateau, the Qilian Shan is truncated by the left-lateral strike-slip Altyn Tagh Fault (ATF) to the west. Movement along the ATF transfers a significant amount of the convergence between India and Asia into eastward extrusion of the northern plateau^3^,^15^ and crustal thickening and shortening in the region^16^. In combination with the ATF propagating northeastward, the Qilian Shan grows outward toward the foreland, creating a series of NW dextral transpressional faults and associated crustal-scale ramp anticlines that divide the Hexi Corridor Basin into several sub-basins. Our study sites are located in the Yumen Basin, which is the westernmost sub-basin of the Hexi Corridor Basin, in the transition area between the topographic front of the Qilian Shan and in the Bei Shan (Fig. 1A).

The Cenozoic strata of the Yumen Basin are well exposed and consist of five primary stratigraphic units: the Oligocene Huoshaogou Formation, the late Oligocene to early Miocene Baiyanghe Formation, the middle Miocene to Pliocene Shulehe Formation, the Late Pliocene Yumen conglomerate, and the Quaternary Jiuquan conglomerate. The age of these stratigraphic units is based on fossils of mammalian, ostracode, and palynological assemblages and magnetostratigraphy^8^,^17^.

We selected one of the most complete and continuous stratigraphic sections along the northern margin of the basin, from which to collect detrital zircon samples (Fig. 1B). This section, known as the Caogou section, was dated using high-resolution magnetostratigraphic dating and was deposited between ~24.2 Ma and ~2.8 Ma^17^. Six fine- to coarse-grained sandstone samples were collected from the section and from sediments dated to 23.8 Ma, 20.6 Ma, 16.7 Ma, 15.8 Ma, 13.2 Ma and 7.5 Ma (Supplementary Fig. S1). In addition, we collected four sand samples from modern rivers (Fig. 1A) to establish distinctive U-Pb age signatures of the North Qilian Shan and the Bei Shan. Although the density of modern sand samples is not large enough to account for all of the streams draining the North Qilian Shan and the Bei Shan, the sampled rivers cross most lithologic units of both ranges that were expected to yield zircons.

## Methodology

Five kilograms of sand or sandstone was collected for each sample from a single outcrop for detrital zircon analysis. Zircon crystals in these samples were isolated using a combined method with heavy liquids and a magnetic separator; the analyzed grains were hand picked at random and mounted in epoxy resin. Measurements for the U-Pb analysis of individual detrital zircon grains were carried out by LA-ICP-MS spectrometry, and the equipment is housed at the Institute of Geology and Geophysics, Chinese Academy of Sciences. Details of the analytical procedures were previously described by Xie *et al*.^18^.

The results and fractionation correction of the detrital zircons were calculated using GLITTER 4.0 software. We interpreted the U-Pb ages based on ^206^Pb/^238^U for grains younger than 1000 Ma, and ^207^Pb/^206^Pb for grains older than 1000 Ma. In general, ^206^Pb/^238^U ages are more precise for younger ages, whereas ^206^Pb/^207^Pb ages are more precise for older ages. All of the analytical results are listed in the Supplemental Table 1. Although all zircon U-Pb ages are presented, only the zircon ages with ≤25% discordance or ≤10% reverse discordance were used to build the age-probability diagrams. In this paper, the detrital zircon age populations for individual samples are plotted on relative age-probability diagrams derived from the probability density function^19^.

Paleocurrent indicators were also measured from the Caogou section and from other outcrops where such features were accessible. Post-depositional tilting and vertical axes rotation of these paleocurrent indicators were corrected for tilted sediment bedding and rotations as indicated by the paleomagnetic data^17^. The combination of zircon grain ages with general paleocurrent orientations in the Yumen Basin allows for identification of the predominant source of the basin sediments and the unroofing history of the adjacent ranges.

## Results

**Figure 2:**
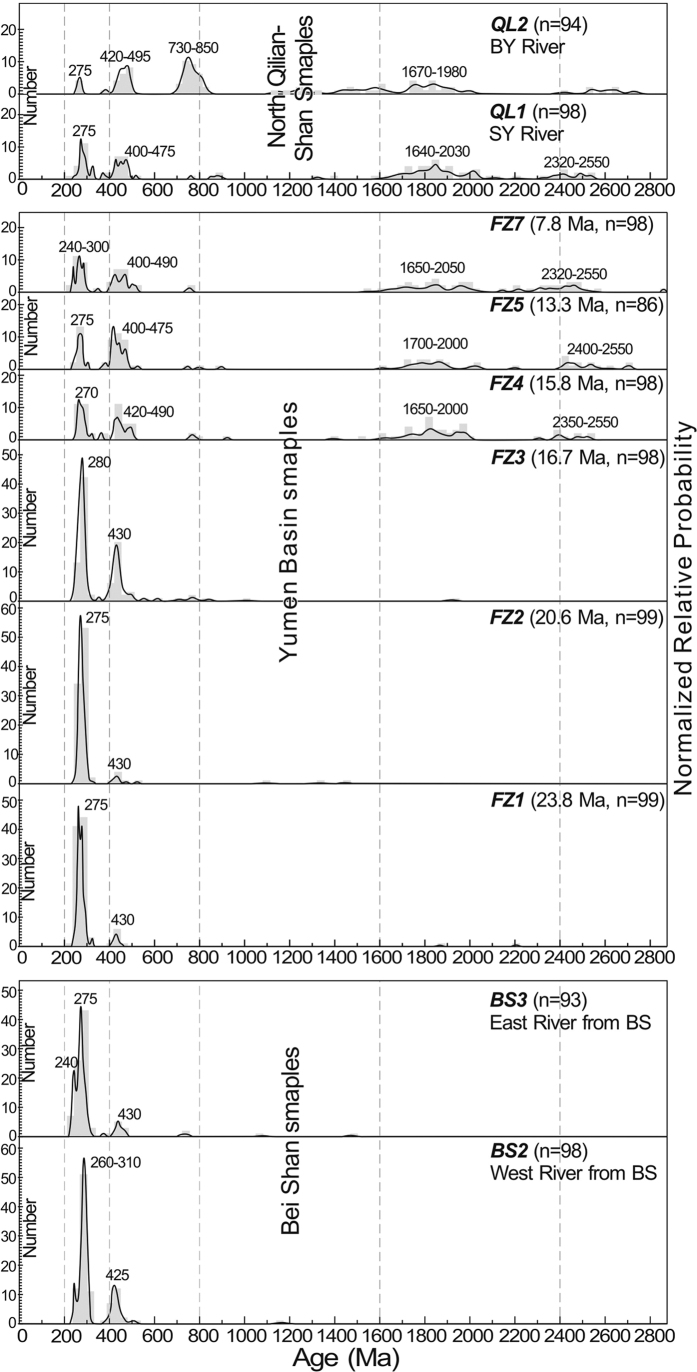
Age-probability plots of U-Pb ages of the detrital zircons from the Bei Shan (BS2-BS3), the North Qilian Shan (QL1-QL2), and the Caogou section in the Yumen Basin (FZ1-FZ5, FZ7).

Samples BS2 and BS3 were collected from two parallel rivers draining the Bei Shan (Fig. 1A). They are characterized by a U-Pb age population of 230–310 Ma, which constitutes 75% and 85% of the BS2 and the BS3 distributions, respectively. A few zircon ages at ~430 Ma were also recorded (Fig. 2). In the Bei Shan, the late Paleozoic and early Mesozoic granite plutons crop out widely, especially along its southern margins (Fig. 1A). Therefore, the 230–310 Ma population in the catchments definitely was eroded from these granite pluton source terranes. The ~430 Ma zircons may have eroded from a small area of early Paleozoic magmatic rocks present in the Bei Shan.

Samples QL1 and QL2 were collected from the SY and BY Rivers draining the North Qilian Shan (Fig. 1A). Four major age populations dominate the QL1 detrital zircon grains; there are peaks at 260–300 Ma, 415–485 Ma, 1640–2030 Ma, and 2320–2550 Ma (Fig. 2). In sample QL2, aside from the previously mentioned four age populations, 30% of the zircon grains date to 730–850 Ma, forming a fifth population (Fig. 2). Zircons yielding U-Pb ages at 1640–2030 Ma and 2320–2550 Ma are two unique zircon populations, which exhibit similar ages to the Proterozoic rocks cropping out along the modern northern Qilian Shan crest, suggesting these two groups of zircons were eroded from a Proterozoic source terrane in the North Qilian Shan^20^. These two populations in these proportions have not been identified in the Bei Shan. The other zircon populations of 260–300 Ma, 415–485 Ma, and 730–850 Ma may have been eroded from the corresponding plutons or strata in the North Qilian Shan.

Six samples from the Caogou section were dated, yielding more than 85 effective U-Pb dates for each of the samples. Zircons from 23.8 Ma (FZ1) and 20.6 Ma (FZ2) strata are dominated by age probability peaks at ~275 Ma, which account for >83% of the total dated grains. Ages older than 310 Ma only comprise ~15% of the population for both samples (Fig. 2). For sample (FZ3) from 16.7 Ma strata, ~90% of the zircon grains cluster at 240–310 Ma and 415–485 Ma; just a few grains yield ages older than 500 Ma. Zircon age populations of the three post-15.8 Ma samples (FZ4, FZ5 and FZ7) range from 235 Ma to 2880 Ma with four distinct peaks at ca. 275 Ma, 430 Ma, 1800 Ma, and 2450 Ma (Fig. 2). These distributions are totally different than the age distributions of pre-16.7 Ma strata.

To compare the similarity or dissimilarity of the detrital zircon age distributions, the Kolmogorov-Smirnoff (K-S) test was used. Two age distributions that returned high *P* values from the K-S test implies that the two age spectra are nearly identical. If the *P *< 0.05, then there is >95% level confidence that their age distributions are unlikely to have been derived from the same parent population.

From the K-S test, the 23.8–20.6 Ma samples and BS03 as well as the 16.7 Ma sample and BS02 have high resemblance, indicated by *P* > 0.1. However, these samples exhibit *P* values near zero with the post-15.8 Ma samples and the North Qilian Shan samples, suggesting a significant difference in the zircon age distribution patterns of the 24.0–16.7 Ma samples with post-15.8 Ma and the North Qilian Shan samples (Table 1). In contrast, the post-15.8 Ma samples had relatively high *P* values (0.093–0.932) from the K-S test with sample QL1, low *P* values (0–0.008) with sample QL2 and zero *P* values compared with the pre-15.8 Ma strata and the Bei Shan samples, again implying the dominant volume of zircons in the post-15.8 Ma samples are remarkably different than the pre-16.7 Ma and the Bei Shan samples. These results clearly reveal that the detrital zircons from the 24.0–16.7 Ma strata in the basin are locally derived from the Bei Shan source, whereas zircons from the post-15.8 Ma strata come from the North Qilian Shan source. A dramatic provenance change at ~16 Ma is also supported by the south-directed paleocurrent indicators shifting to north-directed ones since this time interval (Fig. 1B). The appearance of north-directed paleocurrent indicators provides independent evidence that North Qilian Shan-derived material entered the Yumen Basin during the middle Miocene.

## Implications and Discussion

Our detrital zircon data, combined with paleocurrent measurements, holds important information about the tectonic evolution of the northeastern Tibetan Plateau. Most of the clasts in the 24.0–16.7 Ma strata in the Yumen Basin from the Bei Shan sources indicate significant exhumation along the Bei Shan beginning at ~24 Ma. We interpreted this exhumation as a response to the onset of the rock uplift of the Bei Shan caused by tectonic deformation in the remote region of the northeastern Tibet. Although climate change can also trigger exhumation along mountain belts^21^, the coeval subsidence of the Yumen Basin along its southern margin provides strong evidence that the rock uplift and subsequent exhumation along the Bei Shan were driven by the tectonics in the region.

Our results do not support the hypothesis that the North Qilian Shan was deformed to form relatively high topography on the northeastern Tibetan Plateau before the middle Miocene, unlike the onset of the uplift of the Bei Shan at ~24 Ma. First, the absence of any detrital zircon grains from the North Qilian Shan in the pre-16 Ma strata is the most remarkable contrast in the detrital zircon age distributions (Fig. 2). The absence of these grains requires that the North Qilian Shan had relatively lower relief during the late Oligocene to early Miocene and was presumably tectonically inactive in the southern part of the basin. Further south of the Qilian Shan, the Qaidam Basin initiated as a result of thrust faulting-related subsidence along its northwestern margin during the Paleocene or Eocene and a wedge of conglomerate filled in the accommodation created by the subsidence^9^,^22^,^23^, suggesting that early Cenozoic high topography might lie in the North Qaidam-South Qilian Shan region. Second, the remnants of mid-Tertiary sediments are widespread in the North and Central Qilian Shan area without marginal facies^24^. Assuming that these sediments were part of the Hexi Corridor Basin, the North Qilian Shan seems to be covered by the southern extended Hexi Corridor Basin, again implying no significant deformation took place in the North Qilian Shan region.

A key result of the detrital zircon records is that the provenance of the Yumen Basin sharply changed from the Bei Shan to the North Qilian Shan at ~16 Ma. This result requires the rapid growth of the North Qilian Shan but the relatively steady uplift of the Bei Shan. Following the growth of the North Qilian Shan, the clasts eroded from the newly formed high topography of the North Qilian Shan replace the clasts from the Bei Shan forming the basin deposits. Thus, our results directly constrain the timing of the emergence of the North Qilian Shan to ~16 Ma, similar to the ages obtained from recent sedimentology and low-temperature thermochronology studies^11^,^25^,^26^,^27^,^28^.

The Cenozoic uplift histories of the Bei Shan and the North Qilian Shan challenge previous studies that infer the tectonic deformation in the Qilian Shan region was related to the systematic growth of the ATF northeastward since the late Miocene or the Pliocene^10^,^29^. Our results reveal poorly-known late Oligocene deformation in the outermost northeastern Tibetan Plateau, followed by the emergence of the North Qilian Shan in the mid-Miocene. During the late Oligocene, deformation was expressed as the rapid exhumation of the Bei Shan and subsidence of the Hexi Corridor Basin in the north, but the Qilian Shan, especially the North Qilian Shan was relative quiescent in the south. This observation indicates that late Oligocene deformation occurred prior to the ATF reaching this part of the plateau, which occurred relatively late in the history of the plateau. Although the geodynamics of the Oligocene deformation are not completely understood, it is consistent with the appearance of ranges and the formation of foreland basins along the northern and eastern margins of the Tibetan Plateau^30^,^31^, implying that stresses caused by the collision between India and Eurasia has been transferred to present-day northeastern Tibet since the late Oligocene. Thus, regardless of the exact mechanism, the tectonic processes along the Bei Shan-Hexi Corridor region may reflect far-field deformation associated with the Indo-Asian collision.

**Figure 3:**
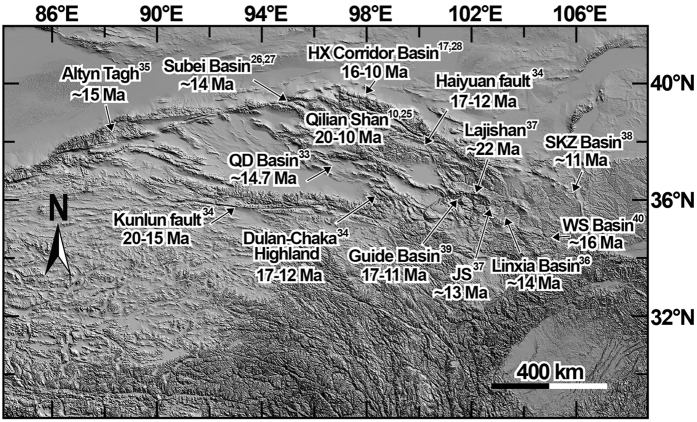
Miocene range growth, faulting and basin deformation throughout the northeastern Tibetan Plateau. The timing of the rapid exhumation of the ranges was based on Zheng *et al*.^10^, George *et al*.^25^, Duvall *et al*.^34^ and Ritts *et al*.^35^. Left-lateral strike-slip fault initiation ages were derived from Yuan *et al*.^6^ and Duvall *et al*.^34^. Changes in provenance /accumulation rates and basin formation/rotation were derived from Wang *et al*.^17^, Wang *et al*.^26^, Lin *et al*.^27^, Bovet *et al*.^28^, Fang *et al*.^33^, Garzione *et al*.^36^, Lease *et al*.^37^, Wang *et al*.^38^, Yan *et al*.^39^, Wang *et al*.^40^. The Fig. 3 was created by the Global Mapper 13 (http://www.bluemarblegeo.com/products/global-mapper-download.php) and CorelDRAW X7 (http://www.coreldraw.com/us/pages/free-download).

The mid-Miocene emergence of the North Qilian Shan could directly represent the most recent outward and upward growth of high topography in northeast Tibet. Yue *et al*.^32^ and Bovet *et al*.^28^ attribute the mid-Miocene uplift of the North Qilian Shan to the kinematic change along the ATF during the mid-Miocene. They suggest that the previous fast strike-slip movement along the ATF in northeast Tibet slowed down abruptly in the mid-Miocene, and renewed reverse faulting within the Qilian Shan region transferred the fault slip from the AFT to shortening and range growth. However, numerous studies have yielded evidence for emergence of the Qilian Shan occurring almost simultaneously along its southern to northern parts at 16–10 Ma, such as magnetostratigraphic dating of sediments to derive sediment accumulation rates or provenance changes in the Qaidam Basin^33^, Subei Basin^26^,^27^, Hexi Corridor Basin^17^,^28^; and low-temperature thermochronology dating of detrital minerals to define rapid exhumation of the adjacent ranges^11^,^25^ (Fig. 3). With the exception of the Qilian Shan, middle Miocene range growth and deformation appear to be widespread across the northern Tibetan Plateau. For example, thermochronologic data reveal that the onset of left-lateral strike slip motion on the Kunlun Fault initiated 20–15 Ma, the Haiyuan Fault initiated 17–12 Ma and rapid exhumation of the Dulan-Chaka highlands occurred at ~15 Ma^34^. Foraminifera assemblages contain planktonic taxa coeval with the apatite fission track, and (U-Th)/He thermal models clearly show that the Altyn Tagh has undergone significant exhumation and surface uplift from sea level to its present elevation of 1400 m since ~15 Ma^35^. Moreover, sedimentology studies constrained by paleomagnetism document changes in provenance in the Linxia Basin at ~14 Ma^36^, the Xunhua Basin at ~22 Ma^37^, sediment accumulation rate increases in the Sikouzi Basin at ~11 Ma^38^, in the Xunhua Basin at ~13 Ma^37^, the clockwise rotation of the Guide Basin at 17–11 Ma^39^ and the formation of the Wushan pull-apart Basin at ~16 Ma^40^, indicating deformation occurred throughout the region during the mid-Miocene (Fig. 3). Such widespread synchronous activity supports an alternative interpretation that convective removal of some gravitationally unstable mantle lithosphere beneath north-central Tibet could trigger or accelerate deformation on the plateau margins^41^. The mantle lithosphere removal could augment the potential energy per unit area and then transfer the deformation to the plateau surroundings. In the northeastern Tibet, the Qaidam Basin is a relatively rigid block that is efficient at transferring deformation north and east to the Qilian Shan and elsewhere. During this process, the ATF may act as a transfer fault to accommodate deformation through northeastward propagation, and the termination of the ATF in the Yumen Basin may delimit the boundary of deformation caused by mid-Miocene mantle removal. This may also be the reason why the Bei Shan was inactive after the mid-Miocene.

## Conclusion

The different provenance histories of the sediments in the Cenozoic Yumen Basin revealed by the detrital zircons and paleocurrent measurements provide a new perspective on the deformation along the outermost region of the northeastern Tibetan Plateau. The zircon grains from the 24.2–16.7 Ma strata in the basin are dominated by the Bei Shan plutons and indicate the late Oligocene uplift of the Bei Shan that may reflect the far-field deformation related to the Indo-Asian collision. The dramatic provenance change at ~16 Ma is highlighted by the North Qilian Shan sources, suggesting the onset of the rapid uplift of the range at this time as a result of the decreased slip rates on the ATF or in response to the removal of lithospheric mantle beneath north-central Tibet.

## Additional Information

**How to cite this article**: Wang, W. *et al*. Constraints on mountain building in the northeastern Tibet: Detrital zircon records from synorogenic deposits in the Yumen Basin. *Sci. Rep.* **6**, 27604; doi: 10.1038/srep27604 (2016).

## Supplementary Material

supplementary Fig.1 and supplementary Table 1

## Figures and Tables

**Table 1:**
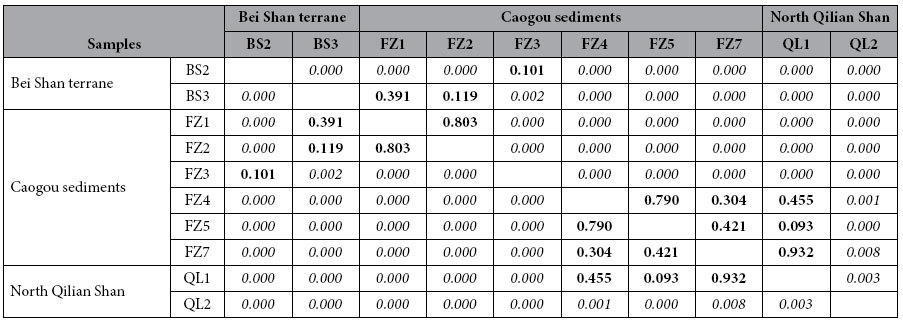
*P* values for the K-S comparison of the isotopic ages of the detrital zircons from sands draining the Bei Shan (BS2-BS3), North Qilian Shan (QL1-QL2) and sandstones from the Yumen Basin (FZ1-FZ5, FZ7).

Two statistically distinct groups of the samples are recognized based on the P values. P values greater than 0.05 are considered to be statistically indistinguishable, and P values of 0 have virtual certainty of different parent populations.
