# Supplementary material for: Constraints on mountain building in the northeastern Tibet: Detrital zircon records from synorogenic deposits in the Yumen Basin
Source: Sci Rep. 2016 Jun 9;6:27604. doi: 10.1038/srep27604 (PMC4899710; doi:10.1038/srep27604)
Supplement: supplementary Fig.1 and supplementary Table 1 [file srep27604-s1.pdf]

Constraints on mountain building in northeast Tibet: Detrital zircon records from synorogenic deposits in the Yumen Basin

Weitao Wang*, Zhang Peizhen, Yu Jinxing, Wang Yizhou, Zheng Dewen, Zheng Wenjun, Zhang Huiping, Pang Jianzhang

State Key Laboratory of Earthquake Dynamics, Institute of Geology, China Earthquake Administration, Beijing, China

Figure caption for supplementary Fig.1

Supplementary Fig.1 Depositional ages of detrital zircon samples from the Magnetostratigrpahic Caogou section[1]. Based on the Magnetostratigrpahy, the depositional ages of six detrital zircon samples (FZ1-FZ5, FZ7) are 23.8 Ma, 20.6 Ma, 16.7 Ma, 15.8 Ma, 13.2 Ma and 7.5 Ma.

Supplementary Table 1 U-Pb (zircon) geochronologic analyses by LA-ICP-MS

Reference

1 Wang, W., *et al.* Revisiting the Cenozoic uplift of the Qilian Shan in the northeastern Tibetan Plateau: Evidence for sedimentary archive from the Jiuxi basin. *Journal of Geophysical Research* in press.

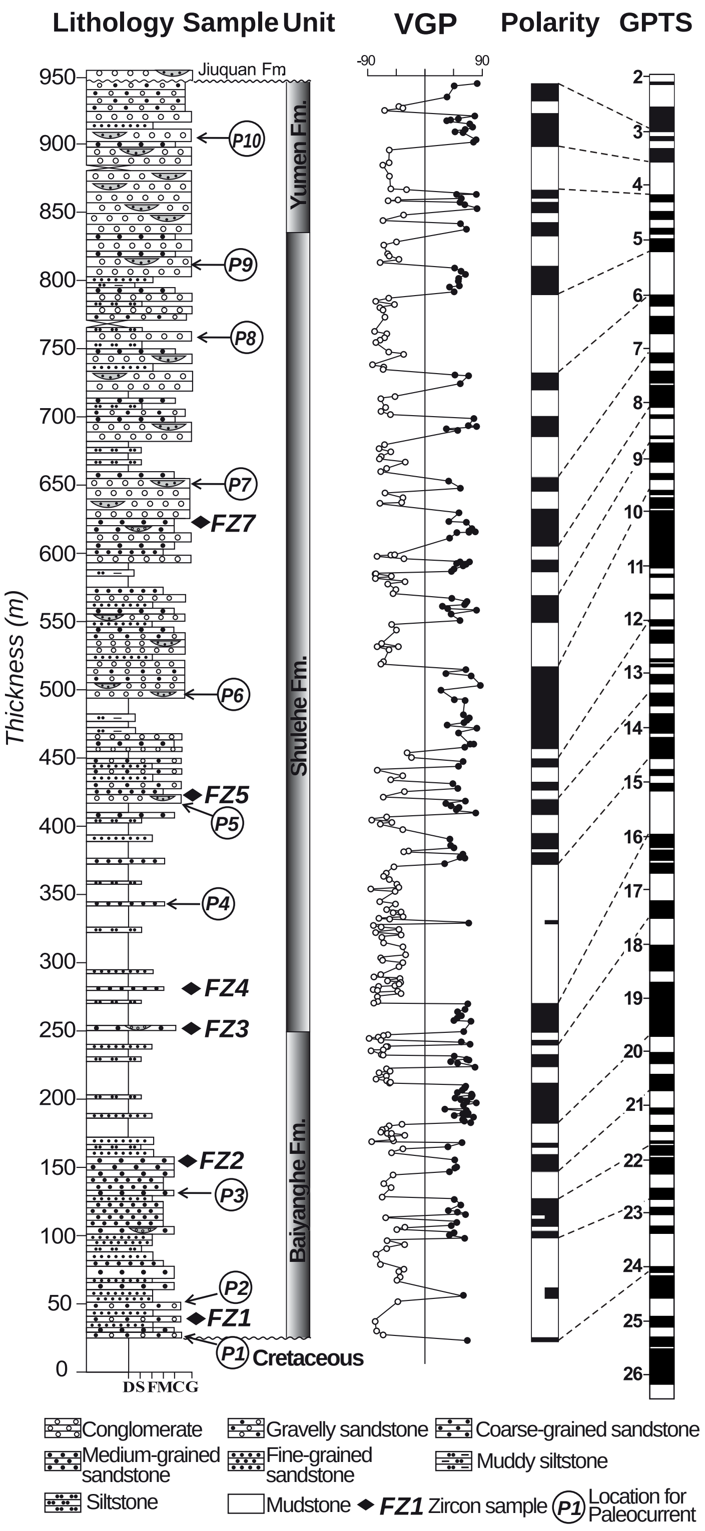
Lithology
Sample
Unit
VGP
Polarity
GPTS
Thickness (m)
Jiuquan Fm
Yumen Fm.
Shulehe Fm.
Baiyanghe Fm.
Cretaceous
P1
P2
P3
P4
P5
P6
P7
P8
P9
P10
FZ1
FZ2
FZ3
FZ4
FZ5
FZ7
D S F M C G
-90
90
Conglomerate
Gravelly sandstone
Coarse-grained sandstone
Medium-grained sandstone
Fine-grained sandstone
Muddy siltstone
Siltstone
Mudstone
FZ1 Zircon sample
P1 Location for Paleocurrent

Supplementary Table 1 U-Pb(Zircon) geochronologic analyses by LA-ICP-MS

Sample (Grain)	Th (ppm)	U (ppm)	$\frac{Th}{U}$	**Isotopic ratios**						**Apparent age (Ma)**						**Corr. disco (%)**	**Best Age (Ma)**	1σ
				$\frac{^{207}Pb}{^{206}Pb}$	1σ	$\frac{^{207}Pb}{^{235}U}$	1σ	$\frac{^{206}Pb}{^{238}U}$	1σ	$\frac{^{207}Pb}{^{206}Pb}$	1σ	$\frac{^{207}Pb}{^{235}U}$	1σ	$\frac{^{206}Pb}{^{238}U}$	1σ			
BS2 026	248	337	0.7	0.05526	0.00299	0.29236	0.01441	0.03835	0.00104	422.7	115.9	260.4	11.3	242.6	6.4	-6.8	242.6	6.4
BS2 019	417	863	0.5	0.05681	0.00126	0.30106	0.00590	0.03845	0.00064	483.5	48.5	267.2	4.6	243.2	4.0	-9.0	243.2	4.0
BS2 074	188	207	0.9	0.05445	0.00142	0.28961	0.00709	0.03858	0.00091	389.5	57.1	258.2	5.6	244.1	5.7	-5.5	244.1	5.7
BS2 027	572	1177	0.5	0.05117	0.00119	0.27461	0.00571	0.03890	0.00066	248.6	52.7	246.4	4.6	246.0	4.1	-0.2	246.0	4.1
BS2 028	194	568	0.3	0.05454	0.00274	0.29367	0.01336	0.03903	0.00102	393.2	108.3	261.4	10.5	246.8	6.3	-5.6	246.8	6.3
BS2 011	208	674	0.3	0.05303	0.00142	0.28532	0.00687	0.03906	0.00068	329.9	59.8	254.9	5.4	247.0	4.2	-3.1	247.0	4.2
BS2 021	365	687	0.5	0.06168	0.00211	0.33852	0.01035	0.03981	0.00083	662.9	71.8	296.1	7.9	251.6	5.1	-15.0	251.6	5.1
BS2 018	232	596	0.4	0.05301	0.00276	0.29418	0.01395	0.04026	0.00106	329.2	113.7	261.8	10.9	254.5	6.6	-2.8	254.5	6.6
BS2 068	122	204	0.6	0.06057	0.00161	0.33915	0.00839	0.04061	0.00097	623.9	56.2	296.5	6.4	256.6	6.0	-13.5	256.6	6.0
BS2 030	350	868	0.4	0.05552	0.00173	0.31882	0.00894	0.04162	0.00081	433.1	67.8	281.0	6.9	262.9	5.0	-6.4	262.9	5.0
BS2 017	437	596	0.7	0.05717	0.00243	0.33475	0.01284	0.04249	0.00100	497.5	91.7	293.2	9.8	268.2	6.2	-8.5	268.2	6.2
BS2 002	155	664	0.2	0.05446	0.00125	0.31973	0.00655	0.04261	0.00075	389.9	50.3	281.7	5.0	269.0	4.7	-4.5	269.0	4.7
BS2 049	74	99	0.7	0.05790	0.00252	0.34219	0.01367	0.04286	0.00122	525.8	93.0	298.8	10.3	270.5	7.5	-9.5	270.5	7.5
~~BS2 012~~	~~378~~	~~786~~	~~0.5~~	~~0.08685~~	~~0.00148~~	~~0.51317~~	~~0.00730~~	~~0.04289~~	~~0.00068~~	~~1357.1~~	~~32.6~~	~~420.6~~	~~4.9~~	~~270.7~~	~~4.2~~	~~-35.6~~	~~270.7~~	~~4.2~~
BS2 013	376	725	0.5	0.05428	0.00177	0.32330	0.00949	0.04323	0.00084	382.4	71.5	284.4	7.3	272.8	5.2	-4.1	272.8	5.2
BS2 016	300	407	0.7	0.05412	0.00375	0.32406	0.02052	0.04345	0.00143	376.0	148.6	285.0	15.7	274.2	8.9	-3.8	274.2	8.9
BS2 034	275	270	1.0	0.05475	0.00141	0.33195	0.00806	0.04396	0.00103	402.1	56.5	291.1	6.1	277.3	6.4	-4.7	277.3	6.4
BS2 054	331	306	1.1	0.05901	0.00136	0.35857	0.00778	0.04406	0.00102	567.6	49.4	311.1	5.8	278.0	6.3	-10.6	278.0	6.3
BS2 022	440	848	0.5	0.05510	0.00200	0.33623	0.01099	0.04425	0.00094	416.1	78.6	294.3	8.4	279.1	5.8	-5.2	279.1	5.8
BS2 081	254	245	1.0	0.05892	0.00179	0.35940	0.01013	0.04425	0.00110	564.1	64.9	311.8	7.6	279.1	6.8	-10.5	279.1	6.8
~~BS2 023~~	~~136~~	~~213~~	~~0.6~~	~~0.08811~~	~~0.00312~~	~~0.53884~~	~~0.01657~~	~~0.04435~~	~~0.00101~~	~~1384.9~~	~~66.4~~	~~437.7~~	~~10.9~~	~~279.7~~	~~6.3~~	~~-36.1~~	~~279.7~~	~~6.3~~
BS2 052	343	303	1.1	0.05623	0.00130	0.34580	0.00754	0.04460	0.00103	460.8	50.8	301.6	5.7	281.3	6.4	-6.7	281.3	6.4
BS2 053	166	221	0.8	0.05478	0.00142	0.33941	0.00824	0.04493	0.00106	403.4	56.1	296.7	6.3	283.3	6.6	-4.5	283.3	6.6
BS2 063	303	340	0.9	0.05494	0.00108	0.34028	0.00643	0.04493	0.00101	409.6	43.1	297.4	4.9	283.3	6.2	-4.7	283.3	6.2
BS2 039	197	292	0.7	0.05820	0.00150	0.36102	0.00873	0.04498	0.00106	536.8	56.5	313.0	6.5	283.6	6.6	-9.4	283.6	6.6
BS2 037	129	165	0.8	0.05491	0.00202	0.34079	0.01164	0.04500	0.00118	408.5	79.8	297.8	8.8	283.8	7.3	-4.7	283.8	7.3
BS2 014	437	765	0.6	0.06282	0.00287	0.38987	0.01597	0.04504	0.00114	702.2	94.5	334.3	11.7	284.0	7.0	-15.0	284.0	7.0
BS2 031	98	132	0.7	0.05293	0.00191	0.32918	0.01104	0.04509	0.00116	325.7	79.9	288.9	8.4	284.3	7.2	-1.6	284.3	7.2

BS2 061	132	146	0.9	0.05901	0.00347	0.36710	0.01965	0.04512	0.00152	567.6	123.0	317.5	14.6	284.5	9.4	-10.4	284.5	9.4
BS2 045	273	446	0.6	0.05659	0.00106	0.35255	0.00637	0.04517	0.00101	475.0	41.4	306.6	4.8	284.8	6.2	-7.1	284.8	6.2
BS2 070	176	232	0.8	0.05502	0.00225	0.34289	0.01296	0.04521	0.00124	412.8	88.5	299.4	9.8	285.0	7.6	-4.8	285.0	7.6
BS2 033	299	304	1.0	0.05779	0.00125	0.36037	0.00738	0.04522	0.00103	521.4	47.0	312.5	5.5	285.1	6.4	-8.8	285.1	6.4
BS2 043	178	192	0.9	0.05358	0.00148	0.33560	0.00871	0.04542	0.00109	353.1	61.3	293.8	6.6	286.4	6.7	-2.5	286.4	6.7
BS2 035	289	264	1.1	0.05623	0.00165	0.35327	0.00965	0.04556	0.00111	460.5	64.2	307.2	7.2	287.2	6.9	-6.5	287.2	6.9
BS2 064	199	314	0.6	0.05403	0.00138	0.33949	0.00814	0.04557	0.00107	372.3	56.2	296.8	6.2	287.3	6.6	-3.2	287.3	6.6
BS2 050	197	254	0.8	0.05949	0.00147	0.37442	0.00865	0.04564	0.00107	585.2	52.6	322.9	6.4	287.7	6.6	-10.9	287.7	6.6
BS2 058	146	170	0.9	0.05851	0.00325	0.36850	0.01869	0.04568	0.00148	548.8	117.0	318.5	13.9	287.9	9.2	-9.6	287.9	9.2
BS2 079	143	159	0.9	0.05380	0.00183	0.33889	0.01073	0.04569	0.00116	362.5	74.8	296.3	8.1	288.0	7.2	-2.8	288.0	7.2
BS2 038	263	256	1.0	0.06384	0.00187	0.40274	0.01089	0.04574	0.00113	736.5	60.9	343.6	7.9	288.3	7.0	-16.1	288.3	7.0
BS2 032	134	163	0.8	0.05533	0.00184	0.34944	0.01080	0.04579	0.00116	425.4	72.3	304.3	8.1	288.6	7.1	-5.2	288.6	7.1
BS2 059	154	219	0.7	0.05382	0.00141	0.34009	0.00838	0.04582	0.00109	363.6	58.0	297.2	6.4	288.8	6.7	-2.8	288.8	6.7
BS2 069	173	280	0.6	0.05711	0.00159	0.36087	0.00939	0.04583	0.00111	495.4	60.9	312.9	7.0	288.9	6.8	-7.7	288.9	6.8
BS2 078	230	249	0.9	0.05446	0.00121	0.34431	0.00725	0.04586	0.00105	389.9	48.7	300.4	5.5	289.1	6.5	-3.8	289.1	6.5
BS2 040	191	195	1.0	0.05824	0.00186	0.36874	0.01092	0.04591	0.00115	538.3	69.0	318.7	8.1	289.3	7.1	-9.2	289.3	7.1
BS2 060	144	204	0.7	0.05395	0.00151	0.34179	0.00897	0.04595	0.00111	368.7	61.8	298.5	6.8	289.6	6.8	-3.0	289.6	6.8
BS2 046	158	205	0.8	0.05426	0.00161	0.34389	0.00952	0.04596	0.00112	381.8	65.0	300.1	7.2	289.7	6.9	-3.5	289.7	6.9
BS2 036	128	151	0.9	0.05553	0.00194	0.35218	0.01141	0.04599	0.00118	433.2	75.9	306.4	8.6	289.8	7.3	-5.4	289.8	7.3
BS2 024	371	899	0.4	0.05501	0.00326	0.34918	0.01884	0.04603	0.00137	412.6	126.9	304.1	14.2	290.1	8.5	-4.6	290.1	8.5
BS2 057	173	182	1.0	0.05698	0.00208	0.36228	0.01223	0.04611	0.00122	490.3	79.3	313.9	9.1	290.6	7.5	-7.4	290.6	7.5
BS2 051	165	160	1.0	0.05593	0.00206	0.35636	0.01212	0.04621	0.00121	449.2	79.6	309.5	9.1	291.2	7.5	-5.9	291.2	7.5
BS2 086	396	339	1.2	0.05520	0.00131	0.35173	0.00789	0.04623	0.00108	420.2	51.5	306.0	5.9	291.3	6.6	-4.8	291.3	6.6
BS2 071	265	270	1.0	0.05356	0.00122	0.34141	0.00740	0.04624	0.00106	352.4	50.8	298.2	5.6	291.4	6.6	-2.3	291.4	6.6
BS2 029	179	345	0.5	0.06218	0.00296	0.39824	0.01715	0.04643	0.00119	680.2	98.7	340.4	12.5	292.5	7.4	-14.1	292.5	7.4
BS2 096	78	108	0.7	0.05548	0.00198	0.35497	0.01173	0.04642	0.00121	431.4	77.5	308.5	8.8	292.5	7.5	-5.2	292.5	7.5
BS2 087	108	139	0.8	0.05634	0.00298	0.36175	0.01755	0.04658	0.00146	465.1	113.9	313.5	13.1	293.5	9.0	-6.4	293.5	9.0
~~BS2 041~~	~~119~~	~~157~~	~~0.8~~	~~0.07743~~	~~0.00195~~	~~0.49760~~	~~0.01149~~	~~0.04660~~	~~0.00112~~	~~1132.2~~	~~49.4~~	~~410.1~~	~~7.8~~	~~293.6~~	~~6.9~~	~~-28.4~~	~~293.6~~	~~6.9~~
~~BS2 065~~	~~129~~	~~174~~	~~0.7~~	~~0.07337~~	~~0.00169~~	~~0.47554~~	~~0.01016~~	~~0.04701~~	~~0.00111~~	~~1024.3~~	~~45.8~~	~~395.0~~	~~7.0~~	~~296.1~~	~~6.8~~	~~-25.0~~	~~296.1~~	~~6.8~~
BS2 048	128	138	0.9	0.05331	0.00220	0.34611	0.01322	0.04708	0.00129	341.9	90.7	301.8	10.0	296.6	7.9	-1.7	296.6	7.9
BS2 097	314	315	1.0	0.05325	0.00108	0.34558	0.00672	0.04709	0.00107	339.3	45.2	301.4	5.1	296.6	6.6	-1.6	296.6	6.6

BS2 094	140	166	0.8	0.05468	0.00349	0.35501	0.02074	0.04711	0.00165	399.0	136.4	308.5	15.5	296.8	10.2	-3.8	296.8	10.2
BS2 077	152	313	0.5	0.05594	0.00134	0.36358	0.00823	0.04715	0.00110	449.5	52.3	314.9	6.1	297.0	6.8	-5.7	297.0	6.8
BS2 083	163	168	1.0	0.05237	0.00167	0.34042	0.01012	0.04716	0.00118	301.5	70.8	297.5	7.7	297.0	7.2	-0.2	297.0	7.2
BS2 067	158	185	0.9	0.05563	0.00157	0.36198	0.00957	0.04719	0.00114	437.4	61.5	313.7	7.1	297.3	7.0	-5.2	297.3	7.0
BS2 085	216	351	0.6	0.05542	0.00157	0.36055	0.00956	0.04719	0.00115	429.1	61.7	312.6	7.1	297.3	7.1	-4.9	297.3	7.1
BS2 062	153	178	0.9	0.05619	0.00171	0.36613	0.01041	0.04726	0.00117	459.3	66.7	316.8	7.7	297.6	7.2	-6.1	297.6	7.2
BS2 098	83	126	0.7	0.06424	0.00235	0.41885	0.01402	0.04731	0.00127	749.5	75.4	355.2	10.0	298.0	7.8	-16.1	298.0	7.8
BS2 102	151	182	0.8	0.05943	0.00245	0.38925	0.01470	0.04752	0.00133	582.9	87.0	333.8	10.8	299.3	8.2	-10.3	299.3	8.2
BS2 090	119	138	0.9	0.05483	0.00170	0.36006	0.01041	0.04765	0.00119	405.1	67.3	312.3	7.8	300.1	7.3	-3.9	300.1	7.3
BS2 095	147	185	0.8	0.05590	0.00137	0.36774	0.00849	0.04773	0.00112	448.1	53.4	318.0	6.3	300.6	6.9	-5.5	300.6	6.9
~~BS2 055~~	~~165~~	~~174~~	~~0.9~~	~~0.07415~~	~~0.00186~~	~~0.48850~~	~~0.01125~~	~~0.04778~~	~~0.00115~~	~~1045.6~~	~~49.7~~	~~403.9~~	~~7.7~~	~~300.9~~	~~7.1~~	~~-25.5~~	~~300.9~~	~~7.1~~
BS2 015	226	334	0.7	0.06661	0.00169	0.43925	0.00980	0.04786	0.00086	825.5	52.1	369.7	6.9	301.4	5.3	-18.5	301.4	5.3
BS2 105	122	154	0.8	0.05600	0.00162	0.36956	0.01000	0.04788	0.00118	452.2	63.2	319.3	7.4	301.5	7.2	-5.6	301.5	7.2
BS2 025	217	301	0.7	0.05349	0.00107	0.35464	0.00680	0.04811	0.00109	349.6	44.5	308.2	5.1	302.9	6.7	-1.7	302.9	6.7
BS2 010	175	209	0.8	0.05268	0.00128	0.34935	0.00805	0.04812	0.00113	315.0	54.3	304.2	6.1	303.0	6.9	-0.4	303.0	6.9
BS2 092	118	133	0.9	0.05403	0.00240	0.36050	0.01479	0.04840	0.00138	372.3	96.5	312.6	11.0	304.7	8.5	-2.5	304.7	8.5
BS2 091	186	218	0.9	0.05447	0.00123	0.36467	0.00780	0.04857	0.00112	390.5	49.4	315.7	5.8	305.7	6.9	-3.2	305.7	6.9
BS2 103	212	391	0.5	0.05866	0.00118	0.39414	0.00755	0.04875	0.00111	554.5	43.2	337.4	5.5	306.9	6.8	-9.0	306.9	6.8
BS2 101	108	141	0.8	0.05620	0.00174	0.37779	0.01092	0.04877	0.00122	459.6	67.8	325.4	8.1	307.0	7.5	-5.7	307.0	7.5
BS2 088	174	186	0.9	0.05512	0.00199	0.37453	0.01255	0.04929	0.00129	417.0	78.4	323.0	9.3	310.2	7.9	-4.0	310.2	7.9
~~BS2 089~~	~~117~~	~~165~~	~~0.7~~	~~0.20319~~	~~0.00320~~	~~1.63795~~	~~0.02330~~	~~0.05848~~	~~0.00135~~	~~2852.0~~	~~25.4~~	~~984.9~~	~~9.0~~	~~366.4~~	~~8.2~~	~~-62.8~~	~~366.4~~	~~8.2~~
~~BS2 006~~	~~111~~	~~142~~	~~0.8~~	~~0.17229~~	~~0.00518~~	~~1.43244~~	~~0.03436~~	~~0.06034~~	~~0.00147~~	~~2580.0~~	~~49.3~~	~~902.6~~	~~14.3~~	~~377.7~~	~~9.0~~	~~-58.2~~	~~377.7~~	~~9.0~~
BS2 009	89	208	0.4	0.07057	0.00201	0.60470	0.01524	0.06219	0.00125	945.0	57.2	480.2	9.6	388.9	7.6	-19.0	388.9	7.6
BS2 004	70	241	0.3	0.06164	0.00149	0.55961	0.01273	0.06588	0.00156	661.7	51.1	451.3	8.3	411.3	9.5	-8.9	411.3	9.5
BS2 047	103	992	0.1	0.05553	0.00073	0.50579	0.00676	0.06605	0.00142	433.4	28.5	415.6	4.6	412.3	8.6	-0.8	412.3	8.6
BS2 056	109	219	0.5	0.06080	0.00123	0.55612	0.01073	0.06634	0.00151	632.0	43.0	449.0	7.0	414.1	9.1	-7.8	414.1	9.1
BS2 066	99	262	0.4	0.05845	0.00115	0.53639	0.01008	0.06656	0.00150	546.7	42.3	436.0	6.7	415.4	9.1	-4.7	415.4	9.1
BS2 044	83	150	0.6	0.05941	0.00141	0.54648	0.01222	0.06670	0.00156	582.3	50.7	442.7	8.0	416.2	9.4	-6.0	416.2	9.4
BS2 075	142	618	0.2	0.05914	0.00094	0.54627	0.00853	0.06701	0.00147	572.1	34.1	442.6	5.6	418.1	8.9	-5.5	418.1	8.9
BS2 073	97	341	0.3	0.05707	0.00096	0.52742	0.00864	0.06704	0.00148	493.5	36.7	430.1	5.8	418.3	9.0	-2.7	418.3	9.0
BS2 076	121	257	0.5	0.05774	0.00121	0.53765	0.01073	0.06754	0.00154	519.7	45.5	436.9	7.1	421.3	9.3	-3.6	421.3	9.3

BS2 072	136	549	0.2	0.05610	0.00080	0.52317	0.00750	0.06765	0.00147	455.9	31.1	427.3	5.0	422.0	8.9	-1.2	422.0	8.9
BS2 080	152	505	0.3	0.05954	0.00090	0.55533	0.00835	0.06766	0.00148	587.0	32.6	448.5	5.5	422.0	9.0	-5.9	422.0	9.0
BS2 099	107	224	0.5	0.05882	0.00120	0.55701	0.01088	0.06871	0.00157	560.5	43.9	449.6	7.1	428.4	9.5	-4.7	428.4	9.5
BS2 084	25	1045	0.0	0.06117	0.00074	0.58244	0.00729	0.06908	0.00149	645.1	25.7	466.0	4.7	430.6	9.0	-7.6	430.6	9.0
BS2 042	147	345	0.4	0.05879	0.00100	0.56138	0.00932	0.06925	0.00153	559.2	36.1	452.4	6.1	431.6	9.2	-4.6	431.6	9.2
BS2 104	87	303	0.3	0.05546	0.00102	0.53182	0.00952	0.06959	0.00156	430.4	40.1	433.0	6.3	433.7	9.4	0.2	433.7	9.4
BS2 082	141	254	0.6	0.05838	0.00106	0.56337	0.00994	0.07001	0.00157	544.0	39.3	453.7	6.5	436.2	9.4	-3.9	436.2	9.4
BS2 007	45	205	0.2	0.05819	0.00234	0.56216	0.02056	0.07012	0.00165	536.2	86.3	452.9	13.4	436.9	9.9	-3.5	436.9	9.9
BS2 093	113	345	0.3	0.06377	0.00138	0.61697	0.01262	0.07019	0.00163	734.0	45.1	487.9	7.9	437.3	9.8	-10.4	437.3	9.8
BS2 008	140	273	0.5	0.05714	0.00102	0.56604	0.00981	0.07189	0.00161	496.2	38.8	455.5	6.4	447.5	9.7	-1.8	447.5	9.7
BS2 003	104	194	0.5	0.05853	0.00122	0.58131	0.01155	0.07207	0.00165	549.7	44.6	465.3	7.4	448.6	9.9	-3.6	448.6	9.9
BS2 005	142	360	0.4	0.06436	0.00164	0.65694	0.01491	0.07408	0.00141	753.4	52.8	512.7	9.1	460.7	8.4	-10.1	460.7	8.4
BS2 020	150	251	0.6	0.05861	0.00449	0.62374	0.04364	0.07721	0.00299	552.5	159.1	492.2	27.3	479.4	17.9	-2.6	479.4	17.9
BS2 100	290	1392	0.2	0.06092	0.00070	0.69407	0.00840	0.08266	0.00178	636.4	24.6	535.2	5.0	512.0	10.6	-4.3	512.0	10.6
BS2 001	153	475	0.3	0.07884	0.00106	2.10933	0.02456	0.19416	0.00312	1168.2	26.5	1151.9	8.0	1143.9	16.9	-0.7	1168.2	26.5

BS3 093	109	269	0.4	0.05119	0.00213	0.25752	0.00988	0.03650	0.00095	249.3	92.9	232.7	8.0	231.1	5.9	0.7	231.1	5.9
BS3 094	128	391	0.3	0.05194	0.00131	0.26320	0.00618	0.03676	0.00081	282.7	56.5	237.2	5.0	232.7	5.0	1.9	232.7	5.0
BS3 039	210	359	0.6	0.06552	0.00231	0.33566	0.01070	0.03717	0.00093	791.2	72.2	293.9	8.1	235.3	5.8	19.9	235.3	5.8
BS3 100	237	253	0.9	0.05456	0.00188	0.27987	0.00890	0.03721	0.00090	394.2	74.7	250.6	7.1	235.5	5.6	6.0	235.5	5.6
BS3 065	149	350	0.4	0.05630	0.00189	0.28938	0.00892	0.03729	0.00090	463.6	72.8	258.1	7.0	236.0	5.6	8.6	236.0	5.6
BS3 070	199	228	0.9	0.05375	0.00223	0.27855	0.01066	0.03760	0.00097	360.5	90.7	249.5	8.5	237.9	6.1	4.6	237.9	6.1
BS3 058	111	271	0.4	0.06503	0.00201	0.33883	0.00956	0.03780	0.00089	775.5	63.6	296.3	7.3	239.2	5.5	19.3	239.2	5.5
BS3 047	202	315	0.6	0.05093	0.00191	0.26685	0.00929	0.03802	0.00094	237.5	84.4	240.2	7.4	240.5	5.8	-0.1	240.5	5.8
BS3 082	106	328	0.3	0.05123	0.00153	0.26841	0.00746	0.03801	0.00087	251.3	67.3	241.4	6.0	240.5	5.4	0.4	240.5	5.4
BS3 088	230	228	1.0	0.05792	0.00287	0.30360	0.01371	0.03803	0.00111	526.3	105.4	269.2	10.7	240.6	6.9	10.6	240.6	6.9
BS3 075	123	141	0.9	0.05431	0.00277	0.28556	0.01342	0.03814	0.00110	384.0	110.4	255.1	10.6	241.3	6.9	5.4	241.3	6.9
BS3 067	226	667	0.3	0.05365	0.00125	0.28226	0.00613	0.03817	0.00082	356.1	51.7	252.4	4.9	241.5	5.1	4.3	241.5	5.1
~~BS3 076~~	~~150~~	~~143~~	~~1.0~~	~~0.08178~~	~~0.00321~~	~~0.43039~~	~~0.01507~~	~~0.03818~~	~~0.00104~~	~~1240.3~~	~~75.0~~	~~363.5~~	~~10.7~~	~~241.5~~	~~6.5~~	~~33.6~~	~~241.5~~	~~6.5~~
BS3 084	104	420	0.2	0.05294	0.00140	0.27924	0.00688	0.03826	0.00085	326.3	58.9	250.1	5.5	242.1	5.3	3.2	242.1	5.3
BS3 099	228	580	0.4	0.06422	0.00203	0.33989	0.00978	0.03839	0.00093	749.0	65.4	297.1	7.4	242.9	5.8	18.2	242.9	5.8

BS3 089	141	207	0.7	0.05488	0.00221	0.29054	0.01079	0.03841	0.00098	407.2	87.0	259.0	8.5	243.0	6.1	6.2	243.0	6.1
BS3 041	441	359	1.2	0.05543	0.00197	0.29406	0.00964	0.03849	0.00094	429.2	77.3	261.8	7.6	243.5	5.8	7.0	243.5	5.8
BS3 042	164	291	0.6	0.06457	0.00346	0.34356	0.01664	0.03861	0.00120	760.3	109.1	299.9	12.6	244.2	7.5	18.6	244.2	7.5
~~BS3 045~~	~~123~~	~~106~~	~~1.2~~	~~0.07734~~	~~0.00369~~	~~0.42345~~	~~0.01809~~	~~0.03973~~	~~0.00118~~	~~1129.9~~	~~92.2~~	~~358.5~~	~~12.9~~	~~251.1~~	~~7.3~~	~~30.0~~	~~251.1~~	~~7.3~~
BS3 048	143	215	0.7	0.05367	0.00203	0.29456	0.01028	0.03982	0.00099	357.1	82.9	262.1	8.1	251.7	6.1	4.0	251.7	6.1
~~BS3 051~~	~~99~~	~~96~~	~~1.0~~	~~0.09215~~	~~0.00455~~	~~0.50575~~	~~0.02195~~	~~0.03982~~	~~0.00126~~	~~1470.4~~	~~91.1~~	~~415.6~~	~~14.8~~	~~251.7~~	~~7.8~~	~~39.4~~	~~251.7~~	~~7.8~~
BS3 083	171	284	0.6	0.05297	0.00226	0.29194	0.01147	0.03998	0.00106	327.6	93.8	260.1	9.0	252.7	6.6	2.8	252.7	6.6
BS3 086	258	590	0.4	0.05608	0.00122	0.30999	0.00630	0.04010	0.00086	455.2	47.4	274.2	4.9	253.5	5.3	7.5	253.5	5.3
BS3 087	230	501	0.5	0.05491	0.00133	0.30601	0.00693	0.04043	0.00089	408.4	52.7	271.1	5.4	255.5	5.5	5.8	255.5	5.5
BS3 085	266	600	0.4	0.05638	0.00132	0.31754	0.00691	0.04086	0.00089	466.6	51.5	280.0	5.3	258.2	5.5	7.8	258.2	5.5
BS3 031	584	964	0.6	0.06778	0.00136	0.38362	0.00709	0.04106	0.00087	861.8	41.1	329.7	5.2	259.4	5.4	21.3	259.4	5.4
BS3 035	82	144	0.6	0.05379	0.00324	0.30531	0.01693	0.04118	0.00130	362.1	129.9	270.5	13.2	260.1	8.1	3.8	260.1	8.1
BS3 046	120	298	0.4	0.06174	0.00217	0.35057	0.01127	0.04119	0.00102	665.2	73.6	305.2	8.5	260.2	6.3	14.7	260.2	6.3
BS3 096	111	290	0.4	0.04800	0.00344	0.27508	0.01826	0.04157	0.00146	98.2	162.6	246.7	14.5	262.6	9.0	-6.4	262.6	9.0
BS3 098	107	223	0.5	0.05818	0.00325	0.33411	0.01704	0.04166	0.00131	536.0	118.4	292.7	13.0	263.1	8.1	10.1	263.1	8.1
BS3 077	349	708	0.5	0.05638	0.00218	0.32781	0.01164	0.04218	0.00109	466.7	84.4	287.9	8.9	266.3	6.7	7.5	266.3	6.7
~~BS3 055~~	~~304~~	~~608~~	~~0.5~~	~~0.07702~~	~~0.00156~~	~~0.44803~~	~~0.00827~~	~~0.04220~~	~~0.00090~~	~~1121.8~~	~~39.8~~	~~375.9~~	~~5.8~~	~~266.5~~	~~5.6~~	~~29.1~~	~~266.5~~	~~5.6~~
BS3 066	336	692	0.5	0.05582	0.00150	0.32476	0.00805	0.04221	0.00095	445.1	58.4	285.6	6.2	266.5	5.9	6.7	266.5	5.9
BS3 028	156	411	0.4	0.05822	0.00197	0.33944	0.01032	0.04231	0.00089	537.3	72.8	296.7	7.8	267.2	5.5	9.9	267.2	5.5
BS3 056	32	86	0.4	0.06201	0.00494	0.36219	0.02618	0.04238	0.00172	674.3	161.7	313.8	19.5	267.6	10.6	14.7	267.6	10.6
BS3 078	234	371	0.6	0.06038	0.00226	0.35278	0.01204	0.04239	0.00108	617.1	78.7	306.8	9.0	267.6	6.7	12.8	267.6	6.7
BS3 021	63	180	0.4	0.05147	0.00208	0.30262	0.01119	0.04267	0.00096	261.9	90.3	268.4	8.7	269.4	6.0	-0.4	269.4	6.0
BS3 038	305	750	0.4	0.05260	0.00199	0.31014	0.01080	0.04278	0.00107	311.5	83.8	274.3	8.4	270.0	6.6	1.6	270.0	6.6
BS3 044	133	437	0.3	0.05743	0.00178	0.33857	0.00966	0.04277	0.00101	507.5	67.4	296.1	7.3	270.0	6.2	8.8	270.0	6.2
BS3 072	272	581	0.5	0.05343	0.00146	0.31506	0.00798	0.04278	0.00096	346.9	60.6	278.1	6.2	270.1	5.9	2.9	270.1	5.9
BS3 090	202	409	0.5	0.05447	0.00168	0.32126	0.00919	0.04279	0.00100	390.6	67.6	282.9	7.1	270.1	6.2	4.5	270.1	6.2
BS3 027	79	308	0.3	0.06195	0.00339	0.36537	0.01803	0.04280	0.00125	672.3	113.1	316.2	13.4	270.2	7.8	14.5	270.2	7.8
BS3 015	299	624	0.5	0.05592	0.00202	0.33064	0.01099	0.04289	0.00107	448.8	78.4	290.1	8.4	270.7	6.6	6.7	270.7	6.6
BS3 043	351	839	0.4	0.05194	0.00190	0.30708	0.01035	0.04289	0.00106	282.8	81.4	271.9	8.0	270.7	6.5	0.4	270.7	6.5
BS3 054	306	623	0.5	0.05750	0.00138	0.34003	0.00754	0.04290	0.00094	510.5	52.2	297.2	5.7	270.8	5.8	8.9	270.8	5.8
BS3 040	376	1735	0.2	0.05149	0.00088	0.30476	0.00494	0.04294	0.00087	262.9	38.8	270.1	3.8	271.0	5.4	-0.3	271.0	5.4

BS3 071	313	659	0.5	0.05770	0.00159	0.34145	0.00869	0.04294	0.00098	518.0	59.7	298.3	6.6	271.0	6.0	9.2	271.0	6.0
BS3 080	100	175	0.6	0.06438	0.00263	0.38128	0.01416	0.04296	0.00115	754.3	84.0	328.0	10.4	271.2	7.1	17.3	271.2	7.1
BS3 036	253	499	0.5	0.05297	0.00151	0.31463	0.00833	0.04309	0.00097	327.4	63.6	277.8	6.4	272.0	6.0	2.1	272.0	6.0
BS3 022	73	175	0.4	0.06112	0.00381	0.36302	0.02057	0.04311	0.00136	643.3	128.8	314.5	15.3	272.1	8.4	13.5	272.1	8.4
BS3 057	198	382	0.5	0.05354	0.00178	0.31820	0.00974	0.04312	0.00103	351.8	73.3	280.5	7.5	272.1	6.4	3.0	272.1	6.4
BS3 079	243	427	0.6	0.05436	0.00138	0.32310	0.00761	0.04312	0.00095	385.9	55.7	284.3	5.8	272.1	5.9	4.3	272.1	5.9
BS3 025	265	527	0.5	0.06217	0.00146	0.36941	0.00769	0.04312	0.00078	679.9	49.3	319.2	5.7	272.2	4.8	14.7	272.2	4.8
BS3 060	131	344	0.4	0.06170	0.00181	0.36885	0.00993	0.04337	0.00101	663.9	61.8	318.8	7.4	273.7	6.2	14.1	273.7	6.2
BS3 081	133	211	0.6	0.05470	0.00228	0.32753	0.01256	0.04344	0.00114	400.1	90.2	287.7	9.6	274.1	7.1	4.7	274.1	7.1
BS3 063	177	474	0.4	0.05789	0.00186	0.34823	0.01029	0.04364	0.00104	525.4	69.1	303.4	7.8	275.4	6.4	9.2	275.4	6.4
BS3 033	47	104	0.4	0.05268	0.00306	0.31742	0.01695	0.04371	0.00136	315.1	126.7	279.9	13.1	275.8	8.4	1.5	275.8	8.4
BS3 006	118	142	0.8	0.05017	0.00484	0.30259	0.02703	0.04376	0.00183	202.8	209.7	268.4	21.1	276.1	11.3	-2.9	276.1	11.3
BS3 034	259	410	0.6	0.05908	0.00154	0.35659	0.00856	0.04379	0.00098	569.9	55.8	309.7	6.4	276.3	6.0	10.8	276.3	6.0
BS3 092	145	178	0.8	0.05163	0.00195	0.31412	0.01101	0.04413	0.00110	269.2	84.2	277.4	8.5	278.4	6.8	-0.4	278.4	6.8
BS3 061	587	519	1.1	0.05507	0.00151	0.33517	0.00851	0.04416	0.00100	415.0	59.6	293.5	6.5	278.5	6.2	5.1	278.5	6.2
BS3 069	223	240	0.9	0.05639	0.00217	0.34345	0.01215	0.04419	0.00113	466.9	83.8	299.8	9.2	278.7	7.0	7.0	278.7	7.0
BS3 095	172	285	0.6	0.05459	0.00165	0.33383	0.00935	0.04436	0.00103	395.6	65.7	292.5	7.1	279.8	6.4	4.3	279.8	6.4
BS3 026	282	555	0.5	0.05764	0.00179	0.35242	0.00989	0.04437	0.00089	516.0	67.2	306.5	7.4	279.9	5.5	8.7	279.9	5.5
BS3 050	88	108	0.8	0.05618	0.00286	0.34568	0.01613	0.04463	0.00131	458.8	109.7	301.5	12.2	281.5	8.1	6.6	281.5	8.1
BS3 001	118	144	0.8	0.05995	0.00518	0.37141	0.02917	0.04495	0.00188	601.7	176.9	320.7	21.6	283.5	11.6	11.6	283.5	11.6
BS3 059	157	182	0.9	0.05906	0.00291	0.36632	0.01645	0.04500	0.00131	569.5	103.8	316.9	12.2	283.7	8.1	10.5	283.7	8.1
BS3 003	262	267	1.0	0.05501	0.00246	0.34237	0.01390	0.04516	0.00110	412.6	96.5	299.0	10.5	284.7	6.8	4.8	284.7	6.8
BS3 032	309	323	1.0	0.05642	0.00158	0.35311	0.00915	0.04540	0.00102	468.4	61.2	307.1	6.9	286.2	6.3	6.8	286.2	6.3
BS3 049	666	526	1.3	0.06617	0.00319	0.41494	0.01806	0.04550	0.00135	811.7	97.8	352.4	13.0	286.8	8.3	18.6	286.8	8.3
BS3 068	230	231	1.0	0.05252	0.00194	0.33027	0.01129	0.04563	0.00113	307.9	82.0	289.8	8.6	287.6	7.0	0.8	287.6	7.0
BS3 062	146	238	0.6	0.05513	0.00285	0.34723	0.01641	0.04570	0.00135	417.3	111.1	302.6	12.4	288.0	8.4	4.8	288.0	8.4
BS3 053	125	163	0.8	0.06498	0.00263	0.41107	0.01511	0.04590	0.00122	773.7	82.9	349.6	10.9	289.3	7.5	17.2	289.3	7.5
BS3 010	115	157	0.7	0.05720	0.00326	0.36189	0.01871	0.04591	0.00135	498.7	121.2	313.6	14.0	289.4	8.3	7.7	289.4	8.3
BS3 004	70	102	0.7	0.05211	0.00321	0.33010	0.01869	0.04596	0.00137	290.3	134.8	289.6	14.3	289.7	8.5	0.0	289.7	8.5
BS3 002	126	157	0.8	0.04934	0.00223	0.31494	0.01308	0.04632	0.00110	163.9	102.5	278.0	10.1	291.9	6.8	-5.0	291.9	6.8
BS3 018	170	204	0.8	0.06033	0.00190	0.39182	0.01107	0.04713	0.00096	615.3	66.6	335.7	8.1	296.9	5.9	11.6	296.9	5.9

BS3 020	228	338	0.7	0.06818	0.00173	0.44419	0.00996	0.04728	0.00089	874.0	51.7	373.2	7.0	297.8	5.5	20.2	297.8	5.5
BS3 014	178	256	0.7	0.05727	0.00193	0.37334	0.01136	0.04730	0.00098	501.6	72.9	322.1	8.4	297.9	6.1	7.5	297.9	6.1
BS3 011	285	447	0.6	0.06494	0.00210	0.42546	0.01227	0.04754	0.00100	772.4	66.6	359.9	8.7	299.4	6.1	16.8	299.4	6.1
~~BS3 023~~	~~98~~	~~142~~	~~0.7~~	~~0.08883~~	~~0.00286~~	~~0.58290~~	~~0.01627~~	~~0.04762~~	~~0.00107~~	~~1400.5~~	~~60.4~~	~~466.3~~	~~10.4~~	~~299.9~~	~~6.6~~	~~35.7~~	~~299.9~~	~~6.6~~
BS3 009	71	103	0.7	0.05648	0.00376	0.37789	0.02293	0.04855	0.00160	470.6	141.7	325.5	16.9	305.6	9.9	6.1	305.6	9.9
BS3 024	159	167	0.9	0.05944	0.00507	0.39858	0.03087	0.04866	0.00201	583.4	175.0	340.6	22.4	306.3	12.4	10.1	306.3	12.4
BS3 012	112	144	0.8	0.06942	0.00297	0.46855	0.01790	0.04898	0.00123	911.4	85.7	390.2	12.4	308.2	7.6	21.0	308.2	7.6
~~BS3 029~~	~~135~~	~~204~~	~~0.7~~	~~0.09286~~	~~0.00348~~	~~0.64188~~	~~0.02078~~	~~0.05017~~	~~0.00125~~	~~1484.9~~	~~69.5~~	~~503.5~~	~~12.9~~	~~315.5~~	~~7.7~~	~~37.3~~	~~315.5~~	~~7.7~~
BS3 030	101	157	0.6	0.06123	0.00236	0.50419	0.01755	0.05976	0.00137	647.2	80.6	414.5	11.9	374.2	8.4	9.7	374.2	8.4
BS3 091	82	149	0.6	0.06239	0.00257	0.57152	0.02147	0.06645	0.00181	687.6	85.5	459.0	13.9	414.8	10.9	9.6	414.8	10.9
BS3 013	144	260	0.6	0.06957	0.00210	0.66857	0.01798	0.06974	0.00145	915.6	61.0	519.8	10.9	434.6	8.7	16.4	434.6	8.7
BS3 008	24	304	0.1	0.06034	0.00156	0.58071	0.01345	0.06984	0.00131	615.7	54.9	464.9	8.6	435.2	7.9	6.4	435.2	7.9
BS3 005	72	231	0.3	0.06326	0.00204	0.61045	0.01762	0.07002	0.00148	717.0	66.9	483.8	11.1	436.3	8.9	9.8	436.3	8.9
BS3 017	191	349	0.5	0.05831	0.00142	0.56488	0.01230	0.07030	0.00128	540.9	53.0	454.7	8.0	438.0	7.7	3.7	438.0	7.7
BS3 016	71	165	0.4	0.05981	0.00197	0.58093	0.01723	0.07049	0.00149	596.6	69.7	465.1	11.1	439.1	9.0	5.6	439.1	9.0
~~BS3 007~~	~~67~~	~~128~~	~~0.5~~	~~0.08794~~	~~0.00299~~	~~0.86610~~	~~0.02574~~	~~0.07146~~	~~0.00165~~	~~1381.2~~	~~63.8~~	~~633.4~~	~~14.0~~	~~445.0~~	~~10.0~~	~~29.7~~	~~445.0~~	~~10.0~~
BS3 019	140	303	0.5	0.06519	0.00150	0.66072	0.01346	0.07356	0.00134	780.4	47.5	515.1	8.2	457.5	8.0	11.2	457.5	8.0
BS3 073	62	127	0.5	0.06026	0.00223	0.61705	0.02100	0.07429	0.00190	612.9	78.0	488.0	13.2	462.0	11.4	5.3	462.0	11.4
BS3 037	116	166	0.7	0.05910	0.00180	0.61074	0.01720	0.07498	0.00175	570.7	65.0	484.0	10.8	466.1	10.5	3.7	466.1	10.5
BS3 064	56	123	0.5	0.06934	0.00199	1.13454	0.02996	0.11870	0.00283	909.0	58.0	769.9	14.3	723.1	16.3	6.1	723.1	16.3
BS3 052	64	181	0.4	0.06832	0.00157	1.15024	0.02448	0.12216	0.00270	878.1	46.8	777.4	11.6	743.0	15.5	4.4	743.0	15.5
BS3 097	168	538	0.3	0.07541	0.00097	1.81476	0.02285	0.17457	0.00354	1079.5	25.6	1050.8	8.2	1037.3	19.4	1.3	1079.5	25.6
BS3 074	571	790	0.7	0.09249	0.00103	2.77322	0.03078	0.21754	0.00433	1477.3	21.2	1348.4	8.3	1268.8	22.9	5.9	1477.3	21.2
QL1 082	121	205	0.6	0.05917	0.00327	0.31537	0.01586	0.03866	0.00102	573.4	115.7	278.3	12.2	244.5	6.3	12.1	244.5	6.3
QL1 081	95	152	0.6	0.05415	0.00236	0.31137	0.01256	0.04171	0.00085	377.1	94.5	275.2	9.7	263.4	5.3	4.3	263.4	5.3
QL1 015	157	226	0.7	0.05367	0.00156	0.31332	0.00848	0.04235	0.00066	357.1	64.4	276.7	6.6	267.4	4.1	3.4	267.4	4.1
QL1 035	179	219	0.8	0.05335	0.00154	0.31377	0.00838	0.04267	0.00068	343.5	64.1	277.1	6.5	269.3	4.2	2.8	269.3	4.2
QL1 072	177	194	0.9	0.05516	0.00190	0.32597	0.01034	0.04287	0.00077	418.5	74.7	286.5	7.9	270.6	4.7	5.5	270.6	4.7
QL1 009	138	271	0.5	0.05564	0.00280	0.32895	0.01518	0.04288	0.00102	437.9	108.5	288.8	11.6	270.7	6.3	6.3	270.7	6.3
QL1 091	108	179	0.6	0.05178	0.00198	0.30787	0.01093	0.04312	0.00078	275.5	85.2	272.5	8.5	272.1	4.8	0.1	272.1	4.8

QL1 028	88	223	0.4	0.05040	0.00146	0.30132	0.00813	0.04337	0.00067	213.3	65.7	267.4	6.3	273.7	4.2	-2.4	273.7	4.2
QL1 006	52	130	0.4	0.05626	0.00236	0.34363	0.01329	0.04431	0.00090	461.9	91.2	299.9	10.0	279.5	5.6	6.8	279.5	5.6
QL1 042	78	71	1.1	0.06362	0.00361	0.38974	0.02036	0.04444	0.00115	729.2	115.9	334.2	14.9	280.3	7.1	16.1	280.3	7.1
QL1 055	80	165	0.5	0.05559	0.00189	0.34351	0.01080	0.04483	0.00078	435.6	73.8	299.8	8.2	282.7	4.8	5.7	282.7	4.8
QL1 012	142	240	0.6	0.05983	0.00170	0.37493	0.00978	0.04546	0.00073	597.4	60.5	323.3	7.2	286.6	4.5	11.4	286.6	4.5
QL1 054	29	76	0.4	0.05538	0.00331	0.34918	0.01941	0.04573	0.00119	427.5	128.4	304.1	14.6	288.3	7.3	5.2	288.3	7.3
QL1 087	175	175	1.0	0.05575	0.00230	0.35407	0.01347	0.04606	0.00091	442.1	89.3	307.8	10.1	290.3	5.6	5.7	290.3	5.6
QL1 022	589	319	1.8	0.05543	0.00142	0.36164	0.00853	0.04733	0.00072	429.3	55.8	313.4	6.4	298.1	4.4	4.9	298.1	4.4
QL1 021	103	83	1.2	0.05566	0.00290	0.38775	0.01868	0.05054	0.00121	438.4	112.3	332.7	13.7	317.8	7.4	4.5	317.8	7.4
QL1 058	6	161	0.0	0.05562	0.00190	0.39198	0.01234	0.05113	0.00091	436.8	74.1	335.8	9.0	321.4	5.6	4.3	321.4	5.6
QL1 032	131	84	1.6	0.05351	0.00228	0.37895	0.01508	0.05137	0.00100	350.6	93.2	326.3	11.1	322.9	6.1	1.0	322.9	6.1
QL1 073	296	409	0.7	0.05715	0.00111	0.46073	0.00830	0.05848	0.00079	496.8	42.3	384.8	5.8	366.4	4.8	4.8	366.4	4.8
QL1 024	96	72	1.3	0.05560	0.00313	0.46645	0.02446	0.06086	0.00151	436.0	121.0	388.7	16.9	380.9	9.2	2.0	380.9	9.2
QL1 051	11	23	0.5	0.05560	0.00482	0.51089	0.04211	0.06666	0.00211	436.0	182.7	419.0	28.3	416.0	12.7	0.7	416.0	12.7
QL1 016	35	175	0.2	0.05591	0.00157	0.51559	0.01337	0.06689	0.00107	448.4	61.2	422.2	9.0	417.4	6.5	1.1	417.4	6.5
QL1 017	62	249	0.2	0.05388	0.00116	0.50604	0.01009	0.06812	0.00094	366.0	47.8	415.8	6.8	424.8	5.7	-2.2	424.8	5.7
QL1 095	96	306	0.3	0.05355	0.00126	0.50513	0.01095	0.06840	0.00098	352.1	52.4	415.2	7.4	426.5	5.9	-2.7	426.5	5.9
QL1 090	69	137	0.5	0.05223	0.00185	0.49606	0.01618	0.06887	0.00122	295.4	78.6	409.0	11.0	429.4	7.3	-5.0	429.4	7.3
QL1 083	17	54	0.3	0.05701	0.00334	0.54394	0.02948	0.06920	0.00184	491.4	124.7	441.0	19.4	431.4	11.1	2.2	431.4	11.1
QL1 064	46	48	0.9	0.05939	0.00282	0.58495	0.02571	0.07144	0.00162	581.5	100.0	467.6	16.5	444.8	9.7	4.9	444.8	9.7
QL1 010	166	193	0.9	0.05538	0.00129	0.54882	0.01183	0.07188	0.00104	427.4	50.7	444.2	7.8	447.5	6.3	-0.7	447.5	6.3
QL1 060	194	270	0.7	0.05628	0.00113	0.55831	0.01043	0.07196	0.00098	462.6	44.3	450.4	6.8	448.0	5.9	0.5	448.0	5.9
QL1 096	40	244	0.2	0.05832	0.00154	0.58222	0.01406	0.07240	0.00112	541.0	57.4	465.9	9.0	450.6	6.7	3.3	450.6	6.7
QL1 075	155	125	1.2	0.05923	0.00268	0.60681	0.02522	0.07431	0.00167	575.7	95.3	481.5	15.9	462.1	10.0	4.0	462.1	10.0
QL1 001	172	422	0.4	0.05825	0.00091	0.59987	0.00874	0.07469	0.00093	538.7	34.6	477.2	5.5	464.4	5.6	2.7	464.4	5.6
QL1 062	167	229	0.7	0.05948	0.00120	0.61701	0.01155	0.07525	0.00104	584.6	43.4	488.0	7.3	467.7	6.2	4.2	467.7	6.2
QL1 086	102	128	0.8	0.05689	0.00193	0.59632	0.01852	0.07601	0.00134	486.8	73.6	474.9	11.8	472.2	8.0	0.6	472.2	8.0
QL1 038	172	309	0.6	0.05740	0.00098	0.60546	0.00962	0.07652	0.00098	506.4	37.6	480.7	6.1	475.3	5.9	1.1	475.3	5.9
QL1 098	50	73	0.7	0.05578	0.00249	0.59149	0.02425	0.07689	0.00162	443.4	95.9	471.8	15.5	477.6	9.7	-1.2	477.6	9.7
QL1 019	296	607	0.5	0.05789	0.00083	0.61973	0.00829	0.07765	0.00095	525.4	31.5	489.7	5.2	482.1	5.7	1.6	482.1	5.7
QL1 023	28	244	0.1	0.05895	0.00122	0.67564	0.01290	0.08315	0.00116	565.1	44.4	524.1	7.8	514.9	6.9	1.8	514.9	6.9

QL1 067	765	1034	0.7	0.06663	0.00062	1.14622	0.01027	0.12478	0.00141	826.4	19.4	775.5	4.9	758.0	8.1	2.3	758.0	8.1
QL1 030	29	246	0.1	0.09320	0.00120	1.80867	0.02135	0.14077	0.00177	1492.0	24.3	1048.6	7.7	849.0	10.0	19.0	849.0	10.0
QL1 037	99	454	0.2	0.06897	0.00073	1.38001	0.01386	0.14515	0.00168	897.8	21.8	880.4	5.9	873.7	9.5	0.8	873.7	9.5
QL1 056	55	208	0.3	0.09665	0.00159	1.98415	0.02953	0.14892	0.00208	1560.4	30.6	1110.1	10.1	894.9	11.7	19.4	894.9	11.7
QL1 036	255	618	0.4	0.08519	0.00067	2.60609	0.01989	0.22190	0.00247	1319.9	15.2	1302.4	5.6	1291.9	13.0	2.1	1319.9	15.2
QL1 029	19	51	0.4	0.08826	0.00228	2.72506	0.06446	0.22396	0.00403	1388.2	48.7	1335.3	17.6	1302.8	21.2	6.2	1388.2	48.7
QL1 041	29	26	1.1	0.09807	0.00367	2.77860	0.09429	0.20552	0.00489	1587.8	68.3	1349.8	25.3	1204.9	26.1	24.1	1587.8	68.3
~~QL1 080~~	~~18~~	~~65~~	~~0.3~~	~~0.09813~~	~~0.00220~~	~~2.56938~~	~~0.05211~~	~~0.18993~~	~~0.00316~~	~~1588.9~~	~~41.3~~	~~1292.0~~	~~14.8~~	~~1121.0~~	~~17.1~~	~~29.4~~	~~1588.9~~	~~41.3~~
QL1 014	18	24	0.7	0.10131	0.00253	4.11909	0.09512	0.29493	0.00545	1648.3	45.7	1658.1	18.9	1666.1	27.1	-1.1	1648.3	45.7
QL1 088	45	54	0.8	0.10234	0.00194	4.28503	0.07154	0.30363	0.00455	1667.0	34.7	1690.5	13.7	1709.3	22.5	-2.5	1667.0	34.7
QL1 034	78	87	0.9	0.10310	0.00143	4.15108	0.05377	0.29206	0.00392	1680.7	25.4	1664.4	10.6	1651.8	19.5	1.7	1680.7	25.4
QL1 089	125	110	1.1	0.10313	0.00148	4.18648	0.05347	0.29439	0.00389	1681.1	26.3	1671.4	10.5	1663.4	19.4	1.1	1681.1	26.3
QL1 027	58	104	0.6	0.10506	0.00130	4.27599	0.04942	0.29525	0.00378	1715.3	22.5	1688.8	9.5	1667.7	18.8	2.8	1715.3	22.5
QL1 094	160	108	1.5	0.10544	0.00158	4.09614	0.05420	0.28173	0.00378	1721.9	27.3	1653.5	10.8	1600.1	19.0	7.1	1721.9	27.3
QL1 003	42	50	0.8	0.10596	0.00182	4.50148	0.07165	0.30817	0.00460	1731.0	31.2	1731.2	13.2	1731.7	22.7	0.0	1731.0	31.2
QL1 050	9	15	0.6	0.10648	0.00632	3.90659	0.21150	0.26615	0.01004	1739.9	105.1	1615.0	43.8	1521.2	51.1	12.6	1739.9	####
QL1 026	43	70	0.6	0.10809	0.00151	4.79121	0.06252	0.32153	0.00437	1767.5	25.3	1783.4	11.0	1797.2	21.3	-1.7	1767.5	25.3
QL1 053	213	344	0.6	0.10813	0.00090	4.47293	0.03602	0.30006	0.00343	1768.1	15.2	1726.0	6.7	1691.6	17.0	4.3	1768.1	15.2
QL1 099	56	65	0.9	0.10860	0.00203	4.50840	0.07379	0.30104	0.00450	1776.1	33.7	1732.5	13.6	1696.5	22.3	4.5	1776.1	33.7
QL1 025	34	54	0.6	0.10868	0.00247	3.93810	0.08143	0.26285	0.00464	1777.4	41.0	1621.5	16.7	1504.4	23.7	15.4	1777.4	41.0
QL1 061	28	52	0.5	0.10914	0.00188	4.89984	0.07855	0.32565	0.00492	1785.1	31.1	1802.2	13.5	1817.3	24.0	-1.8	1785.1	31.1
QL1 020	57	109	0.5	0.10960	0.00129	4.56055	0.05014	0.30183	0.00381	1792.8	21.2	1742.1	9.2	1700.4	18.9	5.2	1792.8	21.2
QL1 047	109	219	0.5	0.11082	0.00106	4.35969	0.03961	0.28539	0.00338	1812.8	17.3	1704.7	7.5	1618.4	17.0	10.7	1812.8	17.3
QL1 039	81	52	1.6	0.11095	0.00184	4.93087	0.07579	0.32239	0.00479	1815.0	29.8	1807.6	13.0	1801.4	23.3	0.7	1815.0	29.8
QL1 004	37	37	1.0	0.11128	0.00222	4.91484	0.09078	0.32037	0.00529	1820.4	35.8	1804.8	15.6	1791.6	25.8	1.6	1820.4	35.8
QL1 059	8	22	0.4	0.11166	0.00283	4.89132	0.11427	0.31776	0.00610	1826.6	45.2	1800.8	19.7	1778.8	29.9	2.6	1826.6	45.2
QL1 044	49	79	0.6	0.11235	0.00152	5.16260	0.06536	0.33333	0.00451	1837.7	24.3	1846.5	10.8	1854.5	21.8	-0.9	1837.7	24.3
QL1 011	140	118	1.2	0.11273	0.00125	5.07434	0.05296	0.32654	0.00406	1843.8	19.9	1831.8	8.9	1821.6	19.8	1.2	1843.8	19.9
QL1 076	69	953	0.1	0.11285	0.00078	4.72935	0.03244	0.30399	0.00336	1845.8	12.5	1772.5	5.8	1711.0	16.6	7.3	1845.8	12.5
QL1 045	99	655	0.2	0.11291	0.00077	5.03822	0.03430	0.32369	0.00357	1846.8	12.4	1825.8	5.8	1807.7	17.4	2.1	1846.8	12.4
QL1 071	86	166	0.5	0.11296	0.00113	5.17948	0.04937	0.33261	0.00402	1847.6	18.0	1849.3	8.1	1851.0	19.5	-0.2	1847.6	18.0

QL1 100	32	209	0.2	0.11301	0.00126	4.70433	0.04696	0.30188	0.00368	1848.4	20.0	1768.0	8.4	1700.6	18.2	8.0	1848.4	20.0
QL1 070	60	95	0.6	0.11457	0.00145	5.19773	0.06169	0.32908	0.00434	1873.2	22.7	1852.2	10.1	1833.9	21.0	2.1	1873.2	22.7
QL1 018	80	97	0.8	0.11509	0.00137	5.19261	0.05810	0.32728	0.00420	1881.3	21.3	1851.4	9.5	1825.2	20.4	3.0	1881.3	21.3
QL1 002	32	77	0.4	0.11529	0.00155	5.15127	0.06448	0.32410	0.00437	1884.4	24.0	1844.6	10.6	1809.7	21.3	4.0	1884.4	24.0
QL1 046	96	259	0.4	0.11637	0.00100	5.33740	0.04440	0.33271	0.00386	1901.2	15.4	1874.9	7.1	1851.5	18.7	2.6	1901.2	15.4
QL1 013	14	51	0.3	0.11690	0.00181	5.60769	0.08089	0.34797	0.00506	1909.4	27.5	1917.3	12.4	1924.9	24.2	-0.8	1909.4	27.5
QL1 031	11	20	0.5	0.11698	0.00272	5.64817	0.12191	0.35025	0.00649	1910.6	41.2	1923.5	18.6	1935.8	31.0	-1.3	1910.6	41.2
QL1 063	45	309	0.1	0.11748	0.00099	5.61109	0.04591	0.34648	0.00401	1918.2	15.1	1917.8	7.1	1917.7	19.2	0.0	1918.2	15.1
QL1 007	43	93	0.5	0.12011	0.00140	5.80267	0.06393	0.35045	0.00449	1957.8	20.7	1946.8	9.5	1936.8	21.5	1.1	1957.8	20.7
QL1 078	66	61	1.1	0.12139	0.00180	5.80843	0.08026	0.34709	0.00496	1976.8	26.2	1947.7	12.0	1920.7	23.7	2.8	1976.8	26.2
QL1 084	243	147	1.7	0.12286	0.00144	5.97409	0.06615	0.35270	0.00456	1998.2	20.7	1972.1	9.6	1947.5	21.7	2.5	1998.2	20.7
QL1 048	66	167	0.4	0.12360	0.00115	6.12361	0.05478	0.35939	0.00429	2008.8	16.5	1993.6	7.8	1979.3	20.4	1.5	2008.8	16.5
QL1 008	80	189	0.4	0.12412	0.00110	6.29422	0.05372	0.36786	0.00432	2016.2	15.7	2017.6	7.5	2019.3	20.4	-0.2	2016.2	15.7
QL1 069	45	234	0.2	0.12437	0.00106	6.12054	0.05040	0.35697	0.00416	2019.9	15.0	1993.2	7.2	1967.8	19.8	2.6	2019.9	15.0
QL1 077	187	172	1.1	0.12448	0.00143	5.97202	0.06488	0.34799	0.00447	2021.4	20.3	1971.8	9.5	1925.0	21.4	4.8	2021.4	20.3
~~QL1 097~~	~~31~~	~~71~~	~~0.4~~	~~0.12496~~	~~0.00223~~	~~3.93586~~	~~0.06048~~	~~0.22840~~	~~0.00338~~	~~2028.3~~	~~31.3~~	~~1621.1~~	~~12.4~~	~~1326.1~~	~~17.8~~	~~34.6~~	~~2028.3~~	~~31.3~~
QL1 065	16	19	0.8	0.12854	0.00528	6.50715	0.24582	0.36722	0.01120	2078.1	70.6	2046.9	33.3	2016.3	52.8	3.0	2078.1	70.6
QL1 043	56	56	1.0	0.13120	0.00187	7.01820	0.09381	0.38805	0.00555	2114.0	24.8	2113.7	11.9	2113.8	25.8	0.0	2114.0	24.8
QL1 066	41	52	0.8	0.13864	0.00231	5.83100	0.08861	0.30509	0.00470	2210.3	28.6	1951.0	13.2	1716.5	23.2	22.3	2210.3	28.6
QL1 074	232	335	0.7	0.14794	0.00154	8.03608	0.07958	0.39403	0.00498	2322.2	17.8	2235.1	8.9	2141.5	23.1	7.8	2322.2	17.8
QL1 092	103	192	0.5	0.15162	0.00131	9.14491	0.07221	0.43738	0.00511	2364.3	14.7	2352.6	7.2	2338.9	22.9	1.1	2364.3	14.7
QL1 049	39	66	0.6	0.15326	0.00175	9.56101	0.10458	0.45254	0.00602	2382.6	19.4	2393.4	10.1	2406.5	26.7	-1.0	2382.6	19.4
QL1 093	91	122	0.7	0.15486	0.00159	9.15440	0.08340	0.42867	0.00526	2400.3	17.4	2353.6	8.3	2299.7	23.7	4.2	2400.3	17.4
QL1 057	43	159	0.3	0.15636	0.00132	9.50568	0.07825	0.44099	0.00523	2416.7	14.3	2388.1	7.6	2355.1	23.4	2.5	2416.7	14.3
QL1 040	69	123	0.6	0.15702	0.00281	10.14780	0.17132	0.46881	0.00812	2423.8	30.0	2448.3	15.6	2478.3	35.7	-2.2	2423.8	30.0
QL1 085	70	81	0.9	0.15793	0.00183	9.91105	0.10053	0.45509	0.00584	2433.7	19.5	2426.5	9.4	2417.8	25.9	0.7	2433.7	19.5
QL1 068	80	94	0.8	0.16311	0.00165	10.39323	0.10123	0.46221	0.00588	2488.2	16.9	2470.4	9.0	2449.3	25.9	1.6	2488.2	16.9
QL1 033	224	113	2.0	0.16321	0.00160	10.53585	0.09977	0.46829	0.00589	2489.2	16.4	2483.1	8.8	2476.0	25.9	0.5	2489.2	16.4
QL1 052	176	276	0.6	0.16368	0.00119	9.52067	0.06855	0.42195	0.00480	2494.0	12.2	2389.5	6.6	2269.3	21.8	9.0	2494.0	12.2
QL1 005	245	234	1.0	0.16674	0.00126	10.98149	0.08162	0.47774	0.00549	2525.2	12.6	2521.6	6.9	2517.4	24.0	0.3	2525.2	12.6
QL1 079	123	273	0.5	0.16843	0.00123	11.34345	0.08268	0.48853	0.00559	2542.1	12.2	2551.8	6.8	2564.3	24.2	-0.9	2542.1	12.2

~~QL2 066~~	~~465~~	~~873~~	~~0.5~~	~~0.07664~~	~~0.00185~~	~~0.41563~~	~~0.00960~~	~~0.03933~~	~~0.00088~~	~~1111.9~~	~~47.4~~	~~352.9~~	~~6.9~~	~~248.7~~	~~5.5~~	~~-29.5~~	~~248.7~~	~~5.5~~
QL2 083	180	343	0.5	0.05777	0.00191	0.33108	0.01052	0.04157	0.00097	520.8	71.1	290.4	8.0	262.5	6.0	-9.6	262.5	6.0
QL2 024	91	271	0.3	0.05882	0.00249	0.34130	0.01378	0.04207	0.00103	560.4	89.6	298.2	10.4	265.7	6.4	-10.9	265.7	6.4
QL2 038	176	219	0.8	0.05754	0.00251	0.34194	0.01424	0.04309	0.00107	511.9	93.3	298.6	10.8	272.0	6.6	-8.9	272.0	6.6
QL2 065	566	410	1.4	0.06129	0.00145	0.37253	0.00856	0.04408	0.00097	649.3	50.0	321.5	6.3	278.1	6.0	-13.5	278.1	6.0
~~QL2 052~~	~~1598~~	~~1304~~	~~1.2~~	~~0.09868~~	~~0.00184~~	~~0.61673~~	~~0.01109~~	~~0.04532~~	~~0.00098~~	~~1599.4~~	~~34.5~~	~~487.8~~	~~7.0~~	~~285.7~~	~~6.1~~	~~-41.4~~	~~285.7~~	~~6.1~~
~~QL2 050~~	~~261~~	~~285~~	~~0.9~~	~~0.07419~~	~~0.00192~~	~~0.53417~~	~~0.01325~~	~~0.05221~~	~~0.00117~~	~~1046.7~~	~~51.4~~	~~434.6~~	~~8.8~~	~~328.1~~	~~7.2~~	~~-24.5~~	~~328.1~~	~~7.2~~
QL2 032	67	333	0.2	0.05798	0.00172	0.49285	0.01411	0.06164	0.00139	528.5	64.6	406.8	9.6	385.6	8.4	-5.2	385.6	8.4
~~QL2 051~~	~~698~~	~~990~~	~~0.7~~	~~0.10073~~	~~0.00139~~	~~0.92219~~	~~0.01271~~	~~0.06638~~	~~0.00140~~	~~1637.7~~	~~25.4~~	~~663.5~~	~~6.7~~	~~414.3~~	~~8.5~~	~~-37.6~~	~~414.3~~	~~8.5~~
QL2 030	284	387	0.7	0.06556	0.00220	0.62024	0.01991	0.06860	0.00161	792.3	69.0	490.0	12.5	427.7	9.7	-12.7	427.7	9.7
QL2 084	679	1068	0.6	0.06079	0.00080	0.58183	0.00789	0.06942	0.00147	631.7	28.2	465.6	5.1	432.7	8.9	-7.1	432.7	8.9
QL2 096	288	384	0.7	0.05936	0.00093	0.58652	0.00929	0.07167	0.00154	580.4	33.8	468.6	6.0	446.2	9.3	-4.8	446.2	9.3
QL2 075	163	290	0.6	0.05822	0.00159	0.57626	0.01522	0.07179	0.00162	537.3	59.2	462.1	9.8	446.9	9.7	-3.3	446.9	9.7
QL2 095	135	273	0.5	0.05781	0.00101	0.57400	0.01001	0.07203	0.00156	522.3	38.1	460.6	6.5	448.4	9.4	-2.6	448.4	9.4
QL2 015	204	363	0.6	0.05847	0.00106	0.58619	0.01020	0.07268	0.00159	547.3	39.2	468.4	6.5	452.3	9.5	-3.4	452.3	9.5
QL2 060	193	359	0.5	0.07277	0.00156	0.73894	0.01536	0.07363	0.00161	1007.6	42.9	561.8	9.0	458.0	9.7	-18.5	458.0	9.7
QL2 011	278	418	0.7	0.05908	0.00138	0.60123	0.01313	0.07378	0.00168	570.1	49.9	478.0	8.3	458.9	10.1	-4.0	458.9	10.1
QL2 100	595	1111	0.5	0.06124	0.00087	0.63411	0.00914	0.07512	0.00161	647.7	30.1	498.7	5.7	466.9	9.7	-6.4	466.9	9.7
QL2 048	548	827	0.7	0.07289	0.00099	0.75564	0.01037	0.07517	0.00157	1010.9	27.3	571.5	6.0	467.2	9.4	-18.3	467.2	9.4
QL2 078	206	390	0.5	0.06425	0.00165	0.68178	0.01688	0.07696	0.00173	749.9	53.2	527.9	10.2	477.9	10.4	-9.5	477.9	10.4
QL2 029	331	279	1.2	0.06324	0.00158	0.67128	0.01617	0.07697	0.00169	716.2	52.1	521.5	9.8	478.0	10.1	-8.3	478.0	10.1
QL2 092	228	395	0.6	0.06144	0.00178	0.65693	0.01839	0.07756	0.00179	654.6	61.1	512.7	11.3	481.6	10.7	-6.1	481.6	10.7
QL2 080	218	281	0.8	0.05935	0.00164	0.63811	0.01703	0.07798	0.00177	579.8	58.8	501.1	10.6	484.1	10.6	-3.4	484.1	10.6
QL2 094	218	331	0.7	0.05970	0.00163	0.64265	0.01700	0.07809	0.00178	593.1	57.7	503.9	10.5	484.7	10.7	-3.8	484.7	10.7
QL2 085	284	543	0.5	0.05890	0.00109	0.63856	0.01174	0.07863	0.00170	563.5	39.9	501.4	7.3	487.9	10.2	-2.7	487.9	10.2
QL2 093	295	274	1.1	0.06006	0.00118	0.65530	0.01266	0.07914	0.00173	605.9	41.9	511.7	7.8	491.0	10.4	-4.0	491.0	10.4
QL2 055	76	166	0.5	0.06754	0.00259	0.74864	0.02739	0.08038	0.00198	854.4	77.8	567.4	15.9	498.4	11.8	-12.2	498.4	11.8
~~QL2 088~~	~~1420~~	~~1233~~	~~1.2~~	~~0.10407~~	~~0.00115~~	~~1.20613~~	~~0.01399~~	~~0.08407~~	~~0.00178~~	~~1697.9~~	~~20.1~~	~~803.4~~	~~6.4~~	~~520.3~~	~~10.6~~	~~-35.2~~	~~520.3~~	~~10.6~~
QL2 059	112	207	0.5	0.06693	0.00136	1.07339	0.02138	0.11629	0.00253	835.7	41.9	740.4	10.5	709.2	14.6	-4.2	709.2	14.6
QL2 043	106	276	0.4	0.06735	0.00111	1.10149	0.01796	0.11859	0.00251	848.5	33.8	754.1	8.7	722.5	14.5	-4.2	722.5	14.5

QL2 062	65	168	0.4	0.07982	0.00174	1.31571	0.02775	0.11953	0.00265	1192.6	42.4	852.6	12.2	727.9	15.2	-14.6	727.9	15.2
QL2 045	165	545	0.3	0.07399	0.00135	1.23373	0.02210	0.12090	0.00259	1041.3	36.4	816.0	10.1	735.7	14.9	-9.8	735.7	14.9
QL2 058	144	333	0.4	0.06964	0.00114	1.16133	0.01890	0.12093	0.00257	917.7	33.3	782.6	8.9	735.9	14.8	-6.0	735.9	14.8
QL2 049	93	179	0.5	0.07326	0.00200	1.23056	0.03230	0.12179	0.00278	1021.2	54.4	814.6	14.7	740.9	16.0	-9.0	740.9	16.0
QL2 070	90	194	0.5	0.06601	0.00128	1.11410	0.02118	0.12240	0.00266	806.6	39.9	760.1	10.2	744.4	15.3	-2.1	744.4	15.3
QL2 053	80	223	0.4	0.06655	0.00133	1.12501	0.02207	0.12259	0.00265	823.6	41.3	765.4	10.6	745.4	15.2	-2.6	745.4	15.2
QL2 006	102	218	0.5	0.08406	0.00158	1.42083	0.02496	0.12257	0.00270	1293.9	36.2	897.7	10.5	745.4	15.5	-17.0	745.4	15.5
QL2 057	110	208	0.5	0.06943	0.00135	1.17381	0.02242	0.12260	0.00265	911.5	39.6	788.4	10.5	745.5	15.2	-5.4	745.5	15.2
QL2 087	85	177	0.5	0.06761	0.00115	1.14431	0.01940	0.12277	0.00265	856.6	35.0	774.6	9.2	746.5	15.2	-3.6	746.5	15.2
QL2 044	86	76	1.1	0.07445	0.00194	1.26411	0.03166	0.12312	0.00277	1053.3	51.9	829.8	14.2	748.5	15.9	-9.8	748.5	15.9
QL2 010	55	135	0.4	0.07498	0.00194	1.28588	0.03070	0.12435	0.00298	1068.0	51.2	839.5	13.6	755.6	17.1	-10.0	755.6	17.1
QL2 074	70	172	0.4	0.07706	0.00226	1.32439	0.03716	0.12465	0.00293	1122.6	57.5	856.4	16.2	757.3	16.8	-11.6	757.3	16.8
QL2 025	71	157	0.4	0.07575	0.00171	1.30376	0.02835	0.12480	0.00273	1088.4	44.5	847.4	12.5	758.2	15.7	-10.5	758.2	15.7
QL2 019	133	492	0.3	0.06891	0.00174	1.19485	0.02817	0.12568	0.00302	896.2	51.4	798.2	13.0	763.2	17.3	-4.4	763.2	17.3
QL2 003	144	327	0.4	0.06878	0.00117	1.19302	0.01930	0.12580	0.00268	892.2	34.6	797.4	8.9	763.9	15.4	-4.2	763.9	15.4
QL2 005	64	135	0.5	0.06885	0.00175	1.19493	0.02826	0.12586	0.00294	894.3	51.6	798.2	13.1	764.2	16.8	-4.3	764.2	16.8
QL2 086	53	129	0.4	0.07175	0.00170	1.25264	0.02868	0.12664	0.00285	978.7	47.4	824.6	12.9	768.7	16.3	-6.8	768.7	16.3
QL2 082	81	168	0.5	0.07183	0.00134	1.26391	0.02319	0.12762	0.00278	981.2	37.5	829.7	10.4	774.2	15.9	-6.7	774.2	15.9
QL2 030	76	200	0.4	0.07887	0.00180	1.38963	0.03058	0.12776	0.00281	1168.8	44.6	884.5	13.0	775.1	16.1	-12.4	775.1	16.1
QL2 056	66	177	0.4	0.07297	0.00186	1.29975	0.03186	0.12916	0.00291	1013.1	50.7	845.6	14.1	783.1	16.6	-7.4	783.1	16.6
QL2 069	104	341	0.3	0.06867	0.00159	1.22669	0.02755	0.12954	0.00288	889.0	47.1	812.8	12.6	785.2	16.4	-3.4	785.2	16.4
QL2 061	47	150	0.3	0.07510	0.00246	1.37301	0.04281	0.13258	0.00318	1071.2	64.3	877.5	18.3	802.5	18.1	-8.5	802.5	18.1
QL2 036	62	354	0.2	0.07363	0.00118	1.35025	0.02151	0.13297	0.00281	1031.3	32.1	867.7	9.3	804.8	16.0	-7.2	804.8	16.0
QL2 009	24	58	0.4	0.07021	0.00230	1.28874	0.03879	0.13311	0.00344	934.4	65.7	840.7	17.2	805.6	19.6	-4.2	805.6	19.6
QL2 031	135	332	0.4	0.06908	0.00107	1.27416	0.01970	0.13375	0.00281	901.1	31.7	834.3	8.8	809.2	16.0	-3.0	809.2	16.0
QL2 004	28	64	0.4	0.07123	0.00214	1.31370	0.03645	0.13376	0.00333	964.0	60.3	851.8	16.0	809.3	18.9	-5.0	809.3	18.9
QL2 017	88	271	0.3	0.06954	0.00108	1.28912	0.01944	0.13438	0.00291	914.9	31.5	840.9	8.6	812.8	16.5	-3.3	812.8	16.5
QL2 020	79	205	0.4	0.07035	0.00164	1.32394	0.02890	0.13642	0.00321	938.6	47.0	856.2	12.6	824.4	18.2	-3.7	824.4	18.2
QL2 047	729	800	0.9	0.07803	0.00087	1.94424	0.02259	0.18067	0.00375	1147.6	21.9	1096.5	7.8	1070.7	20.5	-2.4	1147.6	21.9
QL2 021	35	90	0.4	0.07968	0.00190	2.10045	0.04820	0.19114	0.00424	1189.2	46.3	1149.0	15.8	1127.5	23.0	-1.9	1189.2	46.3
QL2 001	147	297	0.5	0.08154	0.00109	2.29537	0.03003	0.20420	0.00423	1234.5	25.9	1210.9	9.3	1197.8	22.7	-1.1	1234.5	25.9

QL2 014	111	340	0.3	0.08197	0.00107	2.37265	0.03086	0.20985	0.00446	1244.7	25.3	1234.4	9.3	1228.0	23.8	-0.5	1244.7	25.3
QL2 037	108	216	0.5	0.08505	0.00117	2.47056	0.03427	0.21064	0.00441	1316.5	26.5	1263.5	10.0	1232.2	23.5	-2.5	1316.5	26.5
QL2 081	98	310	0.3	0.08970	0.00130	2.95786	0.04318	0.23917	0.00514	1419.0	27.4	1396.9	11.1	1382.4	26.7	-1.0	1419.0	27.4
QL2 018	222	946	0.2	0.09071	0.00099	3.00127	0.03401	0.23985	0.00504	1440.4	20.6	1407.9	8.6	1385.9	26.2	-1.6	1440.4	20.6
QL2 008	61	88	0.7	0.09260	0.00163	3.15603	0.05308	0.24715	0.00548	1479.6	33.2	1446.5	13.0	1423.8	28.4	-1.6	1479.6	33.2
QL2 034	85	166	0.5	0.09268	0.00135	3.00674	0.04375	0.23523	0.00496	1481.3	27.5	1409.3	11.1	1361.8	25.9	-3.4	1481.3	27.5
QL2 097	74	179	0.4	0.09414	0.00119	3.24488	0.04236	0.25004	0.00536	1511.0	23.7	1468.0	10.1	1438.7	27.6	-2.0	1511.0	23.7
QL2 063	81	184	0.4	0.09627	0.00132	3.15588	0.04377	0.23773	0.00505	1552.9	25.5	1446.4	10.7	1374.9	26.3	-4.9	1552.9	25.5
QL2 079	161	241	0.7	0.09737	0.00118	3.52430	0.04419	0.26251	0.00556	1574.3	22.5	1532.7	9.9	1502.7	28.4	-2.0	1574.3	22.5
QL2 042	96	148	0.6	0.09852	0.00234	2.57759	0.05842	0.18971	0.00431	1596.3	43.7	1294.3	16.6	1119.8	23.3	-13.5	1596.3	43.7
QL2 013	45	107	0.4	0.09874	0.00157	3.43063	0.05281	0.25189	0.00556	1600.4	29.4	1511.4	12.1	1448.2	28.6	-4.2	1600.4	29.4
QL2 068	41	82	0.5	0.09884	0.00180	3.14442	0.05598	0.23071	0.00506	1602.3	33.6	1443.6	13.7	1338.2	26.5	-7.3	1602.3	33.6
QL2 046	145	76	1.9	0.10093	0.00344	2.87198	0.09213	0.20633	0.00522	1641.3	61.9	1374.6	24.2	1209.2	27.9	-12.0	1641.3	61.9
QL2 091	75	174	0.4	0.10708	0.00125	4.39553	0.05383	0.29776	0.00635	1750.3	21.2	1711.5	10.1	1680.2	31.5	-1.8	1750.3	21.2
QL2 064	72	174	0.4	0.10727	0.00209	3.73017	0.07039	0.25216	0.00561	1753.6	35.2	1577.9	15.1	1449.6	28.9	-8.1	1753.6	35.2
QL2 089	239	286	0.8	0.10730	0.00119	4.33987	0.05092	0.29338	0.00622	1754.0	20.1	1701.0	9.7	1658.4	31.0	-2.5	1754.0	20.1
QL2 002	145	275	0.5	0.10781	0.00127	4.58323	0.05429	0.30836	0.00635	1762.7	21.4	1746.2	9.9	1732.6	31.3	-0.8	1762.7	21.4
QL2 012	71	90	0.8	0.10827	0.00171	4.60065	0.07051	0.30807	0.00684	1770.4	28.6	1749.4	12.8	1731.2	33.7	-1.0	1770.4	28.6
QL2 071	91	255	0.4	0.10934	0.00178	3.85103	0.06189	0.25542	0.00556	1788.4	29.4	1603.5	13.0	1466.4	28.6	-8.6	1788.4	29.4
QL2 067	145	146	1.0	0.10997	0.00269	3.91518	0.09137	0.25818	0.00604	1798.9	43.9	1616.8	18.9	1480.5	31.0	-8.4	1798.9	43.9
QL2 077	64	182	0.4	0.11165	0.00137	4.76067	0.06042	0.30925	0.00657	1826.4	22.2	1778.0	10.7	1737.0	32.4	-2.3	1826.4	22.2
QL2 023	52	100	0.5	0.11191	0.00159	4.85226	0.06875	0.31441	0.00662	1830.6	25.5	1794.0	11.9	1762.4	32.5	-1.8	1830.6	25.5
QL2 041	292	615	0.5	0.11218	0.00116	4.57859	0.05022	0.29594	0.00612	1835.1	18.7	1745.4	9.1	1671.1	30.4	-4.3	1835.1	18.7
QL2 022	51	72	0.7	0.11234	0.00172	4.80306	0.07272	0.31001	0.00658	1837.6	27.5	1785.4	12.7	1740.8	32.4	-2.5	1837.6	27.5
QL2 099	243	307	0.8	0.11364	0.00122	4.93160	0.05655	0.31481	0.00669	1858.5	19.3	1807.7	9.7	1764.3	32.8	-2.4	1858.5	19.3
QL2 090	43	141	0.3	0.11486	0.00138	5.33688	0.06675	0.33702	0.00720	1877.7	21.5	1874.8	10.7	1872.3	34.7	-0.1	1877.7	21.5
QL2 007	46	144	0.3	0.11495	0.00151	5.42867	0.07075	0.34248	0.00726	1879.0	23.5	1889.4	11.2	1898.6	34.9	0.5	1879.0	23.5
QL2 016	151	320	0.5	0.11738	0.00130	5.59109	0.06409	0.34531	0.00728	1916.6	19.7	1914.7	9.9	1912.2	34.9	-0.1	1916.6	19.7
QL2 026	173	218	0.8	0.11767	0.00150	4.70118	0.06064	0.28971	0.00604	1921.1	22.7	1767.4	10.8	1640.1	30.2	-7.2	1921.1	22.7
QL2 073	46	52	0.9	0.12081	0.00258	5.17038	0.10621	0.31037	0.00713	1968.3	37.6	1847.8	17.5	1742.5	35.1	-5.7	1968.3	37.6
QL2 076	459	635	0.7	0.12291	0.00123	5.61312	0.06057	0.33120	0.00694	1998.9	17.7	1918.1	9.3	1844.2	33.6	-3.9	1998.9	17.7

QL2 072	111	223	0.5	0.15678	0.00235	6.03123	0.08913	0.27898	0.00609	2421.3	25.3	1980.4	12.9	1586.2	30.7	-19.9	2421.3	25.3
~~QL2 033~~	~~162~~	~~366~~	~~0.4~~	~~0.16136~~	~~0.00190~~	~~6.08449~~	~~0.07298~~	~~0.27342~~	~~0.00571~~	~~2469.9~~	~~19.7~~	~~1988.0~~	~~10.5~~	~~1558.1~~	~~28.9~~	~~-36.9~~	~~2469.9~~	~~19.7~~
QL2 101	121	113	1.1	0.16895	0.00187	10.65850	0.12496	0.45768	0.00981	2547.2	18.5	2493.8	10.9	2429.3	43.4	-2.6	2547.2	18.5
QL2 054	124	328	0.4	0.16898	0.00178	10.03410	0.11156	0.43059	0.00899	2547.5	17.5	2437.9	10.3	2308.4	40.5	-5.3	2547.5	17.5
QL2 035	206	458	0.4	0.17425	0.00177	9.35785	0.10079	0.38941	0.00804	2598.9	16.9	2373.7	9.9	2120.1	37.3	-10.7	2598.9	16.9
QL2 039	132	263	0.5	0.17828	0.00277	10.17680	0.15498	0.41390	0.00905	2637.0	25.6	2451.0	14.1	2232.7	41.3	-8.9	2637.0	25.6
QL2 028	418	755	0.6	0.17954	0.00207	11.24222	0.13341	0.45404	0.00947	2648.6	19.0	2543.4	11.1	2413.2	42.0	-5.1	2648.6	19.0
QL2 098	47	86	0.5	0.18862	0.00213	13.48202	0.16011	0.51852	0.01114	2730.2	18.5	2714.0	11.2	2692.9	47.3	-0.8	2730.2	18.5
~~QL2 027~~	~~36~~	~~66~~	~~0.5~~	~~0.19865~~	~~0.00514~~	~~10.09724~~	~~0.24213~~	~~0.36856~~	~~0.00932~~	~~2815.2~~	~~41.7~~	~~2443.7~~	~~22.2~~	~~2022.6~~	~~43.9~~	~~-28.2~~	~~2815.2~~	~~41.7~~

FZ1 009	115	134	0.9	0.05091	0.00147	0.26504	0.00707	0.03775	0.00058	236.9	65.2	238.7	5.7	238.9	3.6	-0.1	238.9	3.6
FZ1 021	330	486	0.7	0.05324	0.00090	0.28191	0.00440	0.03840	0.00048	338.8	37.7	252.2	3.5	242.9	3.0	3.7	242.9	3.0
FZ1 056	120	177	0.7	0.05060	0.00172	0.26817	0.00843	0.03844	0.00066	222.8	77.0	241.2	6.8	243.2	4.1	-0.8	243.2	4.1
FZ1 001	140	136	1.0	0.05055	0.00103	0.26863	0.00513	0.03854	0.00050	220.2	46.5	241.6	4.1	243.8	3.1	-0.9	243.8	3.1
FZ1 003	278	468	0.6	0.04950	0.00053	0.26948	0.00275	0.03948	0.00044	171.4	24.9	242.3	2.2	249.6	2.8	-3.0	249.6	2.8
~~FZ1 004~~	~~256~~	~~451~~	~~1.8~~	~~0.09240~~	~~0.00172~~	~~0.50329~~	~~0.00817~~	~~0.03950~~	~~0.00056~~	~~1475.5~~	~~34.9~~	~~413.9~~	~~5.5~~	~~249.7~~	~~3.5~~	~~39.7~~	~~249.7~~	~~3.5~~
FZ1 089	160	365	0.4	0.05118	0.00106	0.28269	0.00541	0.04007	0.00053	249.1	46.8	252.8	4.3	253.3	3.3	-0.2	253.3	3.3
FZ1 061	218	279	0.8	0.05147	0.00110	0.28666	0.00571	0.04040	0.00054	262.1	48.4	255.9	4.5	255.3	3.3	0.2	255.3	3.3
FZ1 006	201	475	0.4	0.05092	0.00087	0.28550	0.00451	0.04066	0.00051	237.2	38.8	255.0	3.6	256.9	3.2	-0.7	256.9	3.2
FZ1 055	60	143	0.4	0.05163	0.00195	0.29311	0.01018	0.04118	0.00076	269.2	84.4	261.0	8.0	260.1	4.7	0.3	260.1	4.7
FZ1 081	127	124	1.0	0.05283	0.00190	0.29986	0.00994	0.04118	0.00072	321.3	79.7	266.3	7.8	260.1	4.5	2.3	260.1	4.5
FZ1 002	129	177	0.7	0.05422	0.00091	0.30802	0.00479	0.04120	0.00051	380.0	37.2	272.6	3.7	260.3	3.2	4.5	260.3	3.2
FZ1 071	201	428	0.5	0.05158	0.00115	0.29315	0.00605	0.04123	0.00057	266.8	50.5	261.0	4.8	260.4	3.5	0.2	260.4	3.5
FZ1 098	337	556	0.6	0.05566	0.00129	0.31648	0.00669	0.04126	0.00059	438.3	50.4	279.2	5.2	260.6	3.6	6.7	260.6	3.6
FZ1 044	160	385	0.4	0.05285	0.00237	0.30132	0.01233	0.04135	0.00089	322.3	98.6	267.4	9.6	261.2	5.5	2.3	261.2	5.5
FZ1 051	79	141	0.6	0.05063	0.00190	0.28860	0.01009	0.04135	0.00073	223.9	84.7	257.5	8.0	261.2	4.5	-1.4	261.2	4.5
FZ1 059	179	324	0.6	0.05161	0.00081	0.29417	0.00432	0.04134	0.00050	268.2	35.8	261.8	3.4	261.2	3.1	0.2	261.2	3.1
FZ1 097	367	324	1.1	0.05180	0.00078	0.29531	0.00416	0.04137	0.00049	276.4	34.2	262.7	3.3	261.3	3.0	0.5	261.3	3.0
FZ1 053	69	55	1.2	0.05227	0.00477	0.29886	0.02543	0.04147	0.00145	297.3	195.1	265.5	19.9	261.9	9.0	1.4	261.9	9.0
FZ1 038	292	732	0.4	0.05745	0.00102	0.32862	0.00532	0.04149	0.00053	508.4	38.8	288.5	4.1	262.0	3.3	9.2	262.0	3.3
FZ1 045	109	152	0.7	0.05061	0.00121	0.28956	0.00642	0.04150	0.00058	223.2	54.3	258.2	5.1	262.1	3.6	-1.5	262.1	3.6

FZ1 042	86	92	0.9	0.05167	0.00283	0.29576	0.01490	0.04151	0.00101	271.0	120.8	263.1	11.7	262.2	6.2	0.3	262.2	6.2
FZ1 096	155	168	0.9	0.05321	0.00173	0.30456	0.00911	0.04153	0.00069	337.8	72.0	270.0	7.1	262.3	4.3	2.9	262.3	4.3
FZ1 034	82	105	0.8	0.05178	0.00153	0.29668	0.00813	0.04155	0.00064	275.9	66.2	263.8	6.4	262.4	4.0	0.5	262.4	4.0
FZ1 079	301	552	0.5	0.05817	0.00100	0.33308	0.00525	0.04154	0.00052	535.8	37.7	291.9	4.0	262.4	3.3	10.1	262.4	3.3
FZ1 057	138	232	0.6	0.05340	0.00082	0.30600	0.00439	0.04156	0.00050	345.8	34.3	271.1	3.4	262.5	3.1	3.2	262.5	3.1
FZ1 058	292	255	1.1	0.05330	0.00114	0.30555	0.00605	0.04158	0.00056	341.4	47.8	270.7	4.7	262.6	3.5	3.0	262.6	3.5
FZ1 060	432	663	0.7	0.05124	0.00100	0.29389	0.00527	0.04160	0.00054	251.8	44.1	261.6	4.1	262.7	3.4	-0.4	262.7	3.4
FZ1 063	137	241	0.6	0.05207	0.00125	0.29864	0.00671	0.04160	0.00057	288.6	53.8	265.3	5.2	262.7	3.5	1.0	262.7	3.5
FZ1 007	112	89	1.3	0.05316	0.00223	0.30502	0.01188	0.04161	0.00079	335.6	92.1	270.3	9.3	262.8	4.9	2.8	262.8	4.9
FZ1 047	42	44	1.0	0.05286	0.00306	0.30339	0.01652	0.04163	0.00094	322.7	126.0	269.0	12.9	262.9	5.8	2.3	262.9	5.8
FZ1 048	225	243	0.9	0.05146	0.00089	0.29660	0.00475	0.04180	0.00052	261.7	39.1	263.7	3.7	264.0	3.2	-0.1	264.0	3.2
FZ1 062	260	533	0.5	0.05114	0.00147	0.29483	0.00784	0.04182	0.00065	246.9	65.0	262.4	6.1	264.1	4.0	-0.6	264.1	4.0
FZ1 072	128	164	0.8	0.05447	0.00098	0.31428	0.00526	0.04186	0.00052	390.3	39.6	277.5	4.1	264.3	3.2	4.8	264.3	3.2
FZ1 073	122	181	0.7	0.05371	0.00098	0.30986	0.00523	0.04185	0.00053	359.0	41.1	274.1	4.1	264.3	3.3	3.6	264.3	3.3
FZ1 065	184	298	0.6	0.05207	0.00083	0.30187	0.00450	0.04205	0.00051	288.7	36.1	267.9	3.5	265.5	3.2	0.9	265.5	3.2
FZ1 013	201	281	0.7	0.05130	0.00084	0.29774	0.00451	0.04209	0.00052	254.3	37.0	264.6	3.5	265.8	3.2	-0.5	265.8	3.2
FZ1 082	65	152	0.4	0.05205	0.00128	0.30226	0.00693	0.04213	0.00058	287.7	55.2	268.2	5.4	266.0	3.6	0.8	266.0	3.6
FZ1 085	214	228	0.9	0.05167	0.00076	0.30053	0.00416	0.04220	0.00050	270.7	33.6	266.8	3.3	266.5	3.1	0.1	266.5	3.1
FZ1 028	383	209	1.8	0.05613	0.00167	0.32855	0.00892	0.04245	0.00069	457.3	64.8	288.5	6.8	268.0	4.3	7.1	268.0	4.3
FZ1 070	470	411	1.1	0.05488	0.00092	0.32176	0.00496	0.04253	0.00053	407.3	36.7	283.3	3.8	268.5	3.3	5.2	268.5	3.3
FZ1 037	139	160	0.9	0.04969	0.00094	0.29255	0.00517	0.04270	0.00054	180.6	43.4	260.6	4.1	269.5	3.3	-3.4	269.5	3.3
FZ1 019	154	261	0.6	0.05164	0.00087	0.30405	0.00478	0.04270	0.00053	269.3	38.3	269.6	3.7	269.6	3.3	0.0	269.6	3.3
FZ1 080	95	138	0.7	0.05083	0.00106	0.29968	0.00585	0.04277	0.00055	233.0	47.3	266.2	4.6	270.0	3.4	-1.4	270.0	3.4
FZ1 084	215	290	0.7	0.05753	0.00229	0.33948	0.01229	0.04281	0.00086	511.4	85.6	296.8	9.3	270.2	5.3	9.0	270.2	5.3
FZ1 093	58	142	0.4	0.05262	0.00105	0.31041	0.00576	0.04280	0.00055	312.3	44.5	274.5	4.5	270.2	3.4	1.6	270.2	3.4
FZ1 078	292	393	0.7	0.05272	0.00069	0.31136	0.00381	0.04284	0.00049	316.7	29.4	275.2	3.0	270.4	3.1	1.7	270.4	3.1
FZ1 014	247	321	0.8	0.05235	0.00069	0.31027	0.00381	0.04298	0.00050	300.8	29.6	274.4	3.0	271.3	3.1	1.1	271.3	3.1
FZ1 012	187	216	0.9	0.05512	0.00085	0.32809	0.00473	0.04316	0.00052	417.1	34.0	288.1	3.6	272.4	3.2	5.4	272.4	3.2
FZ1 008	99	174	0.6	0.05037	0.00148	0.30089	0.00823	0.04332	0.00067	212.2	66.8	267.1	6.4	273.4	4.1	-2.4	273.4	4.1
FZ1 086	85	118	0.7	0.05453	0.00190	0.32651	0.01044	0.04344	0.00076	393.1	75.5	286.9	8.0	274.1	4.7	4.5	274.1	4.7
FZ1 052	198	235	0.8	0.05374	0.00077	0.32225	0.00432	0.04349	0.00052	360.2	32.2	283.6	3.3	274.4	3.2	3.2	274.4	3.2

FZ1 031	246	253	1.0	0.05286	0.00073	0.31746	0.00409	0.04356	0.00051	322.8	31.0	279.9	3.2	274.8	3.2	1.8	274.8	3.2
FZ1 041	129	154	0.8	0.05120	0.00084	0.30760	0.00475	0.04358	0.00053	249.8	37.5	272.3	3.7	275.0	3.3	-1.0	275.0	3.3
FZ1 046	242	299	0.8	0.05380	0.00094	0.32357	0.00521	0.04362	0.00055	362.6	38.9	284.6	4.0	275.2	3.4	3.3	275.2	3.4
FZ1 015	115	169	0.7	0.05440	0.00089	0.32788	0.00495	0.04371	0.00054	387.6	36.0	287.9	3.8	275.8	3.3	4.2	275.8	3.3
FZ1 032	62	59	1.1	0.05067	0.00238	0.30586	0.01341	0.04378	0.00088	226.0	104.9	271.0	10.4	276.2	5.4	-1.9	276.2	5.4
FZ1 074	285	337	0.8	0.05440	0.00076	0.32843	0.00426	0.04379	0.00051	387.8	30.8	288.4	3.3	276.3	3.2	4.2	276.3	3.2
FZ1 011	137	154	0.9	0.05294	0.00100	0.31979	0.00562	0.04380	0.00056	326.4	42.3	281.7	4.3	276.4	3.5	1.9	276.4	3.5
FZ1 022	119	129	0.9	0.05813	0.00233	0.35153	0.01289	0.04386	0.00087	534.2	86.1	305.9	9.7	276.7	5.4	9.5	276.7	5.4
FZ1 026	57	72	0.8	0.05099	0.00234	0.30836	0.01313	0.04386	0.00089	240.3	102.3	272.9	10.2	276.7	5.5	-1.4	276.7	5.5
FZ1 018	202	207	1.0	0.05207	0.00127	0.31530	0.00708	0.04391	0.00064	288.5	54.8	278.3	5.5	277.0	3.9	0.5	277.0	3.9
FZ1 064	65	103	0.6	0.05468	0.00159	0.33128	0.00887	0.04394	0.00069	400.1	62.3	290.5	6.8	277.2	4.2	4.6	277.2	4.2
FZ1 068	120	152	0.8	0.05198	0.00094	0.31480	0.00534	0.04393	0.00055	284.4	41.0	277.9	4.1	277.2	3.4	0.3	277.2	3.4
FZ1 029	124	148	0.8	0.05287	0.00104	0.32080	0.00586	0.04401	0.00057	323.1	43.9	282.5	4.5	277.6	3.5	1.7	277.6	3.5
FZ1 027	105	485	0.2	0.05019	0.00052	0.30556	0.00304	0.04415	0.00049	203.7	24.1	270.7	2.4	278.5	3.1	-2.9	278.5	3.1
FZ1 033	152	190	0.8	0.05125	0.00080	0.31223	0.00453	0.04418	0.00053	252.3	35.3	275.9	3.5	278.7	3.3	-1.0	278.7	3.3
FZ1 066	238	229	1.0	0.05137	0.00077	0.31298	0.00441	0.04420	0.00053	257.3	34.2	276.5	3.4	278.8	3.2	-0.8	278.8	3.2
FZ1 099	259	260	1.0	0.05878	0.00143	0.35839	0.00798	0.04424	0.00064	559.0	52.1	311.0	6.0	279.0	4.0	10.3	279.0	4.0
FZ1 040	132	143	0.9	0.05363	0.00097	0.32715	0.00551	0.04424	0.00056	355.6	40.6	287.4	4.2	279.1	3.5	2.9	279.1	3.5
FZ1 020	205	192	1.1	0.05035	0.00076	0.30814	0.00433	0.04439	0.00053	211.1	34.4	272.7	3.4	280.0	3.3	-2.7	280.0	3.3
FZ1 095	62	87	0.7	0.05548	0.00140	0.34049	0.00797	0.04452	0.00064	431.5	54.9	297.5	6.0	280.8	3.9	5.6	280.8	3.9
FZ1 023	90	129	0.7	0.05126	0.00110	0.31489	0.00628	0.04456	0.00059	252.3	48.5	278.0	4.9	281.0	3.7	-1.1	281.0	3.7
FZ1 100	110	127	0.9	0.05306	0.00141	0.32632	0.00799	0.04462	0.00066	331.4	59.0	286.8	6.1	281.4	4.1	1.9	281.4	4.1
FZ1 050	59	85	0.7	0.05282	0.00123	0.32645	0.00708	0.04483	0.00061	321.1	51.9	286.9	5.4	282.7	3.8	1.5	282.7	3.8
FZ1 025	162	163	1.0	0.05281	0.00083	0.32697	0.00480	0.04490	0.00055	320.7	35.4	287.3	3.7	283.1	3.4	1.5	283.1	3.4
FZ1 088	91	123	0.7	0.05284	0.00106	0.32919	0.00611	0.04519	0.00059	322.1	44.7	288.9	4.7	285.0	3.6	1.3	285.0	3.6
FZ1 049	124	260	0.5	0.05237	0.00098	0.32844	0.00569	0.04548	0.00059	301.8	42.1	288.4	4.4	286.7	3.6	0.6	286.7	3.6
FZ1 017	165	176	0.9	0.05162	0.00091	0.32633	0.00536	0.04585	0.00057	268.7	39.9	286.8	4.1	289.0	3.5	-0.8	289.0	3.5
FZ1 030	56	81	0.7	0.05177	0.00135	0.32778	0.00796	0.04592	0.00067	275.1	58.8	287.9	6.1	289.4	4.1	-0.5	289.4	4.1
FZ1 005	379	325	1.2	0.05079	0.00059	0.32190	0.00350	0.04596	0.00052	231.3	26.4	283.4	2.7	289.7	3.2	-2.2	289.7	3.2
FZ1 035	129	323	0.4	0.05182	0.00076	0.32847	0.00446	0.04597	0.00055	277.5	33.1	288.4	3.4	289.7	3.4	-0.5	289.7	3.4
FZ1 054	255	388	0.7	0.05227	0.00064	0.33351	0.00385	0.04628	0.00053	297.1	27.9	292.2	2.9	291.7	3.3	0.2	291.7	3.3

FZ1 076	110	136	0.8	0.05225	0.00102	0.33668	0.00619	0.04674	0.00059	296.4	44.1	294.7	4.7	294.5	3.6	0.1	294.5	3.6
FZ1 010	80	114	0.7	0.05264	0.00098	0.34005	0.00588	0.04685	0.00060	313.3	41.7	297.2	4.5	295.1	3.7	0.7	295.1	3.7
FZ1 091	40	69	0.6	0.05387	0.00171	0.34948	0.01020	0.04707	0.00077	365.4	69.7	304.3	7.7	296.5	4.7	2.6	296.5	4.7
FZ1 039	36	68	0.5	0.05072	0.00164	0.33119	0.00996	0.04736	0.00076	227.9	72.9	290.5	7.6	298.3	4.7	-2.7	298.3	4.7
FZ1 077	137	249	0.6	0.05237	0.00076	0.34931	0.00474	0.04839	0.00057	301.6	32.6	304.2	3.6	304.6	3.5	-0.1	304.6	3.5
FZ1 090	31	83	0.4	0.05409	0.00137	0.38158	0.00901	0.05118	0.00073	374.5	56.0	328.2	6.6	321.8	4.5	2.0	321.8	4.5
FZ1 067	46	117	0.4	0.05168	0.00100	0.36698	0.00667	0.05151	0.00065	271.4	43.8	317.4	5.0	323.8	4.0	-2.0	323.8	4.0
FZ1 069	41	38	1.1	0.05595	0.00221	0.50989	0.01878	0.06611	0.00120	450.0	85.3	418.4	12.6	412.7	7.3	1.4	412.7	7.3
FZ1 043	40	118	0.3	0.05796	0.00087	0.53826	0.00753	0.06735	0.00081	528.0	33.0	437.3	5.0	420.2	4.9	3.9	420.2	4.9
FZ1 075	36	129	0.3	0.05634	0.00094	0.52633	0.00812	0.06777	0.00084	465.1	36.7	429.4	5.4	422.7	5.0	1.6	422.7	5.0
FZ1 036	103	364	0.3	0.05635	0.00056	0.53624	0.00503	0.06902	0.00077	465.4	21.9	435.9	3.3	430.2	4.6	1.3	430.2	4.6
FZ1 024	193	150	1.3	0.05520	0.00077	0.52682	0.00690	0.06921	0.00082	420.3	30.8	429.7	4.6	431.4	5.0	-0.4	431.4	5.0
FZ1 016	44	104	0.4	0.05372	0.00123	0.51459	0.01088	0.06947	0.00097	359.3	50.9	421.5	7.3	432.9	5.9	-2.7	432.9	5.9
FZ1 087	41	249	0.2	0.05154	0.00069	0.50106	0.00631	0.07053	0.00082	264.9	30.6	412.4	4.3	439.4	4.9	-6.5	439.4	4.9
FZ1 092	122	378	0.3	0.05404	0.00060	0.54334	0.00573	0.07295	0.00082	372.5	25.1	440.6	3.8	453.9	4.9	-3.0	453.9	4.9
FZ1 083	91	109	0.8	0.11429	0.00083	5.18279	0.03588	0.32898	0.00359	1868.8	13.1	1849.8	5.9	1833.4	17.4	1.9	1868.8	13.1
FZ1 094	109	155	0.7	0.13856	0.00091	6.67432	0.04211	0.34948	0.00377	2209.3	11.4	2069.2	5.6	1932.1	18.0	12.5	2209.3	11.4
FZ2 043	113	82	1.4	0.05055	0.00297	0.26917	0.01490	0.03863	0.00090	220.2	130.5	242.0	11.9	244.3	5.6	-1.0	244.3	5.6
FZ2 040	69	184	0.4	0.05119	0.00173	0.28154	0.00887	0.03990	0.00067	249.3	76.1	251.9	7.0	252.2	4.2	-0.1	252.2	4.2
FZ2 075	282	285	1.0	0.05135	0.00173	0.28540	0.00889	0.04032	0.00069	256.6	75.7	254.9	7.0	254.8	4.3	0.0	254.8	4.3
FZ2 042	187	151	1.2	0.05355	0.00211	0.30420	0.01111	0.04120	0.00078	352.3	86.5	269.7	8.7	260.3	4.8	3.5	260.3	4.8
FZ2 090	215	202	1.1	0.06153	0.00238	0.35024	0.01233	0.04129	0.00083	657.7	81.0	304.9	9.3	260.9	5.1	14.4	260.9	5.1
FZ2 064	74	95	0.8	0.05728	0.00317	0.32658	0.01653	0.04136	0.00107	501.9	117.7	286.9	12.7	261.2	6.6	9.0	261.2	6.6
FZ2 002	75	137	0.5	0.05359	0.00366	0.30609	0.01918	0.04142	0.00126	353.9	146.7	271.1	14.9	261.6	7.8	3.5	261.6	7.8
FZ2 083	252	134	1.9	0.05311	0.00214	0.30382	0.01135	0.04149	0.00079	333.6	88.7	269.4	8.8	262.1	4.9	2.7	262.1	4.9
FZ2 073	52	151	0.3	0.05231	0.00223	0.29939	0.01186	0.04152	0.00083	299.1	94.6	265.9	9.3	262.2	5.1	1.4	262.2	5.1
FZ2 039	170	293	0.6	0.05506	0.00221	0.31576	0.01158	0.04160	0.00084	414.6	86.7	278.6	8.9	262.7	5.2	5.7	262.7	5.2
FZ2 065	120	226	0.5	0.05553	0.00172	0.32125	0.00914	0.04196	0.00069	433.5	67.1	282.9	7.0	265.0	4.3	6.3	265.0	4.3
FZ2 100	85	170	0.5	0.05331	0.00286	0.30856	0.01521	0.04199	0.00105	341.9	116.9	273.1	11.8	265.2	6.5	2.9	265.2	6.5
FZ2 066	46	72	0.6	0.05571	0.00500	0.32369	0.02702	0.04215	0.00155	440.4	188.6	284.7	20.7	266.1	9.6	6.5	266.1	9.6

FZ2 011	83	203	0.4	0.05163	0.00161	0.30021	0.00863	0.04217	0.00069	269.1	69.8	266.6	6.7	266.3	4.3	0.1	266.3	4.3
FZ2 012	79	179	0.4	0.05239	0.00202	0.30556	0.01088	0.04230	0.00080	302.5	85.6	270.7	8.5	267.1	5.0	1.3	267.1	5.0
FZ2 048	143	235	0.6	0.05411	0.00149	0.31602	0.00804	0.04237	0.00065	375.3	60.6	278.8	6.2	267.5	4.0	4.1	267.5	4.0
FZ2 020	152	305	0.5	0.05094	0.00132	0.29824	0.00715	0.04246	0.00062	238.3	58.5	265.0	5.6	268.1	3.8	-1.2	268.1	3.8
FZ2 095	78	135	0.6	0.05280	0.00223	0.30916	0.01212	0.04248	0.00084	320.0	93.2	273.5	9.4	268.2	5.2	1.9	268.2	5.2
FZ2 017	116	146	0.8	0.04966	0.00176	0.29128	0.00958	0.04254	0.00074	179.1	80.5	259.6	7.5	268.6	4.6	-3.5	268.6	4.6
FZ2 001	121	149	0.8	0.05069	0.00186	0.29782	0.01016	0.04261	0.00076	226.9	82.6	264.7	8.0	269.0	4.7	-1.6	269.0	4.7
FZ2 025	195	853	0.2	0.05113	0.00076	0.30038	0.00417	0.04261	0.00051	246.9	34.0	266.7	3.3	269.0	3.2	-0.9	269.0	3.2
FZ2 079	235	382	0.6	0.05215	0.00127	0.30630	0.00692	0.04261	0.00061	291.8	54.7	271.3	5.4	269.0	3.8	0.8	269.0	3.8
FZ2 015	96	243	0.4	0.05058	0.00144	0.29731	0.00786	0.04264	0.00066	221.7	64.7	264.3	6.2	269.2	4.1	-1.9	269.2	4.1
FZ2 058	158	306	0.5	0.05205	0.00141	0.30643	0.00766	0.04271	0.00064	287.5	60.5	271.4	6.0	269.6	4.0	0.7	269.6	4.0
FZ2 061	130	180	0.7	0.05305	0.00230	0.31260	0.01250	0.04274	0.00089	331.1	95.3	276.2	9.7	269.8	5.5	2.3	269.8	5.5
FZ2 034	120	139	0.9	0.05087	0.00195	0.29991	0.01071	0.04276	0.00078	235.1	86.3	266.3	8.4	269.9	4.8	-1.4	269.9	4.8
FZ2 023	118	196	0.6	0.05155	0.00160	0.30383	0.00876	0.04275	0.00069	265.3	69.8	269.4	6.8	269.9	4.3	-0.2	269.9	4.3
FZ2 005	98	125	0.8	0.05502	0.00224	0.32452	0.01218	0.04278	0.00084	412.8	87.9	285.4	9.3	270.1	5.2	5.4	270.1	5.2
FZ2 085	134	175	0.8	0.05389	0.00235	0.31834	0.01280	0.04285	0.00090	366.5	95.0	280.6	9.9	270.5	5.6	3.6	270.5	5.6
FZ2 087	105	160	0.7	0.05196	0.00187	0.30770	0.01034	0.04296	0.00075	283.8	80.3	272.4	8.0	271.1	4.6	0.5	271.1	4.6
FZ2 018	63	107	0.6	0.05094	0.00227	0.30224	0.01262	0.04304	0.00084	237.9	99.6	268.1	9.8	271.6	5.2	-1.3	271.6	5.2
FZ2 028	103	330	0.3	0.05076	0.00119	0.30141	0.00656	0.04307	0.00060	230.0	53.2	267.5	5.1	271.8	3.7	-1.6	271.8	3.7
FZ2 098	94	212	0.4	0.05311	0.00191	0.31529	0.01056	0.04307	0.00076	333.3	79.7	278.3	8.2	271.8	4.7	2.3	271.8	4.7
FZ2 059	66	130	0.5	0.05095	0.00212	0.30275	0.01173	0.04310	0.00082	238.8	93.0	268.5	9.1	272.0	5.1	-1.3	272.0	5.1
FZ2 093	105	227	0.5	0.05205	0.00161	0.30938	0.00889	0.04312	0.00069	287.6	69.0	273.7	6.9	272.1	4.3	0.6	272.1	4.3
FZ2 009	149	176	0.8	0.05321	0.00191	0.31641	0.01050	0.04313	0.00078	337.9	79.4	279.1	8.1	272.2	4.8	2.5	272.2	4.8
FZ2 068	313	456	0.7	0.05151	0.00115	0.30634	0.00633	0.04315	0.00059	263.5	50.3	271.3	4.9	272.3	3.7	-0.4	272.3	3.7
FZ2 077	89	133	0.7	0.05580	0.00257	0.33238	0.01418	0.04321	0.00092	444.1	99.8	291.4	10.8	272.7	5.7	6.4	272.7	5.7
FZ2 082	129	241	0.5	0.05589	0.00175	0.33330	0.00963	0.04326	0.00072	447.6	68.3	292.1	7.3	273.0	4.5	6.5	273.0	4.5
FZ2 054	132	240	0.6	0.05123	0.00152	0.30584	0.00844	0.04330	0.00068	251.4	66.9	270.9	6.6	273.3	4.2	-0.9	273.3	4.2
FZ2 096	90	115	0.8	0.06375	0.00266	0.38072	0.01464	0.04333	0.00088	733.2	86.0	327.6	10.8	273.4	5.4	16.5	273.4	5.4
FZ2 084	204	240	0.9	0.05266	0.00157	0.31476	0.00872	0.04336	0.00069	314.2	66.5	277.9	6.7	273.6	4.3	1.5	273.6	4.3
FZ2 035	199	233	0.9	0.05241	0.00158	0.31347	0.00876	0.04339	0.00070	303.2	67.3	276.9	6.8	273.8	4.3	1.1	273.8	4.3
FZ2 003	171	256	0.7	0.05130	0.00136	0.30708	0.00752	0.04342	0.00065	254.4	59.7	271.9	5.8	274.0	4.0	-0.8	274.0	4.0

FZ2 045	157	261	0.6	0.05316	0.00144	0.31828	0.00799	0.04343	0.00066	335.5	60.5	280.6	6.2	274.1	4.1	2.3	274.1	4.1
FZ2 081	68	110	0.6	0.05231	0.00226	0.31378	0.01258	0.04351	0.00088	299.0	95.7	277.1	9.7	274.6	5.4	0.9	274.6	5.4
FZ2 051	134	162	0.8	0.05158	0.00183	0.30971	0.01024	0.04355	0.00076	266.9	79.5	274.0	7.9	274.8	4.7	-0.3	274.8	4.7
FZ2 013	115	226	0.5	0.05196	0.00149	0.31212	0.00828	0.04357	0.00067	283.8	64.1	275.8	6.4	274.9	4.2	0.3	274.9	4.2
FZ2 004	117	148	0.8	0.05313	0.00331	0.31941	0.01835	0.04360	0.00121	334.5	134.7	281.5	14.1	275.1	7.5	2.3	275.1	7.5
FZ2 070	92	140	0.7	0.05414	0.00210	0.32557	0.01164	0.04362	0.00083	376.6	84.4	286.2	8.9	275.3	5.2	3.8	275.3	5.2
FZ2 008	66	213	0.3	0.06201	0.00178	0.37337	0.00978	0.04367	0.00071	674.6	60.3	322.1	7.2	275.5	4.4	14.5	275.5	4.4
FZ2 097	80	121	0.7	0.05086	0.00224	0.30693	0.01270	0.04378	0.00084	234.5	98.5	271.8	9.9	276.2	5.2	-1.6	276.2	5.2
FZ2 053	77	104	0.7	0.04981	0.00234	0.30119	0.01327	0.04387	0.00089	185.9	105.9	267.3	10.4	276.8	5.5	-3.6	276.8	5.5
FZ2 022	96	223	0.4	0.05152	0.00146	0.31206	0.00817	0.04393	0.00068	264.4	63.6	275.8	6.3	277.1	4.2	-0.5	277.1	4.2
FZ2 074	61	106	0.6	0.05855	0.00350	0.35462	0.01939	0.04394	0.00122	550.4	125.4	308.2	14.5	277.2	7.5	10.1	277.2	7.5
FZ2 099	75	140	0.5	0.06872	0.00258	0.41811	0.01426	0.04414	0.00088	890.4	75.8	354.7	10.2	278.4	5.4	21.5	278.4	5.4
FZ2 091	113	197	0.6	0.05170	0.00196	0.31501	0.01106	0.04420	0.00081	272.3	84.4	278.1	8.5	278.8	5.0	-0.3	278.8	5.0
FZ2 069	124	232	0.5	0.05635	0.00219	0.34341	0.01221	0.04421	0.00087	465.6	84.4	299.8	9.2	278.8	5.3	7.0	278.8	5.3
FZ2 014	112	195	0.6	0.05143	0.00152	0.31374	0.00856	0.04425	0.00071	260.0	66.6	277.1	6.6	279.1	4.4	-0.7	279.1	4.4
FZ2 027	87	139	0.6	0.05387	0.00239	0.32989	0.01355	0.04442	0.00093	365.6	96.5	289.5	10.4	280.2	5.7	3.2	280.2	5.7
FZ2 032	143	275	0.5	0.05085	0.00145	0.31184	0.00823	0.04449	0.00068	233.8	64.3	275.6	6.4	280.6	4.2	-1.8	280.6	4.2
FZ2 063	64	147	0.4	0.05237	0.00210	0.32126	0.01206	0.04450	0.00082	301.5	89.1	282.9	9.3	280.7	5.1	0.8	280.7	5.1
FZ2 078	95	159	0.6	0.05288	0.00196	0.32469	0.01114	0.04454	0.00081	323.7	82.1	285.5	8.5	280.9	5.0	1.6	280.9	5.0
FZ2 052	98	187	0.5	0.05318	0.00173	0.32693	0.00984	0.04459	0.00074	336.5	71.9	287.2	7.5	281.2	4.6	2.1	281.2	4.6
FZ2 030	30	79	0.4	0.05247	0.00264	0.32268	0.01506	0.04461	0.00101	305.9	110.7	284.0	11.6	281.3	6.2	1.0	281.3	6.2
FZ2 037	106	253	0.4	0.05188	0.00141	0.32020	0.00810	0.04477	0.00067	280.2	61.0	282.1	6.2	282.3	4.1	-0.1	282.3	4.1
FZ2 089	268	255	1.0	0.05562	0.00159	0.34335	0.00904	0.04478	0.00072	437.1	62.3	299.7	6.8	282.4	4.4	5.8	282.4	4.4
FZ2 086	80	147	0.5	0.05336	0.00236	0.33064	0.01354	0.04495	0.00092	344.2	96.7	290.1	10.3	283.4	5.7	2.3	283.4	5.7
FZ2 016	74	145	0.5	0.05227	0.00183	0.32457	0.01051	0.04504	0.00080	297.4	78.0	285.4	8.1	284.0	5.0	0.5	284.0	5.0
FZ2 094	129	323	0.4	0.05082	0.00132	0.32082	0.00775	0.04580	0.00067	232.5	58.9	282.5	6.0	288.7	4.1	-2.2	288.7	4.1
FZ2 056	181	621	0.3	0.05651	0.00104	0.35907	0.00609	0.04610	0.00060	471.6	40.7	311.5	4.6	290.5	3.7	6.7	290.5	3.7
FZ2 076	89	157	0.6	0.05711	0.00247	0.36302	0.01448	0.04611	0.00096	495.2	93.3	314.5	10.8	290.6	5.9	7.6	290.6	5.9
FZ2 033	87	116	0.7	0.05144	0.00295	0.32734	0.01749	0.04616	0.00114	260.7	126.7	287.5	13.4	290.9	7.1	-1.2	290.9	7.1
FZ2 067	105	119	0.9	0.05343	0.00246	0.34050	0.01448	0.04623	0.00099	346.8	100.5	297.6	11.0	291.4	6.1	2.1	291.4	6.1
FZ2 010	160	551	0.3	0.05307	0.00092	0.34035	0.00548	0.04651	0.00059	331.9	38.9	297.4	4.2	293.1	3.6	1.4	293.1	3.6

FZ2 019	165	696	0.2	0.05077	0.00077	0.32815	0.00466	0.04688	0.00056	230.4	34.8	288.2	3.6	295.3	3.5	-2.5	295.3	3.5
FZ2 036	229	637	0.4	0.05171	0.00085	0.33622	0.00511	0.04716	0.00058	272.6	37.0	294.3	3.9	297.1	3.6	-1.0	297.1	3.6
FZ2 024	196	592	0.3	0.05285	0.00098	0.34449	0.00593	0.04728	0.00061	322.4	41.7	300.6	4.5	297.8	3.8	0.9	297.8	3.8
FZ2 046	114	468	0.2	0.05106	0.00097	0.33502	0.00591	0.04759	0.00062	243.6	43.3	293.4	4.5	299.7	3.8	-2.1	299.7	3.8
FZ2 044	268	676	0.4	0.05426	0.00085	0.35672	0.00516	0.04769	0.00058	381.7	34.7	309.8	3.9	300.3	3.6	3.1	300.3	3.6
FZ2 071	196	649	0.3	0.05461	0.00099	0.35966	0.00601	0.04778	0.00061	396.1	39.7	312.0	4.5	300.9	3.8	3.6	300.9	3.8
FZ2 060	167	534	0.3	0.05308	0.00102	0.35050	0.00622	0.04790	0.00062	332.2	42.8	305.1	4.7	301.6	3.8	1.1	301.6	3.8
FZ2 038	167	509	0.3	0.05374	0.00096	0.35553	0.00589	0.04799	0.00061	360.0	40.1	308.9	4.4	302.2	3.8	2.2	302.2	3.8
FZ2 072	186	580	0.3	0.05303	0.00115	0.35093	0.00701	0.04801	0.00066	329.9	48.2	305.4	5.3	302.3	4.1	1.0	302.3	4.1
FZ2 041	174	576	0.3	0.05103	0.00090	0.33966	0.00559	0.04828	0.00061	242.1	40.3	296.9	4.2	304.0	3.7	-2.4	304.0	3.7
FZ2 080	247	621	0.4	0.05240	0.00092	0.34927	0.00573	0.04835	0.00061	303.0	39.7	304.2	4.3	304.4	3.7	-0.1	304.4	3.7
FZ2 031	158	532	0.3	0.05335	0.00096	0.35931	0.00597	0.04885	0.00062	343.8	40.0	311.7	4.5	307.5	3.8	1.3	307.5	3.8
~~FZ2 021~~	~~43~~	~~88~~	~~0.5~~	~~0.14609~~	~~0.00444~~	~~0.99337~~	~~0.02474~~	~~0.04932~~	~~0.00106~~	~~2300.6~~	~~51.4~~	~~700.4~~	~~12.6~~	~~310.4~~	~~6.5~~	~~55.7~~	~~310.4~~	~~6.5~~
FZ2 029	80	159	0.5	0.05403	0.00171	0.38084	0.01116	0.05112	0.00086	372.3	69.8	327.7	8.2	321.4	5.3	1.9	321.4	5.3
FZ2 047	72	85	0.8	0.05489	0.00211	0.49806	0.01781	0.06582	0.00122	407.8	83.1	410.4	12.1	410.9	7.4	-0.1	410.9	7.4
FZ2 007	224	313	0.7	0.05350	0.00100	0.49537	0.00855	0.06716	0.00087	349.8	41.6	408.6	5.8	419.0	5.3	-2.5	419.0	5.3
FZ2 092	90	192	0.5	0.05699	0.00167	0.53298	0.01441	0.06784	0.00112	490.6	64.1	433.8	9.5	423.1	6.7	2.5	423.1	6.7
FZ2 062	153	282	0.5	0.05448	0.00117	0.51775	0.01030	0.06894	0.00095	390.8	47.2	423.6	6.9	429.8	5.7	-1.5	429.8	5.7
FZ2 055	209	284	0.7	0.06013	0.00120	0.58340	0.01074	0.07038	0.00096	608.4	42.7	466.6	6.9	438.4	5.8	6.0	438.4	5.8
FZ2 057	162	484	0.3	0.07679	0.00118	0.74554	0.01037	0.07043	0.00089	1115.6	30.3	565.6	6.0	438.8	5.4	22.4	438.8	5.4
FZ2 026	158	150	1.1	0.05832	0.00231	0.59710	0.02167	0.07427	0.00154	541.0	85.1	475.4	13.8	461.8	9.2	2.9	461.8	9.2
FZ2 049	62	127	0.5	0.06567	0.00167	0.80286	0.01859	0.08869	0.00140	795.8	52.3	598.4	10.5	547.8	8.3	8.5	547.8	8.3
FZ2 088	122	115	1.1	0.07580	0.00131	1.91438	0.03054	0.18322	0.00250	1089.7	34.3	1086.1	10.6	1084.5	13.6	0.5	1089.7	34.3
FZ2 006	40	73	0.5	0.08479	0.00148	2.61728	0.04200	0.22389	0.00317	1310.6	33.5	1305.5	11.8	1302.4	16.7	0.6	1310.6	33.5
FZ2 050	164	293	0.6	0.09050	0.00086	3.10889	0.02796	0.24918	0.00286	1436.1	18.0	1434.9	6.9	1434.2	14.7	0.1	1436.1	18.0

FZ3 075	321	148	2.2	0.05316	0.00235	0.27351	0.01125	0.03732	0.00075	335.4	96.9	245.5	9.0	236.2	4.6	3.8	236.2	4.6
FZ3 072	269	351	0.8	0.06391	0.00221	0.33392	0.01043	0.03790	0.00071	738.6	71.6	292.6	7.9	239.8	4.4	18.0	239.8	4.4
FZ3 026	169	213	0.8	0.05261	0.00174	0.27599	0.00845	0.03805	0.00064	312.1	73.5	247.5	6.7	240.7	4.0	2.7	240.7	4.0
FZ3 090	190	153	1.2	0.05747	0.00332	0.30623	0.01613	0.03865	0.00104	509.2	122.4	271.3	12.5	244.5	6.5	9.9	244.5	6.5
FZ3 056	103	82	1.3	0.05213	0.00316	0.28482	0.01605	0.03963	0.00103	291.3	132.8	254.5	12.7	250.5	6.4	1.6	250.5	6.4

FZ3 041	103	135	0.8	0.05003	0.00212	0.27532	0.01088	0.03991	0.00076	196.4	95.4	246.9	8.7	252.3	4.7	-2.2	252.3	4.7
~~FZ3 030~~	~~93~~	~~63~~	~~1.5~~	~~0.07310~~	~~0.00436~~	~~0.40373~~	~~0.02192~~	~~0.04006~~	~~0.00113~~	~~1016.6~~	~~116.3~~	~~344.3~~	~~15.9~~	~~253.2~~	~~7.0~~	~~26.5~~	~~253.2~~	~~7.0~~
FZ3 039	767	292	2.6	0.05239	0.00196	0.29121	0.01006	0.04032	0.00075	302.4	83.2	259.5	7.9	254.8	4.7	1.8	254.8	4.7
FZ3 054	83	102	0.8	0.05350	0.00265	0.29784	0.01377	0.04038	0.00088	349.9	108.1	264.7	10.8	255.2	5.4	3.6	255.2	5.4
FZ3 002	42	167	0.3	0.05128	0.00170	0.28621	0.00877	0.04048	0.00069	253.4	74.4	255.6	6.9	255.8	4.3	-0.1	255.8	4.3
FZ3 067	126	74	1.7	0.05260	0.00370	0.29503	0.01947	0.04068	0.00113	311.7	151.9	262.5	15.3	257.1	7.0	2.1	257.1	7.0
FZ3 055	99	123	0.8	0.05350	0.00322	0.30064	0.01668	0.04076	0.00109	349.9	129.9	266.9	13.0	257.6	6.8	3.5	257.6	6.8
FZ3 016	344	274	1.3	0.05203	0.00137	0.29336	0.00715	0.04089	0.00061	286.7	59.2	261.2	5.6	258.4	3.8	1.1	258.4	3.8
FZ3 011	58	40	1.5	0.05205	0.00494	0.29349	0.02653	0.04090	0.00134	287.4	203.1	261.3	20.8	258.4	8.3	1.1	258.4	8.3
FZ3 085	86	250	0.3	0.05216	0.00178	0.29684	0.00934	0.04128	0.00072	292.3	76.0	263.9	7.3	260.8	4.5	1.2	260.8	4.5
FZ3 065	416	174	2.4	0.05619	0.00243	0.31986	0.01272	0.04129	0.00086	459.0	93.9	281.8	9.8	260.8	5.3	7.5	260.8	5.3
FZ3 015	145	107	1.4	0.05008	0.00225	0.28544	0.01194	0.04134	0.00083	198.9	101.0	255.0	9.4	261.1	5.2	-2.4	261.1	5.2
FZ3 024	83	90	0.9	0.05456	0.00348	0.31245	0.01849	0.04153	0.00113	394.4	136.6	276.1	14.3	262.3	7.0	5.0	262.3	7.0
FZ3 066	281	542	0.5	0.05607	0.00149	0.32105	0.00782	0.04153	0.00063	454.7	57.8	282.7	6.0	262.3	3.9	7.2	262.3	3.9
FZ3 082	370	327	1.1	0.05213	0.00142	0.29972	0.00755	0.04171	0.00063	290.9	60.9	266.2	5.9	263.4	3.9	1.1	263.4	3.9
FZ3 053	159	281	0.6	0.05248	0.00146	0.30185	0.00779	0.04172	0.00064	306.5	62.1	267.8	6.1	263.5	4.0	1.6	263.5	4.0
FZ3 006	69	93	0.7	0.05327	0.00336	0.30942	0.01812	0.04213	0.00113	340.1	136.1	273.7	14.1	266.0	7.0	2.8	266.0	7.0
FZ3 079	203	221	0.9	0.05956	0.00190	0.34630	0.01013	0.04218	0.00073	587.5	67.9	301.9	7.6	266.3	4.5	11.8	266.3	4.5
FZ3 014	301	285	1.1	0.05290	0.00133	0.30792	0.00717	0.04222	0.00062	324.3	56.1	272.6	5.6	266.6	3.8	2.2	266.6	3.8
FZ3 013	163	267	0.6	0.05018	0.00129	0.29327	0.00700	0.04239	0.00062	203.5	58.6	261.1	5.5	267.6	3.8	-2.5	267.6	3.8
FZ3 084	158	171	0.9	0.05190	0.00186	0.30409	0.01009	0.04250	0.00075	281.0	79.8	269.6	7.9	268.3	4.7	0.5	268.3	4.7
FZ3 005	156	208	0.7	0.05441	0.00213	0.32102	0.01155	0.04280	0.00084	387.8	85.1	282.7	8.9	270.1	5.2	4.5	270.1	5.2
FZ3 076	139	294	0.5	0.05286	0.00140	0.31215	0.00765	0.04284	0.00064	322.6	59.1	275.8	5.9	270.4	4.0	2.0	270.4	4.0
FZ3 037	116	124	0.9	0.05135	0.00216	0.30353	0.01194	0.04288	0.00081	256.4	93.8	269.2	9.3	270.6	5.0	-0.5	270.6	5.0
FZ3 060	17	26	0.6	0.05490	0.00852	0.32462	0.04739	0.04289	0.00244	407.9	314.3	285.5	36.3	270.7	15.1	5.2	270.7	15.1
FZ3 097	41	39	1.0	0.05360	0.00510	0.31697	0.02849	0.04290	0.00148	354.0	201.7	279.6	22.0	270.8	9.2	3.1	270.8	9.2
FZ3 045	100	180	0.6	0.05333	0.00185	0.31686	0.01014	0.04309	0.00076	343.0	76.5	279.5	7.8	272.0	4.7	2.7	272.0	4.7
FZ3 071	72	131	0.5	0.05113	0.00230	0.30491	0.01278	0.04326	0.00089	246.7	100.5	270.2	9.9	273.0	5.5	-1.0	273.0	5.5
FZ3 040	270	284	1.0	0.05172	0.00131	0.30846	0.00721	0.04326	0.00063	273.1	56.8	273.0	5.6	273.0	3.9	0.0	273.0	3.9
FZ3 008	204	348	0.6	0.05630	0.00184	0.33604	0.01009	0.04329	0.00076	463.5	71.2	294.2	7.7	273.2	4.7	7.1	273.2	4.7
~~FZ3 018~~	~~55~~	~~96~~	~~0.6~~	~~0.07760~~	~~0.00374~~	~~0.46309~~	~~0.01988~~	~~0.04329~~	~~0.00111~~	~~1136.6~~	~~93.0~~	~~386.4~~	~~13.8~~	~~273.2~~	~~6.8~~	~~29.3~~	~~273.2~~	~~6.8~~

FZ3 046	57	91	0.6	0.05315	0.00294	0.31730	0.01634	0.04330	0.00103	335.2	120.3	279.8	12.6	273.3	6.4	2.3	273.3	6.4
FZ3 007	45	44	1.0	0.05646	0.00446	0.33731	0.02482	0.04333	0.00141	469.7	166.8	295.1	18.8	273.5	8.7	7.3	273.5	8.7
FZ3 027	153	176	0.9	0.05271	0.00172	0.31602	0.00955	0.04348	0.00072	316.4	72.4	278.8	7.4	274.4	4.5	1.6	274.4	4.5
FZ3 043	129	161	0.8	0.05164	0.00182	0.31047	0.01019	0.04361	0.00074	269.6	78.6	274.5	7.9	275.1	4.6	-0.2	275.1	4.6
FZ3 080	258	406	0.6	0.05776	0.00176	0.34715	0.00966	0.04360	0.00073	520.4	65.7	302.6	7.3	275.1	4.5	9.1	275.1	4.5
FZ3 062	131	258	0.5	0.05836	0.00229	0.35161	0.01268	0.04370	0.00086	543.2	83.7	305.9	9.5	275.7	5.3	9.9	275.7	5.3
FZ3 098	69	114	0.6	0.06715	0.00242	0.40736	0.01342	0.04401	0.00083	842.3	73.4	347.0	9.7	277.6	5.1	20.0	277.6	5.1
FZ3 063	47	57	0.8	0.05020	0.00387	0.30470	0.02233	0.04403	0.00122	204.1	169.8	270.1	17.4	277.8	7.6	-2.9	277.8	7.6
FZ3 099	85	129	0.7	0.05241	0.00254	0.31919	0.01431	0.04418	0.00098	303.2	106.8	281.3	11.0	278.7	6.0	0.9	278.7	6.0
FZ3 038	202	141	1.4	0.05165	0.00196	0.31565	0.01120	0.04433	0.00080	269.8	84.9	278.6	8.6	279.6	4.9	-0.4	279.6	4.9
FZ3 036	157	241	0.7	0.05546	0.00161	0.33929	0.00905	0.04437	0.00071	430.5	63.1	296.6	6.9	279.9	4.4	5.6	279.9	4.4
FZ3 031	175	269	0.7	0.05252	0.00135	0.32240	0.00767	0.04452	0.00066	308.2	57.5	283.7	5.9	280.8	4.1	1.0	280.8	4.1
FZ3 096	23	33	0.7	0.05382	0.00722	0.33054	0.04135	0.04455	0.00234	363.5	277.1	290.0	31.6	281.0	14.4	3.1	281.0	14.4
FZ3 028	66	163	0.4	0.05573	0.00186	0.34239	0.01053	0.04456	0.00078	441.2	72.6	299.0	8.0	281.0	4.8	6.0	281.0	4.8
FZ3 095	84	154	0.5	0.05365	0.00251	0.33222	0.01434	0.04492	0.00098	356.3	102.0	291.3	10.9	283.2	6.1	2.8	283.2	6.1
FZ3 052	138	258	0.5	0.05290	0.00144	0.32984	0.00830	0.04523	0.00068	324.4	60.4	289.4	6.3	285.1	4.2	1.5	285.1	4.2
FZ3 020	141	223	0.6	0.05390	0.00208	0.33804	0.01206	0.04549	0.00087	366.6	84.7	295.7	9.2	286.8	5.4	3.0	286.8	5.4
FZ3 012	83	82	1.0	0.05262	0.00322	0.33177	0.01884	0.04573	0.00121	312.3	133.2	290.9	14.4	288.3	7.5	0.9	288.3	7.5
FZ3 035	98	127	0.8	0.05162	0.00196	0.32717	0.01155	0.04597	0.00084	268.6	84.8	287.4	8.8	289.7	5.2	-0.8	289.7	5.2
FZ3 001	129	109	1.2	0.05255	0.00224	0.34344	0.01361	0.04740	0.00094	309.5	94.3	299.8	10.3	298.5	5.8	0.4	298.5	5.8
FZ3 034	109	125	0.9	0.05325	0.00383	0.34901	0.02321	0.04754	0.00149	339.3	154.7	304.0	17.5	299.4	9.1	1.5	299.4	9.1
FZ3 073	175	153	1.1	0.05408	0.00232	0.35447	0.01402	0.04755	0.00098	374.1	93.1	308.1	10.5	299.4	6.0	2.8	299.4	6.0
FZ3 069	86	92	0.9	0.05830	0.00351	0.39859	0.02222	0.04959	0.00133	540.2	127.3	340.6	16.1	312.0	8.2	8.4	312.0	8.2
FZ3 019	128	328	0.4	0.05681	0.00151	0.41222	0.01007	0.05263	0.00082	483.4	57.8	350.5	7.2	330.7	5.0	5.6	330.7	5.0
FZ3 086	131	1199	0.1	0.05608	0.00067	0.46955	0.00527	0.06073	0.00069	455.3	26.1	390.9	3.6	380.1	4.2	2.8	380.1	4.2
FZ3 100	62	256	0.2	0.05548	0.00118	0.49255	0.00966	0.06440	0.00088	431.2	46.2	406.6	6.6	402.3	5.3	1.1	402.3	5.3
FZ3 048	34	180	0.2	0.05576	0.00149	0.50144	0.01243	0.06522	0.00100	442.6	58.3	412.7	8.4	407.3	6.0	1.3	407.3	6.0
FZ3 094	71	54	1.3	0.05974	0.00314	0.53738	0.02618	0.06524	0.00158	594.2	110.2	436.7	17.3	407.4	9.6	6.7	407.4	9.6
FZ3 092	176	277	0.6	0.05684	0.00122	0.51496	0.01018	0.06571	0.00091	484.8	46.9	421.8	6.8	410.3	5.5	2.7	410.3	5.5
FZ3 077	64	476	0.1	0.05853	0.00098	0.53482	0.00825	0.06627	0.00083	549.8	36.1	435.0	5.5	413.7	5.0	4.9	413.7	5.0
FZ3 068	101	112	0.9	0.05447	0.00181	0.50218	0.01541	0.06687	0.00117	390.5	72.2	413.2	10.4	417.3	7.1	-1.0	417.3	7.1

FZ3 089	109	327	0.3	0.05540	0.00115	0.51436	0.00983	0.06735	0.00091	428.2	45.0	421.4	6.6	420.1	5.5	0.3	420.1	5.5
FZ3 003	138	207	0.7	0.05546	0.00132	0.51678	0.01139	0.06758	0.00098	430.7	51.8	423.0	7.6	421.6	5.9	0.3	421.6	5.9
FZ3 087	194	273	0.7	0.05605	0.00127	0.52224	0.01090	0.06758	0.00096	454.1	49.3	426.6	7.3	421.6	5.8	1.2	421.6	5.8
FZ3 088	154	227	0.7	0.05608	0.00122	0.52244	0.01050	0.06758	0.00094	455.0	47.4	426.8	7.0	421.6	5.7	1.2	421.6	5.7
FZ3 078	124	271	0.5	0.05552	0.00113	0.51797	0.00976	0.06767	0.00091	432.9	44.3	423.8	6.5	422.1	5.5	0.4	422.1	5.5
FZ3 009	62	258	0.2	0.05612	0.00113	0.52434	0.00973	0.06777	0.00091	456.6	43.8	428.0	6.5	422.7	5.5	1.2	422.7	5.5
FZ3 047	87	206	0.4	0.05578	0.00145	0.52721	0.01267	0.06855	0.00105	443.3	56.8	430.0	8.4	427.4	6.3	0.6	427.4	6.3
FZ3 093	234	427	0.5	0.05500	0.00091	0.52220	0.00804	0.06886	0.00085	412.4	36.3	426.6	5.4	429.3	5.1	-0.6	429.3	5.1
FZ3 051	214	269	0.8	0.05630	0.00117	0.53756	0.01030	0.06926	0.00094	463.4	45.7	436.8	6.8	431.7	5.7	1.2	431.7	5.7
FZ3 057	117	187	0.6	0.05725	0.00162	0.54717	0.01430	0.06932	0.00111	500.8	61.7	443.1	9.4	432.1	6.7	2.5	432.1	6.7
FZ3 021	236	308	0.8	0.05494	0.00103	0.52934	0.00924	0.06989	0.00092	409.5	41.3	431.4	6.1	435.5	5.5	-1.0	435.5	5.5
FZ3 050	199	242	0.8	0.05546	0.00120	0.53485	0.01074	0.06995	0.00097	430.6	47.2	435.0	7.1	435.8	5.9	-0.2	435.8	5.9
FZ3 022	143	292	0.5	0.05654	0.00107	0.54647	0.00960	0.07010	0.00092	472.9	41.9	442.7	6.3	436.8	5.6	1.3	436.8	5.6
FZ3 059	159	288	0.6	0.05740	0.00112	0.55674	0.00999	0.07035	0.00094	506.7	42.6	449.4	6.5	438.2	5.6	2.5	438.2	5.6
FZ3 042	97	220	0.4	0.05636	0.00131	0.54665	0.01171	0.07035	0.00101	465.8	51.1	442.8	7.7	438.3	6.1	1.0	438.3	6.1
FZ3 004	100	259	0.4	0.05551	0.00108	0.54007	0.00975	0.07057	0.00094	432.4	42.4	438.5	6.4	439.6	5.7	-0.3	439.6	5.7
FZ3 044	129	191	0.7	0.05627	0.00135	0.54927	0.01218	0.07080	0.00103	462.4	52.8	444.5	8.0	440.9	6.2	0.8	440.9	6.2
FZ3 070	232	316	0.7	0.05691	0.00139	0.55611	0.01250	0.07088	0.00105	487.5	53.0	449.0	8.2	441.4	6.3	1.7	441.4	6.3
FZ3 032	119	270	0.4	0.05681	0.00126	0.55895	0.01147	0.07136	0.00101	483.5	48.6	450.8	7.5	444.4	6.1	1.4	444.4	6.1
FZ3 010	75	143	0.5	0.05788	0.00163	0.57503	0.01497	0.07206	0.00115	524.8	60.9	461.3	9.7	448.6	6.9	2.8	448.6	6.9
FZ3 049	98	207	0.5	0.05946	0.00148	0.61344	0.01399	0.07483	0.00113	583.9	53.0	485.7	8.8	465.2	6.8	4.2	465.2	6.8
FZ3 025	152	329	0.5	0.05933	0.00122	0.62001	0.01174	0.07580	0.00105	579.1	44.1	489.9	7.4	471.0	6.3	3.9	471.0	6.3
FZ3 023	8	28	0.3	0.05924	0.00569	0.63378	0.05702	0.07760	0.00301	575.8	196.0	498.5	35.4	481.8	18.0	3.4	481.8	18.0
FZ3 083	87	352	0.2	0.06660	0.00105	0.71464	0.01040	0.07783	0.00098	825.4	32.7	547.5	6.2	483.2	5.8	11.7	483.2	5.8
FZ3 033	32	118	0.3	0.05729	0.00259	0.62002	0.02618	0.07850	0.00163	502.2	97.0	489.9	16.4	487.2	9.8	0.6	487.2	9.8
FZ3 017	76	136	0.6	0.05754	0.00152	0.63686	0.01558	0.08028	0.00124	512.0	57.5	500.4	9.7	497.8	7.4	0.5	497.8	7.4
FZ3 074	254	570	0.4	0.06261	0.00081	0.85495	0.01029	0.09904	0.00117	695.2	27.4	627.4	5.6	608.8	6.9	3.0	608.8	6.9
FZ3 029	66	48	1.4	0.06309	0.00225	1.00270	0.03303	0.11527	0.00220	711.3	73.9	705.2	16.8	703.3	12.7	0.3	703.3	12.7
FZ3 061	111	476	0.2	0.06531	0.00089	1.12724	0.01424	0.12519	0.00151	784.4	28.3	766.4	6.8	760.4	8.7	0.8	760.4	8.7
FZ3 091	69	136	0.5	0.06744	0.00188	1.18640	0.03035	0.12762	0.00218	851.2	57.0	794.3	14.1	774.2	12.4	2.5	774.2	12.4
FZ3 058	35	94	0.4	0.07213	0.00192	1.50996	0.03673	0.15185	0.00257	989.5	53.1	934.4	14.9	911.3	14.4	2.5	911.3	14.4

FZ3 081	78	221	0.4	0.08540	0.00113	1.86586	0.02267	0.15847	0.00196	1324.7	25.5	1069.1	8.0	948.3	10.9	11.3	948.3	10.9
FZ3 064	87	111	0.8	0.11748	0.00146	5.40023	0.06271	0.33343	0.00433	1918.2	22.1	1884.9	10.0	1855.0	20.9	3.3	1918.2	22.1
FZ3 075	321	148	2.2	0.05316	0.00235	0.27351	0.01125	0.03732	0.00075	335.4	96.9	245.5	9.0	236.2	4.6	3.8	236.2	4.6
FZ3 072	269	351	0.8	0.06391	0.00221	0.33392	0.01043	0.03790	0.00071	738.6	71.6	292.6	7.9	239.8	4.4	18.0	239.8	4.4
FZ3 026	169	213	0.8	0.05261	0.00174	0.27599	0.00845	0.03805	0.00064	312.1	73.5	247.5	6.7	240.7	4.0	2.7	240.7	4.0
FZ3 090	190	153	1.2	0.05747	0.00332	0.30623	0.01613	0.03865	0.00104	509.2	122.4	271.3	12.5	244.5	6.5	9.9	244.5	6.5
FZ3 056	103	82	1.3	0.05213	0.00316	0.28482	0.01605	0.03963	0.00103	291.3	132.8	254.5	12.7	250.5	6.4	1.6	250.5	6.4
FZ3 041	103	135	0.8	0.05003	0.00212	0.27532	0.01088	0.03991	0.00076	196.4	95.4	246.9	8.7	252.3	4.7	-2.2	252.3	4.7
~~FZ3 030~~	~~93~~	~~63~~	~~1.5~~	~~0.07310~~	~~0.00436~~	~~0.40373~~	~~0.02192~~	~~0.04006~~	~~0.00113~~	~~1016.6~~	~~116.3~~	~~344.3~~	~~15.9~~	~~253.2~~	~~7.0~~	~~26.5~~	~~253.2~~	~~7.0~~
FZ3 039	767	292	2.6	0.05239	0.00196	0.29121	0.01006	0.04032	0.00075	302.4	83.2	259.5	7.9	254.8	4.7	1.8	254.8	4.7
FZ3 054	83	102	0.8	0.05350	0.00265	0.29784	0.01377	0.04038	0.00088	349.9	108.1	264.7	10.8	255.2	5.4	3.6	255.2	5.4
FZ3 002	42	167	0.3	0.05128	0.00170	0.28621	0.00877	0.04048	0.00069	253.4	74.4	255.6	6.9	255.8	4.3	-0.1	255.8	4.3
FZ3 067	126	74	1.7	0.05260	0.00370	0.29503	0.01947	0.04068	0.00113	311.7	151.9	262.5	15.3	257.1	7.0	2.1	257.1	7.0
FZ3 055	99	123	0.8	0.05350	0.00322	0.30064	0.01668	0.04076	0.00109	349.9	129.9	266.9	13.0	257.6	6.8	3.5	257.6	6.8
FZ3 016	344	274	1.3	0.05203	0.00137	0.29336	0.00715	0.04089	0.00061	286.7	59.2	261.2	5.6	258.4	3.8	1.1	258.4	3.8
FZ3 011	58	40	1.5	0.05205	0.00494	0.29349	0.02653	0.04090	0.00134	287.4	203.1	261.3	20.8	258.4	8.3	1.1	258.4	8.3
FZ3 085	86	250	0.3	0.05216	0.00178	0.29684	0.00934	0.04128	0.00072	292.3	76.0	263.9	7.3	260.8	4.5	1.2	260.8	4.5
FZ3 065	416	174	2.4	0.05619	0.00243	0.31986	0.01272	0.04129	0.00086	459.0	93.9	281.8	9.8	260.8	5.3	7.5	260.8	5.3
FZ3 015	145	107	1.4	0.05008	0.00225	0.28544	0.01194	0.04134	0.00083	198.9	101.0	255.0	9.4	261.1	5.2	-2.4	261.1	5.2
FZ3 024	83	90	0.9	0.05456	0.00348	0.31245	0.01849	0.04153	0.00113	394.4	136.6	276.1	14.3	262.3	7.0	5.0	262.3	7.0
FZ3 066	281	542	0.5	0.05607	0.00149	0.32105	0.00782	0.04153	0.00063	454.7	57.8	282.7	6.0	262.3	3.9	7.2	262.3	3.9
FZ3 082	370	327	1.1	0.05213	0.00142	0.29972	0.00755	0.04171	0.00063	290.9	60.9	266.2	5.9	263.4	3.9	1.1	263.4	3.9
FZ3 053	159	281	0.6	0.05248	0.00146	0.30185	0.00779	0.04172	0.00064	306.5	62.1	267.8	6.1	263.5	4.0	1.6	263.5	4.0
FZ3 006	69	93	0.7	0.05327	0.00336	0.30942	0.01812	0.04213	0.00113	340.1	136.1	273.7	14.1	266.0	7.0	2.8	266.0	7.0
FZ3 079	203	221	0.9	0.05956	0.00190	0.34630	0.01013	0.04218	0.00073	587.5	67.9	301.9	7.6	266.3	4.5	11.8	266.3	4.5
FZ3 014	301	285	1.1	0.05290	0.00133	0.30792	0.00717	0.04222	0.00062	324.3	56.1	272.6	5.6	266.6	3.8	2.2	266.6	3.8
FZ3 013	163	267	0.6	0.05018	0.00129	0.29327	0.00700	0.04239	0.00062	203.5	58.6	261.1	5.5	267.6	3.8	-2.5	267.6	3.8
FZ3 084	158	171	0.9	0.05190	0.00186	0.30409	0.01009	0.04250	0.00075	281.0	79.8	269.6	7.9	268.3	4.7	0.5	268.3	4.7
FZ3 005	156	208	0.7	0.05441	0.00213	0.32102	0.01155	0.04280	0.00084	387.8	85.1	282.7	8.9	270.1	5.2	4.5	270.1	5.2
FZ3 076	139	294	0.5	0.05286	0.00140	0.31215	0.00765	0.04284	0.00064	322.6	59.1	275.8	5.9	270.4	4.0	2.0	270.4	4.0
FZ3 037	116	124	0.9	0.05135	0.00216	0.30353	0.01194	0.04288	0.00081	256.4	93.8	269.2	9.3	270.6	5.0	-0.5	270.6	5.0

FZ3 060	17	26	0.6	0.05490	0.00852	0.32462	0.04739	0.04289	0.00244	407.9	314.3	285.5	36.3	270.7	15.1	5.2	270.7	15.1
FZ3 097	41	39	1.0	0.05360	0.00510	0.31697	0.02849	0.04290	0.00148	354.0	201.7	279.6	22.0	270.8	9.2	3.1	270.8	9.2
FZ3 045	100	180	0.6	0.05333	0.00185	0.31686	0.01014	0.04309	0.00076	343.0	76.5	279.5	7.8	272.0	4.7	2.7	272.0	4.7
FZ3 071	72	131	0.5	0.05113	0.00230	0.30491	0.01278	0.04326	0.00089	246.7	100.5	270.2	9.9	273.0	5.5	-1.0	273.0	5.5
FZ3 040	270	284	1.0	0.05172	0.00131	0.30846	0.00721	0.04326	0.00063	273.1	56.8	273.0	5.6	273.0	3.9	0.0	273.0	3.9
FZ3 008	204	348	0.6	0.05630	0.00184	0.33604	0.01009	0.04329	0.00076	463.5	71.2	294.2	7.7	273.2	4.7	7.1	273.2	4.7
~~FZ3 018~~	~~55~~	~~96~~	~~0.6~~	~~0.07760~~	~~0.00374~~	~~0.46309~~	~~0.01988~~	~~0.04329~~	~~0.00111~~	~~1136.6~~	~~93.0~~	~~386.4~~	~~13.8~~	~~273.2~~	~~6.8~~	~~29.3~~	~~273.2~~	~~6.8~~
FZ3 046	57	91	0.6	0.05315	0.00294	0.31730	0.01634	0.04330	0.00103	335.2	120.3	279.8	12.6	273.3	6.4	2.3	273.3	6.4
FZ3 007	45	44	1.0	0.05646	0.00446	0.33731	0.02482	0.04333	0.00141	469.7	166.8	295.1	18.8	273.5	8.7	7.3	273.5	8.7
FZ3 027	153	176	0.9	0.05271	0.00172	0.31602	0.00955	0.04348	0.00072	316.4	72.4	278.8	7.4	274.4	4.5	1.6	274.4	4.5
FZ3 043	129	161	0.8	0.05164	0.00182	0.31047	0.01019	0.04361	0.00074	269.6	78.6	274.5	7.9	275.1	4.6	-0.2	275.1	4.6
FZ3 080	258	406	0.6	0.05776	0.00176	0.34715	0.00966	0.04360	0.00073	520.4	65.7	302.6	7.3	275.1	4.5	9.1	275.1	4.5
FZ3 062	131	258	0.5	0.05836	0.00229	0.35161	0.01268	0.04370	0.00086	543.2	83.7	305.9	9.5	275.7	5.3	9.9	275.7	5.3
FZ3 098	69	114	0.6	0.06715	0.00242	0.40736	0.01342	0.04401	0.00083	842.3	73.4	347.0	9.7	277.6	5.1	20.0	277.6	5.1
FZ3 063	47	57	0.8	0.05020	0.00387	0.30470	0.02233	0.04403	0.00122	204.1	169.8	270.1	17.4	277.8	7.6	-2.9	277.8	7.6
FZ3 099	85	129	0.7	0.05241	0.00254	0.31919	0.01431	0.04418	0.00098	303.2	106.8	281.3	11.0	278.7	6.0	0.9	278.7	6.0
FZ3 038	202	141	1.4	0.05165	0.00196	0.31565	0.01120	0.04433	0.00080	269.8	84.9	278.6	8.6	279.6	4.9	-0.4	279.6	4.9
FZ3 036	157	241	0.7	0.05546	0.00161	0.33929	0.00905	0.04437	0.00071	430.5	63.1	296.6	6.9	279.9	4.4	5.6	279.9	4.4
FZ3 031	175	269	0.7	0.05252	0.00135	0.32240	0.00767	0.04452	0.00066	308.2	57.5	283.7	5.9	280.8	4.1	1.0	280.8	4.1
FZ3 096	23	33	0.7	0.05382	0.00722	0.33054	0.04135	0.04455	0.00234	363.5	277.1	290.0	31.6	281.0	14.4	3.1	281.0	14.4
FZ3 028	66	163	0.4	0.05573	0.00186	0.34239	0.01053	0.04456	0.00078	441.2	72.6	299.0	8.0	281.0	4.8	6.0	281.0	4.8
FZ3 095	84	154	0.5	0.05365	0.00251	0.33222	0.01434	0.04492	0.00098	356.3	102.0	291.3	10.9	283.2	6.1	2.8	283.2	6.1
FZ3 052	138	258	0.5	0.05290	0.00144	0.32984	0.00830	0.04523	0.00068	324.4	60.4	289.4	6.3	285.1	4.2	1.5	285.1	4.2
FZ3 020	141	223	0.6	0.05390	0.00208	0.33804	0.01206	0.04549	0.00087	366.6	84.7	295.7	9.2	286.8	5.4	3.0	286.8	5.4
FZ3 012	83	82	1.0	0.05262	0.00322	0.33177	0.01884	0.04573	0.00121	312.3	133.2	290.9	14.4	288.3	7.5	0.9	288.3	7.5
FZ3 035	98	127	0.8	0.05162	0.00196	0.32717	0.01155	0.04597	0.00084	268.6	84.8	287.4	8.8	289.7	5.2	-0.8	289.7	5.2
FZ3 001	129	109	1.2	0.05255	0.00224	0.34344	0.01361	0.04740	0.00094	309.5	94.3	299.8	10.3	298.5	5.8	0.4	298.5	5.8
FZ3 034	109	125	0.9	0.05325	0.00383	0.34901	0.02321	0.04754	0.00149	339.3	154.7	304.0	17.5	299.4	9.1	1.5	299.4	9.1
FZ3 073	175	153	1.1	0.05408	0.00232	0.35447	0.01402	0.04755	0.00098	374.1	93.1	308.1	10.5	299.4	6.0	2.8	299.4	6.0
FZ3 069	86	92	0.9	0.05830	0.00351	0.39859	0.02222	0.04959	0.00133	540.2	127.3	340.6	16.1	312.0	8.2	8.4	312.0	8.2
FZ3 019	128	328	0.4	0.05681	0.00151	0.41222	0.01007	0.05263	0.00082	483.4	57.8	350.5	7.2	330.7	5.0	5.6	330.7	5.0

FZ3 086	131	1199	0.1	0.05608	0.00067	0.46955	0.00527	0.06073	0.00069	455.3	26.1	390.9	3.6	380.1	4.2	2.8	380.1	4.2
FZ3 100	62	256	0.2	0.05548	0.00118	0.49255	0.00966	0.06440	0.00088	431.2	46.2	406.6	6.6	402.3	5.3	1.1	402.3	5.3
FZ3 048	34	180	0.2	0.05576	0.00149	0.50144	0.01243	0.06522	0.00100	442.6	58.3	412.7	8.4	407.3	6.0	1.3	407.3	6.0
FZ3 094	71	54	1.3	0.05974	0.00314	0.53738	0.02618	0.06524	0.00158	594.2	110.2	436.7	17.3	407.4	9.6	6.7	407.4	9.6
FZ3 092	176	277	0.6	0.05684	0.00122	0.51496	0.01018	0.06571	0.00091	484.8	46.9	421.8	6.8	410.3	5.5	2.7	410.3	5.5
FZ3 077	64	476	0.1	0.05853	0.00098	0.53482	0.00825	0.06627	0.00083	549.8	36.1	435.0	5.5	413.7	5.0	4.9	413.7	5.0
FZ3 068	101	112	0.9	0.05447	0.00181	0.50218	0.01541	0.06687	0.00117	390.5	72.2	413.2	10.4	417.3	7.1	-1.0	417.3	7.1
FZ3 089	109	327	0.3	0.05540	0.00115	0.51436	0.00983	0.06735	0.00091	428.2	45.0	421.4	6.6	420.1	5.5	0.3	420.1	5.5
FZ3 003	138	207	0.7	0.05546	0.00132	0.51678	0.01139	0.06758	0.00098	430.7	51.8	423.0	7.6	421.6	5.9	0.3	421.6	5.9
FZ3 087	194	273	0.7	0.05605	0.00127	0.52224	0.01090	0.06758	0.00096	454.1	49.3	426.6	7.3	421.6	5.8	1.2	421.6	5.8
FZ3 088	154	227	0.7	0.05608	0.00122	0.52244	0.01050	0.06758	0.00094	455.0	47.4	426.8	7.0	421.6	5.7	1.2	421.6	5.7
FZ3 078	124	271	0.5	0.05552	0.00113	0.51797	0.00976	0.06767	0.00091	432.9	44.3	423.8	6.5	422.1	5.5	0.4	422.1	5.5
FZ3 009	62	258	0.2	0.05612	0.00113	0.52434	0.00973	0.06777	0.00091	456.6	43.8	428.0	6.5	422.7	5.5	1.2	422.7	5.5
FZ3 047	87	206	0.4	0.05578	0.00145	0.52721	0.01267	0.06855	0.00105	443.3	56.8	430.0	8.4	427.4	6.3	0.6	427.4	6.3
FZ3 093	234	427	0.5	0.05500	0.00091	0.52220	0.00804	0.06886	0.00085	412.4	36.3	426.6	5.4	429.3	5.1	-0.6	429.3	5.1
FZ3 051	214	269	0.8	0.05630	0.00117	0.53756	0.01030	0.06926	0.00094	463.4	45.7	436.8	6.8	431.7	5.7	1.2	431.7	5.7
FZ3 057	117	187	0.6	0.05725	0.00162	0.54717	0.01430	0.06932	0.00111	500.8	61.7	443.1	9.4	432.1	6.7	2.5	432.1	6.7
FZ3 021	236	308	0.8	0.05494	0.00103	0.52934	0.00924	0.06989	0.00092	409.5	41.3	431.4	6.1	435.5	5.5	-1.0	435.5	5.5
FZ3 050	199	242	0.8	0.05546	0.00120	0.53485	0.01074	0.06995	0.00097	430.6	47.2	435.0	7.1	435.8	5.9	-0.2	435.8	5.9
FZ3 022	143	292	0.5	0.05654	0.00107	0.54647	0.00960	0.07010	0.00092	472.9	41.9	442.7	6.3	436.8	5.6	1.3	436.8	5.6
FZ3 059	159	288	0.6	0.05740	0.00112	0.55674	0.00999	0.07035	0.00094	506.7	42.6	449.4	6.5	438.2	5.6	2.5	438.2	5.6
FZ3 042	97	220	0.4	0.05636	0.00131	0.54665	0.01171	0.07035	0.00101	465.8	51.1	442.8	7.7	438.3	6.1	1.0	438.3	6.1
FZ3 004	100	259	0.4	0.05551	0.00108	0.54007	0.00975	0.07057	0.00094	432.4	42.4	438.5	6.4	439.6	5.7	-0.3	439.6	5.7
FZ3 044	129	191	0.7	0.05627	0.00135	0.54927	0.01218	0.07080	0.00103	462.4	52.8	444.5	8.0	440.9	6.2	0.8	440.9	6.2
FZ3 070	232	316	0.7	0.05691	0.00139	0.55611	0.01250	0.07088	0.00105	487.5	53.0	449.0	8.2	441.4	6.3	1.7	441.4	6.3
FZ3 032	119	270	0.4	0.05681	0.00126	0.55895	0.01147	0.07136	0.00101	483.5	48.6	450.8	7.5	444.4	6.1	1.4	444.4	6.1
FZ3 010	75	143	0.5	0.05788	0.00163	0.57503	0.01497	0.07206	0.00115	524.8	60.9	461.3	9.7	448.6	6.9	2.8	448.6	6.9
FZ3 049	98	207	0.5	0.05946	0.00148	0.61344	0.01399	0.07483	0.00113	583.9	53.0	485.7	8.8	465.2	6.8	4.2	465.2	6.8
FZ3 025	152	329	0.5	0.05933	0.00122	0.62001	0.01174	0.07580	0.00105	579.1	44.1	489.9	7.4	471.0	6.3	3.9	471.0	6.3
FZ3 023	8	28	0.3	0.05924	0.00569	0.63378	0.05702	0.07760	0.00301	575.8	196.0	498.5	35.4	481.8	18.0	3.4	481.8	18.0
FZ3 083	87	352	0.2	0.06660	0.00105	0.71464	0.01040	0.07783	0.00098	825.4	32.7	547.5	6.2	483.2	5.8	11.7	483.2	5.8

FZ3 033	32	118	0.3	0.05729	0.00259	0.62002	0.02618	0.07850	0.00163	502.2	97.0	489.9	16.4	487.2	9.8	0.6	487.2	9.8
FZ3 017	76	136	0.6	0.05754	0.00152	0.63686	0.01558	0.08028	0.00124	512.0	57.5	500.4	9.7	497.8	7.4	0.5	497.8	7.4
FZ3 074	254	570	0.4	0.06261	0.00081	0.85495	0.01029	0.09904	0.00117	695.2	27.4	627.4	5.6	608.8	6.9	3.0	608.8	6.9
FZ3 029	66	48	1.4	0.06309	0.00225	1.00270	0.03303	0.11527	0.00220	711.3	73.9	705.2	16.8	703.3	12.7	0.3	703.3	12.7
FZ3 061	111	476	0.2	0.06531	0.00089	1.12724	0.01424	0.12519	0.00151	784.4	28.3	766.4	6.8	760.4	8.7	0.8	760.4	8.7
FZ3 091	69	136	0.5	0.06744	0.00188	1.18640	0.03035	0.12762	0.00218	851.2	57.0	794.3	14.1	774.2	12.4	2.5	774.2	12.4
FZ3 058	35	94	0.4	0.07213	0.00192	1.50996	0.03673	0.15185	0.00257	989.5	53.1	934.4	14.9	911.3	14.4	2.5	911.3	14.4
FZ3 081	78	221	0.4	0.08540	0.00113	1.86586	0.02267	0.15847	0.00196	1324.7	25.5	1069.1	8.0	948.3	10.9	11.3	948.3	10.9
FZ3 064	87	111	0.8	0.11748	0.00146	5.40023	0.06271	0.33343	0.00433	1918.2	22.1	1884.9	10.0	1855.0	20.9	3.3	1918.2	22.1
FZ3 075	321	148	2.2	0.05316	0.00235	0.27351	0.01125	0.03732	0.00075	335.4	96.9	245.5	9.0	236.2	4.6	3.8	236.2	4.6
FZ3 072	269	351	0.8	0.06391	0.00221	0.33392	0.01043	0.03790	0.00071	738.6	71.6	292.6	7.9	239.8	4.4	18.0	239.8	4.4
FZ3 026	169	213	0.8	0.05261	0.00174	0.27599	0.00845	0.03805	0.00064	312.1	73.5	247.5	6.7	240.7	4.0	2.7	240.7	4.0
FZ3 090	190	153	1.2	0.05747	0.00332	0.30623	0.01613	0.03865	0.00104	509.2	122.4	271.3	12.5	244.5	6.5	9.9	244.5	6.5
FZ3 056	103	82	1.3	0.05213	0.00316	0.28482	0.01605	0.03963	0.00103	291.3	132.8	254.5	12.7	250.5	6.4	1.6	250.5	6.4
FZ3 041	103	135	0.8	0.05003	0.00212	0.27532	0.01088	0.03991	0.00076	196.4	95.4	246.9	8.7	252.3	4.7	-2.2	252.3	4.7
~~FZ3 030~~	~~93~~	~~63~~	~~1.5~~	~~0.07310~~	~~0.00436~~	~~0.40373~~	~~0.02192~~	~~0.04006~~	~~0.00113~~	~~1016.6~~	~~116.3~~	~~344.3~~	~~15.9~~	~~253.2~~	~~7.0~~	~~26.5~~	~~253.2~~	~~7.0~~
FZ3 039	767	292	2.6	0.05239	0.00196	0.29121	0.01006	0.04032	0.00075	302.4	83.2	259.5	7.9	254.8	4.7	1.8	254.8	4.7
FZ3 054	83	102	0.8	0.05350	0.00265	0.29784	0.01377	0.04038	0.00088	349.9	108.1	264.7	10.8	255.2	5.4	3.6	255.2	5.4
FZ3 002	42	167	0.3	0.05128	0.00170	0.28621	0.00877	0.04048	0.00069	253.4	74.4	255.6	6.9	255.8	4.3	-0.1	255.8	4.3
FZ3 067	126	74	1.7	0.05260	0.00370	0.29503	0.01947	0.04068	0.00113	311.7	151.9	262.5	15.3	257.1	7.0	2.1	257.1	7.0
FZ3 055	99	123	0.8	0.05350	0.00322	0.30064	0.01668	0.04076	0.00109	349.9	129.9	266.9	13.0	257.6	6.8	3.5	257.6	6.8
FZ3 016	344	274	1.3	0.05203	0.00137	0.29336	0.00715	0.04089	0.00061	286.7	59.2	261.2	5.6	258.4	3.8	1.1	258.4	3.8
FZ3 011	58	40	1.5	0.05205	0.00494	0.29349	0.02653	0.04090	0.00134	287.4	203.1	261.3	20.8	258.4	8.3	1.1	258.4	8.3
FZ3 085	86	250	0.3	0.05216	0.00178	0.29684	0.00934	0.04128	0.00072	292.3	76.0	263.9	7.3	260.8	4.5	1.2	260.8	4.5
FZ3 065	416	174	2.4	0.05619	0.00243	0.31986	0.01272	0.04129	0.00086	459.0	93.9	281.8	9.8	260.8	5.3	7.5	260.8	5.3
FZ3 015	145	107	1.4	0.05008	0.00225	0.28544	0.01194	0.04134	0.00083	198.9	101.0	255.0	9.4	261.1	5.2	-2.4	261.1	5.2
FZ3 024	83	90	0.9	0.05456	0.00348	0.31245	0.01849	0.04153	0.00113	394.4	136.6	276.1	14.3	262.3	7.0	5.0	262.3	7.0
FZ3 066	281	542	0.5	0.05607	0.00149	0.32105	0.00782	0.04153	0.00063	454.7	57.8	282.7	6.0	262.3	3.9	7.2	262.3	3.9
FZ3 082	370	327	1.1	0.05213	0.00142	0.29972	0.00755	0.04171	0.00063	290.9	60.9	266.2	5.9	263.4	3.9	1.1	263.4	3.9
FZ3 053	159	281	0.6	0.05248	0.00146	0.30185	0.00779	0.04172	0.00064	306.5	62.1	267.8	6.1	263.5	4.0	1.6	263.5	4.0
FZ3 006	69	93	0.7	0.05327	0.00336	0.30942	0.01812	0.04213	0.00113	340.1	136.1	273.7	14.1	266.0	7.0	2.8	266.0	7.0

FZ3 079	203	221	0.9	0.05956	0.00190	0.34630	0.01013	0.04218	0.00073	587.5	67.9	301.9	7.6	266.3	4.5	11.8	266.3	4.5
FZ3 014	301	285	1.1	0.05290	0.00133	0.30792	0.00717	0.04222	0.00062	324.3	56.1	272.6	5.6	266.6	3.8	2.2	266.6	3.8
FZ3 013	163	267	0.6	0.05018	0.00129	0.29327	0.00700	0.04239	0.00062	203.5	58.6	261.1	5.5	267.6	3.8	-2.5	267.6	3.8
FZ3 084	158	171	0.9	0.05190	0.00186	0.30409	0.01009	0.04250	0.00075	281.0	79.8	269.6	7.9	268.3	4.7	0.5	268.3	4.7
FZ3 005	156	208	0.7	0.05441	0.00213	0.32102	0.01155	0.04280	0.00084	387.8	85.1	282.7	8.9	270.1	5.2	4.5	270.1	5.2
FZ3 076	139	294	0.5	0.05286	0.00140	0.31215	0.00765	0.04284	0.00064	322.6	59.1	275.8	5.9	270.4	4.0	2.0	270.4	4.0
FZ3 037	116	124	0.9	0.05135	0.00216	0.30353	0.01194	0.04288	0.00081	256.4	93.8	269.2	9.3	270.6	5.0	-0.5	270.6	5.0
FZ3 060	17	26	0.6	0.05490	0.00852	0.32462	0.04739	0.04289	0.00244	407.9	314.3	285.5	36.3	270.7	15.1	5.2	270.7	15.1
FZ3 097	41	39	1.0	0.05360	0.00510	0.31697	0.02849	0.04290	0.00148	354.0	201.7	279.6	22.0	270.8	9.2	3.1	270.8	9.2
FZ3 045	100	180	0.6	0.05333	0.00185	0.31686	0.01014	0.04309	0.00076	343.0	76.5	279.5	7.8	272.0	4.7	2.7	272.0	4.7
FZ3 071	72	131	0.5	0.05113	0.00230	0.30491	0.01278	0.04326	0.00089	246.7	100.5	270.2	9.9	273.0	5.5	-1.0	273.0	5.5
FZ3 040	270	284	1.0	0.05172	0.00131	0.30846	0.00721	0.04326	0.00063	273.1	56.8	273.0	5.6	273.0	3.9	0.0	273.0	3.9
FZ3 008	204	348	0.6	0.05630	0.00184	0.33604	0.01009	0.04329	0.00076	463.5	71.2	294.2	7.7	273.2	4.7	7.1	273.2	4.7
~~FZ3 018~~	~~55~~	~~96~~	~~0.6~~	~~0.07760~~	~~0.00374~~	~~0.46309~~	~~0.01988~~	~~0.04329~~	~~0.00111~~	~~1136.6~~	~~93.0~~	~~386.4~~	~~13.8~~	~~273.2~~	~~6.8~~	~~29.3~~	~~273.2~~	~~6.8~~
FZ3 046	57	91	0.6	0.05315	0.00294	0.31730	0.01634	0.04330	0.00103	335.2	120.3	279.8	12.6	273.3	6.4	2.3	273.3	6.4
FZ3 007	45	44	1.0	0.05646	0.00446	0.33731	0.02482	0.04333	0.00141	469.7	166.8	295.1	18.8	273.5	8.7	7.3	273.5	8.7
FZ3 027	153	176	0.9	0.05271	0.00172	0.31602	0.00955	0.04348	0.00072	316.4	72.4	278.8	7.4	274.4	4.5	1.6	274.4	4.5
FZ3 043	129	161	0.8	0.05164	0.00182	0.31047	0.01019	0.04361	0.00074	269.6	78.6	274.5	7.9	275.1	4.6	-0.2	275.1	4.6
FZ3 080	258	406	0.6	0.05776	0.00176	0.34715	0.00966	0.04360	0.00073	520.4	65.7	302.6	7.3	275.1	4.5	9.1	275.1	4.5
FZ3 062	131	258	0.5	0.05836	0.00229	0.35161	0.01268	0.04370	0.00086	543.2	83.7	305.9	9.5	275.7	5.3	9.9	275.7	5.3
FZ3 098	69	114	0.6	0.06715	0.00242	0.40736	0.01342	0.04401	0.00083	842.3	73.4	347.0	9.7	277.6	5.1	20.0	277.6	5.1
FZ3 063	47	57	0.8	0.05020	0.00387	0.30470	0.02233	0.04403	0.00122	204.1	169.8	270.1	17.4	277.8	7.6	-2.9	277.8	7.6
FZ3 099	85	129	0.7	0.05241	0.00254	0.31919	0.01431	0.04418	0.00098	303.2	106.8	281.3	11.0	278.7	6.0	0.9	278.7	6.0
FZ3 038	202	141	1.4	0.05165	0.00196	0.31565	0.01120	0.04433	0.00080	269.8	84.9	278.6	8.6	279.6	4.9	-0.4	279.6	4.9
FZ3 036	157	241	0.7	0.05546	0.00161	0.33929	0.00905	0.04437	0.00071	430.5	63.1	296.6	6.9	279.9	4.4	5.6	279.9	4.4
FZ3 031	175	269	0.7	0.05252	0.00135	0.32240	0.00767	0.04452	0.00066	308.2	57.5	283.7	5.9	280.8	4.1	1.0	280.8	4.1
FZ3 096	23	33	0.7	0.05382	0.00722	0.33054	0.04135	0.04455	0.00234	363.5	277.1	290.0	31.6	281.0	14.4	3.1	281.0	14.4
FZ3 028	66	163	0.4	0.05573	0.00186	0.34239	0.01053	0.04456	0.00078	441.2	72.6	299.0	8.0	281.0	4.8	6.0	281.0	4.8
FZ3 095	84	154	0.5	0.05365	0.00251	0.33222	0.01434	0.04492	0.00098	356.3	102.0	291.3	10.9	283.2	6.1	2.8	283.2	6.1
FZ3 052	138	258	0.5	0.05290	0.00144	0.32984	0.00830	0.04523	0.00068	324.4	60.4	289.4	6.3	285.1	4.2	1.5	285.1	4.2
FZ3 020	141	223	0.6	0.05390	0.00208	0.33804	0.01206	0.04549	0.00087	366.6	84.7	295.7	9.2	286.8	5.4	3.0	286.8	5.4

FZ3 012	83	82	1.0	0.05262	0.00322	0.33177	0.01884	0.04573	0.00121	312.3	133.2	290.9	14.4	288.3	7.5	0.9	288.3	7.5
FZ3 035	98	127	0.8	0.05162	0.00196	0.32717	0.01155	0.04597	0.00084	268.6	84.8	287.4	8.8	289.7	5.2	-0.8	289.7	5.2
FZ3 001	129	109	1.2	0.05255	0.00224	0.34344	0.01361	0.04740	0.00094	309.5	94.3	299.8	10.3	298.5	5.8	0.4	298.5	5.8
FZ3 034	109	125	0.9	0.05325	0.00383	0.34901	0.02321	0.04754	0.00149	339.3	154.7	304.0	17.5	299.4	9.1	1.5	299.4	9.1
FZ3 073	175	153	1.1	0.05408	0.00232	0.35447	0.01402	0.04755	0.00098	374.1	93.1	308.1	10.5	299.4	6.0	2.8	299.4	6.0
FZ3 069	86	92	0.9	0.05830	0.00351	0.39859	0.02222	0.04959	0.00133	540.2	127.3	340.6	16.1	312.0	8.2	8.4	312.0	8.2
FZ3 019	128	328	0.4	0.05681	0.00151	0.41222	0.01007	0.05263	0.00082	483.4	57.8	350.5	7.2	330.7	5.0	5.6	330.7	5.0
FZ3 086	131	1199	0.1	0.05608	0.00067	0.46955	0.00527	0.06073	0.00069	455.3	26.1	390.9	3.6	380.1	4.2	2.8	380.1	4.2
FZ3 100	62	256	0.2	0.05548	0.00118	0.49255	0.00966	0.06440	0.00088	431.2	46.2	406.6	6.6	402.3	5.3	1.1	402.3	5.3
FZ3 048	34	180	0.2	0.05576	0.00149	0.50144	0.01243	0.06522	0.00100	442.6	58.3	412.7	8.4	407.3	6.0	1.3	407.3	6.0
FZ3 094	71	54	1.3	0.05974	0.00314	0.53738	0.02618	0.06524	0.00158	594.2	110.2	436.7	17.3	407.4	9.6	6.7	407.4	9.6
FZ3 092	176	277	0.6	0.05684	0.00122	0.51496	0.01018	0.06571	0.00091	484.8	46.9	421.8	6.8	410.3	5.5	2.7	410.3	5.5
FZ3 077	64	476	0.1	0.05853	0.00098	0.53482	0.00825	0.06627	0.00083	549.8	36.1	435.0	5.5	413.7	5.0	4.9	413.7	5.0
FZ3 068	101	112	0.9	0.05447	0.00181	0.50218	0.01541	0.06687	0.00117	390.5	72.2	413.2	10.4	417.3	7.1	-1.0	417.3	7.1
FZ3 089	109	327	0.3	0.05540	0.00115	0.51436	0.00983	0.06735	0.00091	428.2	45.0	421.4	6.6	420.1	5.5	0.3	420.1	5.5
FZ3 003	138	207	0.7	0.05546	0.00132	0.51678	0.01139	0.06758	0.00098	430.7	51.8	423.0	7.6	421.6	5.9	0.3	421.6	5.9
FZ3 087	194	273	0.7	0.05605	0.00127	0.52224	0.01090	0.06758	0.00096	454.1	49.3	426.6	7.3	421.6	5.8	1.2	421.6	5.8
FZ3 088	154	227	0.7	0.05608	0.00122	0.52244	0.01050	0.06758	0.00094	455.0	47.4	426.8	7.0	421.6	5.7	1.2	421.6	5.7
FZ3 078	124	271	0.5	0.05552	0.00113	0.51797	0.00976	0.06767	0.00091	432.9	44.3	423.8	6.5	422.1	5.5	0.4	422.1	5.5
FZ3 009	62	258	0.2	0.05612	0.00113	0.52434	0.00973	0.06777	0.00091	456.6	43.8	428.0	6.5	422.7	5.5	1.2	422.7	5.5
FZ3 047	87	206	0.4	0.05578	0.00145	0.52721	0.01267	0.06855	0.00105	443.3	56.8	430.0	8.4	427.4	6.3	0.6	427.4	6.3
FZ3 093	234	427	0.5	0.05500	0.00091	0.52220	0.00804	0.06886	0.00085	412.4	36.3	426.6	5.4	429.3	5.1	-0.6	429.3	5.1
FZ3 051	214	269	0.8	0.05630	0.00117	0.53756	0.01030	0.06926	0.00094	463.4	45.7	436.8	6.8	431.7	5.7	1.2	431.7	5.7
FZ3 057	117	187	0.6	0.05725	0.00162	0.54717	0.01430	0.06932	0.00111	500.8	61.7	443.1	9.4	432.1	6.7	2.5	432.1	6.7
FZ3 021	236	308	0.8	0.05494	0.00103	0.52934	0.00924	0.06989	0.00092	409.5	41.3	431.4	6.1	435.5	5.5	-1.0	435.5	5.5
FZ3 050	199	242	0.8	0.05546	0.00120	0.53485	0.01074	0.06995	0.00097	430.6	47.2	435.0	7.1	435.8	5.9	-0.2	435.8	5.9
FZ3 022	143	292	0.5	0.05654	0.00107	0.54647	0.00960	0.07010	0.00092	472.9	41.9	442.7	6.3	436.8	5.6	1.3	436.8	5.6
FZ3 059	159	288	0.6	0.05740	0.00112	0.55674	0.00999	0.07035	0.00094	506.7	42.6	449.4	6.5	438.2	5.6	2.5	438.2	5.6
FZ3 042	97	220	0.4	0.05636	0.00131	0.54665	0.01171	0.07035	0.00101	465.8	51.1	442.8	7.7	438.3	6.1	1.0	438.3	6.1
FZ3 004	100	259	0.4	0.05551	0.00108	0.54007	0.00975	0.07057	0.00094	432.4	42.4	438.5	6.4	439.6	5.7	-0.3	439.6	5.7
FZ3 044	129	191	0.7	0.05627	0.00135	0.54927	0.01218	0.07080	0.00103	462.4	52.8	444.5	8.0	440.9	6.2	0.8	440.9	6.2

FZ3 070	232	316	0.7	0.05691	0.00139	0.55611	0.01250	0.07088	0.00105	487.5	53.0	449.0	8.2	441.4	6.3	1.7	441.4	6.3
FZ3 032	119	270	0.4	0.05681	0.00126	0.55895	0.01147	0.07136	0.00101	483.5	48.6	450.8	7.5	444.4	6.1	1.4	444.4	6.1
FZ3 010	75	143	0.5	0.05788	0.00163	0.57503	0.01497	0.07206	0.00115	524.8	60.9	461.3	9.7	448.6	6.9	2.8	448.6	6.9
FZ3 049	98	207	0.5	0.05946	0.00148	0.61344	0.01399	0.07483	0.00113	583.9	53.0	485.7	8.8	465.2	6.8	4.2	465.2	6.8
FZ3 025	152	329	0.5	0.05933	0.00122	0.62001	0.01174	0.07580	0.00105	579.1	44.1	489.9	7.4	471.0	6.3	3.9	471.0	6.3
FZ3 023	8	28	0.3	0.05924	0.00569	0.63378	0.05702	0.07760	0.00301	575.8	196.0	498.5	35.4	481.8	18.0	3.4	481.8	18.0
FZ3 083	87	352	0.2	0.06660	0.00105	0.71464	0.01040	0.07783	0.00098	825.4	32.7	547.5	6.2	483.2	5.8	11.7	483.2	5.8
FZ3 033	32	118	0.3	0.05729	0.00259	0.62002	0.02618	0.07850	0.00163	502.2	97.0	489.9	16.4	487.2	9.8	0.6	487.2	9.8
FZ3 017	76	136	0.6	0.05754	0.00152	0.63686	0.01558	0.08028	0.00124	512.0	57.5	500.4	9.7	497.8	7.4	0.5	497.8	7.4
FZ3 074	254	570	0.4	0.06261	0.00081	0.85495	0.01029	0.09904	0.00117	695.2	27.4	627.4	5.6	608.8	6.9	3.0	608.8	6.9
FZ3 029	66	48	1.4	0.06309	0.00225	1.00270	0.03303	0.11527	0.00220	711.3	73.9	705.2	16.8	703.3	12.7	0.3	703.3	12.7
FZ3 061	111	476	0.2	0.06531	0.00089	1.12724	0.01424	0.12519	0.00151	784.4	28.3	766.4	6.8	760.4	8.7	0.8	760.4	8.7
FZ3 091	69	136	0.5	0.06744	0.00188	1.18640	0.03035	0.12762	0.00218	851.2	57.0	794.3	14.1	774.2	12.4	2.5	774.2	12.4
FZ3 058	35	94	0.4	0.07213	0.00192	1.50996	0.03673	0.15185	0.00257	989.5	53.1	934.4	14.9	911.3	14.4	2.5	911.3	14.4
FZ3 081	78	221	0.4	0.08540	0.00113	1.86586	0.02267	0.15847	0.00196	1324.7	25.5	1069.1	8.0	948.3	10.9	11.3	948.3	10.9
FZ3 064	87	111	0.8	0.11748	0.00146	5.40023	0.06271	0.33343	0.00433	1918.2	22.1	1884.9	10.0	1855.0	20.9	3.3	1918.2	22.1
FZ3 075	321	148	2.2	0.05316	0.00235	0.27351	0.01125	0.03732	0.00075	335.4	96.9	245.5	9.0	236.2	4.6	3.8	236.2	4.6
FZ3 072	269	351	0.8	0.06391	0.00221	0.33392	0.01043	0.03790	0.00071	738.6	71.6	292.6	7.9	239.8	4.4	18.0	239.8	4.4
FZ4 036	315	200	1.6	0.05204	0.00179	0.28130	0.00890	0.03921	0.00069	287.1	76.5	251.7	7.1	247.9	4.3	1.5	247.9	4.3
FZ4 037	232	242	1.0	0.05114	0.00155	0.28363	0.00800	0.04023	0.00064	247.0	68.4	253.5	6.3	254.3	4.0	-0.3	254.3	4.0
FZ4 006	342	190	1.8	0.05071	0.00160	0.28721	0.00836	0.04108	0.00067	227.9	71.1	256.4	6.6	259.5	4.2	-1.2	259.5	4.2
FZ4 090	239	263	0.9	0.05416	0.00180	0.30780	0.00944	0.04122	0.00071	377.5	72.9	272.5	7.3	260.4	4.4	4.4	260.4	4.4
FZ4 011	185	327	0.6	0.05092	0.00119	0.28945	0.00624	0.04124	0.00058	237.0	52.9	258.1	4.9	260.5	3.6	-0.9	260.5	3.6
FZ4 008	88	172	0.5	0.05455	0.00179	0.31040	0.00943	0.04127	0.00070	393.9	71.3	274.5	7.3	260.7	4.3	5.0	260.7	4.3
FZ4 091	79	89	0.9	0.05392	0.00437	0.30970	0.02318	0.04166	0.00144	367.4	172.4	274.0	18.0	263.1	8.9	4.0	263.1	8.9
FZ4 072	164	305	0.5	0.05384	0.00138	0.31189	0.00741	0.04202	0.00062	364.1	56.8	275.6	5.7	265.3	3.8	3.7	265.3	3.8
FZ4 050	120	147	0.8	0.05146	0.00203	0.29840	0.01092	0.04206	0.00079	261.7	88.0	265.2	8.5	265.6	4.9	-0.2	265.6	4.9
FZ4 009	111	132	0.8	0.05178	0.00198	0.30280	0.01079	0.04242	0.00077	275.8	85.3	268.6	8.4	267.8	4.8	0.3	267.8	4.8
FZ4 094	89	106	0.8	0.05129	0.00259	0.30192	0.01424	0.04269	0.00094	254.0	112.1	267.9	11.1	269.5	5.8	-0.6	269.5	5.8
FZ4 059	186	300	0.6	0.05239	0.00139	0.31140	0.00763	0.04311	0.00064	302.4	59.1	275.3	5.9	272.1	4.0	1.2	272.1	4.0

FZ4 077	109	154	0.7	0.05147	0.00188	0.30804	0.01046	0.04341	0.00078	262.0	81.9	272.7	8.1	273.9	4.8	-0.4	273.9	4.8
FZ4 060	88	154	0.6	0.05305	0.00209	0.31907	0.01168	0.04363	0.00081	330.9	86.8	281.2	9.0	275.3	5.0	2.1	275.3	5.0
FZ4 027	114	173	0.7	0.05517	0.00175	0.33555	0.00981	0.04412	0.00073	419.0	68.7	293.8	7.5	278.3	4.5	5.3	278.3	4.5
~~FZ4 030~~	~~32~~	~~49~~	~~0.7~~	~~0.06785~~	~~0.00441~~	~~0.41260~~	~~0.02486~~	~~0.04411~~	~~0.00124~~	~~863.8~~	~~129.4~~	~~350.7~~	~~17.9~~	~~278.3~~	~~7.6~~	~~20.6~~	~~278.3~~	~~7.6~~
FZ4 087	109	156	0.7	0.05343	0.00205	0.32791	0.01169	0.04451	0.00082	347.2	84.5	288.0	8.9	280.7	5.1	2.5	280.7	5.1
FZ4 033	103	165	0.6	0.05477	0.00269	0.33790	0.01527	0.04475	0.00103	403.0	106.1	295.6	11.6	282.2	6.4	4.5	282.2	6.4
FZ4 079	113	192	0.6	0.05599	0.00200	0.34801	0.01142	0.04508	0.00083	451.8	77.4	303.2	8.6	284.2	5.1	6.3	284.2	5.1
FZ4 097	40	81	0.5	0.05175	0.00284	0.32246	0.01653	0.04519	0.00106	274.5	121.1	283.8	12.7	284.9	6.5	-0.4	284.9	6.5
FZ4 010	150	148	1.0	0.05441	0.00181	0.34266	0.01049	0.04568	0.00079	388.0	72.2	299.2	7.9	288.0	4.9	3.7	288.0	4.9
FZ4 045	149	107	1.4	0.05595	0.00330	0.35293	0.01914	0.04576	0.00123	449.9	126.2	306.9	14.4	288.4	7.6	6.0	288.4	7.6
FZ4 035	72	210	0.3	0.05135	0.00146	0.33254	0.00880	0.04698	0.00073	256.5	64.3	291.5	6.7	295.9	4.5	-1.5	295.9	4.5
FZ4 032	84	120	0.7	0.05194	0.00327	0.35090	0.02046	0.04900	0.00135	282.9	137.9	305.4	15.4	308.4	8.3	-1.0	308.4	8.3
FZ4 063	365	358	1.0	0.05423	0.00116	0.38185	0.00757	0.05108	0.00070	380.3	47.4	328.4	5.6	321.1	4.3	2.2	321.1	4.3
FZ4 013	164	271	0.6	0.05537	0.00122	0.44081	0.00896	0.05775	0.00080	426.9	47.9	370.8	6.3	361.9	4.9	2.4	361.9	4.9
FZ4 048	23	43	0.5	0.05162	0.00338	0.41164	0.02547	0.05785	0.00148	268.4	143.6	350.1	18.3	362.5	9.0	-3.5	362.5	9.0
FZ4 074	143	217	0.7	0.05352	0.00151	0.49133	0.01287	0.06658	0.00105	351.0	62.7	405.8	8.8	415.5	6.3	-2.4	415.5	6.3
FZ4 031	66	231	0.3	0.05439	0.00122	0.50555	0.01048	0.06742	0.00095	387.4	49.3	415.5	7.1	420.6	5.7	-1.2	420.6	5.7
FZ4 014	289	455	0.6	0.05531	0.00088	0.51464	0.00757	0.06749	0.00083	424.7	34.4	421.6	5.1	421.0	5.0	0.1	421.0	5.0
FZ4 067	82	614	0.1	0.06042	0.00149	0.56691	0.01277	0.06806	0.00103	618.5	52.3	456.0	8.3	424.5	6.2	6.9	424.5	6.2
FZ4 020	42	88	0.5	0.05578	0.00197	0.53078	0.01731	0.06902	0.00125	443.3	76.7	432.3	11.5	430.3	7.6	0.5	430.3	7.6
FZ4 028	44	175	0.3	0.05261	0.00177	0.50391	0.01563	0.06948	0.00123	312.0	74.6	414.3	10.6	433.0	7.4	-4.5	433.0	7.4
FZ4 026	151	288	0.5	0.05529	0.00108	0.53026	0.00954	0.06957	0.00092	423.7	42.2	432.0	6.3	433.6	5.6	-0.4	433.6	5.6
FZ4 051	90	195	0.5	0.05776	0.00143	0.55447	0.01269	0.06963	0.00104	520.4	53.8	447.9	8.3	433.9	6.3	3.1	433.9	6.3
FZ4 004	217	283	0.8	0.05842	0.00122	0.56644	0.01089	0.07034	0.00097	545.4	45.0	455.7	7.1	438.2	5.8	3.8	438.2	5.8
FZ4 053	215	331	0.7	0.05545	0.00105	0.54333	0.00955	0.07107	0.00093	430.3	41.2	440.6	6.3	442.6	5.6	-0.5	442.6	5.6
FZ4 015	215	372	0.6	0.05452	0.00092	0.53966	0.00849	0.07180	0.00090	392.7	37.4	438.2	5.6	447.0	5.4	-2.0	447.0	5.4
FZ4 056	3	31	0.1	0.05358	0.00382	0.53167	0.03567	0.07198	0.00205	353.2	153.0	432.9	23.6	448.1	12.3	-3.5	448.1	12.3
FZ4 071	166	334	0.5	0.05707	0.00104	0.57443	0.00972	0.07301	0.00095	493.5	39.9	460.9	6.3	454.3	5.7	1.4	454.3	5.7
FZ4 061	76	72	1.1	0.06049	0.00236	0.61516	0.02207	0.07377	0.00147	621.0	81.9	486.8	13.9	458.8	8.8	5.8	458.8	8.8
FZ4 021	109	147	0.7	0.05532	0.00147	0.56342	0.01387	0.07388	0.00113	425.1	57.7	453.8	9.0	459.5	6.8	-1.3	459.5	6.8
FZ4 083	81	102	0.8	0.05586	0.00189	0.59209	0.01865	0.07688	0.00134	446.5	73.7	472.2	11.9	477.5	8.0	-1.1	477.5	8.0

FZ4 065	5	536	0.0	0.05606	0.00081	0.59481	0.00804	0.07696	0.00093	454.3	31.6	473.9	5.1	478.0	5.5	-0.9	478.0	5.5
FZ4 064	265	483	0.5	0.05949	0.00103	0.63527	0.01018	0.07745	0.00100	585.2	37.3	499.4	6.3	480.9	6.0	3.7	480.9	6.0
FZ4 076	70	236	0.3	0.05894	0.00151	0.64577	0.01515	0.07947	0.00123	564.9	54.7	505.9	9.4	492.9	7.3	2.6	492.9	7.3
FZ4 018	229	309	0.7	0.05775	0.00097	0.63315	0.00984	0.07952	0.00101	520.2	36.7	498.1	6.1	493.3	6.0	1.0	493.3	6.0
FZ4 068	217	394	0.6	0.05738	0.00092	0.63533	0.00948	0.08031	0.00100	505.7	34.8	499.4	5.9	498.0	6.0	0.3	498.0	6.0
FZ4 005	58	81	0.7	0.06016	0.00202	0.67642	0.02102	0.08155	0.00146	609.4	71.1	524.6	12.7	505.4	8.7	3.7	505.4	8.7
FZ4 098	65	49	1.3	0.06618	0.00234	1.14203	0.03705	0.12516	0.00245	812.1	72.1	773.5	17.6	760.2	14.1	1.7	760.2	14.1
FZ4 002	133	232	0.6	0.06632	0.00097	1.15168	0.01559	0.12597	0.00156	816.4	30.3	778.0	7.4	764.8	8.9	1.7	764.8	8.9
FZ4 086	32	68	0.5	0.06743	0.00195	1.19710	0.03172	0.12878	0.00224	850.9	58.9	799.2	14.7	780.9	12.8	2.3	780.9	12.8
FZ4 017	75	314	0.2	0.06789	0.00080	1.43896	0.01587	0.15375	0.00180	865.1	24.3	905.3	6.6	922.0	10.1	-1.8	922.0	10.1
FZ4 025	126	182	0.7	0.08833	0.00105	2.69810	0.02974	0.22157	0.00269	1389.7	22.6	1327.9	8.2	1290.2	14.2	7.2	1389.7	22.6
FZ4 088	20	33	0.6	0.09457	0.00443	3.39174	0.14548	0.26013	0.00776	1519.6	85.8	1502.5	33.6	1490.5	39.7	1.9	1519.6	85.8
FZ4 062	8	22	0.4	0.09882	0.00578	3.31132	0.17579	0.24306	0.00894	1601.9	105.3	1483.7	41.4	1402.6	46.4	12.4	1601.9	105
FZ4 075	117	182	0.6	0.09955	0.00110	3.78814	0.03921	0.27601	0.00333	1615.7	20.4	1590.2	8.3	1571.2	16.8	2.8	1615.7	20.4
FZ4 024	40	47	0.8	0.10182	0.00182	4.19167	0.06920	0.29862	0.00451	1657.6	32.7	1672.4	13.5	1684.5	22.4	-1.6	1657.6	32.7
~~FZ4 089~~	~~18~~	~~32~~	~~0.6~~	~~0.10312~~	~~0.00299~~	~~2.97839~~	~~0.07799~~	~~0.20950~~	~~0.00421~~	~~1680.9~~	~~52.7~~	~~1402.1~~	~~19.9~~	~~1226.2~~	~~22.4~~	~~27.1~~	~~1680.9~~	~~52.7~~
FZ4 038	49	51	1.0	0.10341	0.00184	4.19175	0.06881	0.29404	0.00440	1686.2	32.4	1672.4	13.5	1661.7	21.9	1.5	1686.2	32.4
FZ4 041	28	43	0.6	0.10559	0.00195	4.25373	0.07250	0.29222	0.00452	1724.7	33.6	1684.5	14.0	1652.6	22.5	4.2	1724.7	33.6
FZ4 069	278	117	2.4	0.10561	0.00143	4.19081	0.05244	0.28784	0.00379	1724.9	24.6	1672.2	10.3	1630.7	19.0	5.5	1724.9	24.6
FZ4 099	77	90	0.9	0.10613	0.00149	4.50264	0.05872	0.30773	0.00412	1733.9	25.5	1731.5	10.8	1729.5	20.3	0.3	1733.9	25.5
FZ4 078	84	158	0.5	0.10623	0.00118	4.25515	0.04416	0.29054	0.00354	1735.7	20.2	1684.7	8.5	1644.2	17.7	5.3	1735.7	20.2
FZ4 040	128	206	0.6	0.10744	0.00099	4.58955	0.04024	0.30987	0.00358	1756.5	16.8	1747.4	7.3	1740.0	17.6	0.9	1756.5	16.8
FZ4 007	65	88	0.7	0.10827	0.00156	4.23448	0.05622	0.28370	0.00386	1770.4	26.1	1680.7	10.9	1610.0	19.4	9.1	1770.4	26.1
FZ4 092	123	158	0.8	0.10887	0.00119	4.66353	0.04801	0.31070	0.00378	1780.5	19.9	1760.7	8.6	1744.2	18.6	2.0	1780.5	19.9
FZ4 003	315	854	0.4	0.11008	0.00069	4.75861	0.02957	0.31356	0.00335	1800.8	11.4	1777.6	5.2	1758.2	16.4	2.4	1800.8	11.4
FZ4 085	75	119	0.6	0.11103	0.00134	4.97916	0.05620	0.32528	0.00413	1816.3	21.7	1815.8	9.5	1815.4	20.1	0.0	1816.3	21.7
FZ4 023	31	104	0.3	0.11112	0.00129	5.10816	0.05575	0.33345	0.00419	1817.8	21.0	1837.5	9.3	1855.1	20.2	-2.1	1817.8	21.0
FZ4 029	130	266	0.5	0.11134	0.00095	4.97973	0.04044	0.32444	0.00368	1821.3	15.4	1815.9	6.9	1811.4	17.9	0.5	1821.3	15.4
FZ4 016	210	288	0.7	0.11149	0.00089	5.09165	0.03915	0.33128	0.00371	1823.8	14.5	1834.7	6.5	1844.6	18.0	-1.1	1823.8	14.5
FZ4 095	111	86	1.3	0.11151	0.00156	4.99403	0.06503	0.32484	0.00440	1824.1	25.2	1818.3	11.0	1813.3	21.4	0.6	1824.1	25.2
FZ4 057	196	112	1.7	0.11153	0.00134	4.87361	0.05466	0.31698	0.00403	1824.4	21.6	1797.7	9.5	1775.0	19.7	2.7	1824.4	21.6

FZ4 096	66	433	0.2	0.11211	0.00094	4.19391	0.03342	0.27133	0.00305	1833.9	15.1	1672.8	6.5	1547.5	15.5	15.6	1833.9	15.1
FZ4 100	94	82	1.2	0.11295	0.00161	5.01201	0.06658	0.32185	0.00440	1847.4	25.6	1821.4	11.2	1798.8	21.5	2.6	1847.4	25.6
FZ4 054	211	214	1.0	0.11325	0.00104	5.22257	0.04563	0.33451	0.00389	1852.2	16.5	1856.3	7.5	1860.2	18.8	-0.4	1852.2	16.5
FZ4 046	57	142	0.4	0.11374	0.00116	5.07793	0.04891	0.32384	0.00390	1860.1	18.3	1832.4	8.2	1808.5	19.0	2.8	1860.1	18.3
FZ4 012	17	152	0.1	0.11425	0.00112	5.37117	0.04979	0.34101	0.00404	1868.1	17.6	1880.3	7.9	1891.5	19.4	-1.3	1868.1	17.6
FZ4 052	102	199	0.5	0.11533	0.00105	5.62016	0.04904	0.35346	0.00412	1885.1	16.4	1919.2	7.5	1951.1	19.6	-3.5	1885.1	16.4
FZ4 044	43	125	0.3	0.11616	0.00123	5.48506	0.05476	0.34252	0.00419	1898.0	18.9	1898.3	8.6	1898.8	20.1	0.0	1898.0	18.9
FZ4 042	77	79	1.0	0.11759	0.00163	5.25365	0.06770	0.32409	0.00443	1919.9	24.7	1861.4	11.0	1809.7	21.6	5.7	1919.9	24.7
FZ4 019	45	247	0.2	0.11861	0.00097	5.76771	0.04534	0.35274	0.00399	1935.3	14.6	1941.6	6.8	1947.7	19.0	-0.6	1935.3	14.6
FZ4 001	305	690	0.4	0.11883	0.00087	4.95919	0.03503	0.30272	0.00333	1938.7	13.1	1812.4	6.0	1704.8	16.5	12.1	1938.7	13.1
FZ4 066	44	133	0.3	0.11894	0.00128	5.55112	0.05616	0.33854	0.00416	1940.3	19.1	1908.6	8.7	1879.6	20.0	3.1	1940.3	19.1
FZ4 055	60	205	0.3	0.12058	0.00107	5.75330	0.04893	0.34610	0.00402	1964.8	15.8	1939.4	7.4	1916.0	19.2	2.5	1964.8	15.8
FZ4 093	91	74	1.2	0.12091	0.00170	5.80566	0.07601	0.34826	0.00480	1969.8	24.8	1947.3	11.3	1926.3	23.0	2.2	1969.8	24.8
FZ4 047	48	58	0.8	0.12114	0.00172	6.04954	0.08037	0.36225	0.00507	1973.1	25.1	1983.0	11.6	1992.8	24.0	-1.0	1973.1	25.1
FZ4 058	94	212	0.4	0.12134	0.00108	5.73660	0.04884	0.34292	0.00398	1976.1	15.8	1936.9	7.4	1900.7	19.1	3.8	1976.1	15.8
FZ4 070	116	88	1.3	0.12134	0.00219	4.71993	0.07738	0.28214	0.00442	1976.1	31.8	1770.8	13.7	1602.1	22.2	18.9	1976.1	31.8
FZ4 049	84	239	0.4	0.12201	0.00103	5.61955	0.04535	0.33411	0.00382	1985.7	15.0	1919.1	7.0	1858.3	18.5	6.4	1985.7	15.0
FZ4 084	268	342	0.8	0.14673	0.00109	8.19089	0.05925	0.40490	0.00452	2308.1	12.7	2252.3	6.6	2191.6	20.8	5.0	2308.1	12.7
FZ4 022	29	70	0.4	0.15346	0.00247	8.62978	0.12888	0.40792	0.00644	2384.8	27.1	2299.7	13.6	2205.4	29.5	7.5	2384.8	27.1
FZ4 073	82	157	0.5	0.15380	0.00133	9.50517	0.07961	0.44828	0.00529	2388.6	14.7	2388.0	7.7	2387.6	23.5	0.0	2388.6	14.7
FZ4 081	262	395	0.7	0.15423	0.00106	9.28426	0.06282	0.43664	0.00480	2393.3	11.7	2366.5	6.2	2335.6	21.6	2.4	2393.3	11.7
FZ4 080	216	317	0.7	0.15629	0.00126	9.30085	0.07279	0.43165	0.00497	2415.9	13.7	2368.1	7.2	2313.1	22.4	4.3	2415.9	13.7
FZ4 082	183	260	0.7	0.16146	0.00135	9.88133	0.07951	0.44390	0.00517	2471.0	14.0	2423.8	7.4	2368.1	23.1	4.2	2471.0	14.0
FZ4 039	106	191	0.6	0.16268	0.00124	10.32363	0.07682	0.46033	0.00523	2483.7	12.8	2464.2	6.9	2441.0	23.1	1.7	2483.7	12.8
FZ4 043	120	182	0.7	0.16623	0.00126	11.02442	0.08220	0.48109	0.00550	2520.0	12.7	2525.2	6.9	2532.0	24.0	-0.5	2520.0	12.7
FZ4 034	88	93	1.0	0.16669	0.00164	10.83557	0.10211	0.47153	0.00588	2524.7	16.4	2509.1	8.8	2490.3	25.8	1.4	2524.7	16.4
FZ5 053	147	294	0.5	0.05452	0.00167	0.28785	0.00816	0.03830	0.00062	392.5	66.7	256.9	6.4	242.3	3.8	5.7	242.3	3.8
FZ5 064	715	420	1.7	0.05069	0.00124	0.27752	0.00631	0.03972	0.00056	226.5	55.6	248.7	5.0	251.1	3.5	-1.0	251.1	3.5
FZ5 077	105	140	0.7	0.05351	0.00421	0.29573	0.02148	0.04009	0.00136	350.6	168.7	263.1	16.8	253.4	8.4	3.7	253.4	8.4
FZ5 088	45	17	2.6	0.06820	0.00874	0.38178	0.04433	0.04059	0.00227	874.8	244.9	328.3	32.6	256.5	14.1	21.9	256.5	14.1

FZ5 047	356	463	0.8	0.05646	0.00138	0.32063	0.00721	0.04119	0.00060	469.7	53.9	282.4	5.5	260.2	3.7	7.9	260.2	3.7
FZ5 003	260	368	0.7	0.05878	0.00180	0.33540	0.00935	0.04139	0.00070	559.0	65.3	293.7	7.1	261.4	4.3	11.0	261.4	4.3
FZ5 017	335	443	0.8	0.05473	0.00146	0.31312	0.00767	0.04150	0.00063	401.2	57.8	276.6	5.9	262.1	3.9	5.2	262.1	3.9
FZ5 066	57	136	0.4	0.05267	0.00227	0.30531	0.01225	0.04205	0.00083	314.4	95.3	270.5	9.5	265.5	5.1	1.8	265.5	5.1
FZ5 074	50	75	0.7	0.05113	0.00306	0.29994	0.01680	0.04255	0.00105	246.6	132.1	266.4	13.1	268.6	6.5	-0.8	268.6	6.5
FZ5 037	104	225	0.5	0.05532	0.00205	0.32662	0.01107	0.04283	0.00081	425.1	80.0	287.0	8.5	270.3	5.0	5.8	270.3	5.0
FZ5 059	74	137	0.5	0.05257	0.00218	0.31288	0.01204	0.04317	0.00083	310.0	91.7	276.4	9.3	272.5	5.2	1.4	272.5	5.2
FZ5 069	58	67	0.9	0.05363	0.00378	0.32208	0.02115	0.04356	0.00127	355.5	151.5	283.5	16.2	274.9	7.8	3.0	274.9	7.8
FZ5 018	227	341	0.7	0.06957	0.00161	0.41995	0.00877	0.04378	0.00065	915.7	46.8	356.0	6.3	276.2	4.0	22.4	276.2	4.0
FZ5 019	53	61	0.9	0.05225	0.00375	0.31547	0.02146	0.04379	0.00116	296.3	155.6	278.4	16.6	276.3	7.2	0.8	276.3	7.2
FZ5 031	186	279	0.7	0.05689	0.00202	0.34599	0.01126	0.04412	0.00082	486.4	77.3	301.7	8.5	278.3	5.0	7.8	278.3	5.0
FZ5 008	70	178	0.4	0.05148	0.00300	0.31719	0.01719	0.04469	0.00113	262.3	128.7	279.7	13.3	281.8	7.0	-0.8	281.8	7.0
FZ5 061	266	644	0.4	0.05703	0.00113	0.37685	0.00689	0.04793	0.00063	492.3	43.3	324.7	5.1	301.8	3.9	7.1	301.8	3.9
~~FZ5 072~~	~~97~~	~~161~~	~~0.6~~	~~0.07510~~	~~0.00211~~	~~0.52314~~	~~0.01324~~	~~0.05053~~	~~0.00085~~	~~1071.3~~	~~55.5~~	~~427.2~~	~~8.8~~	~~317.8~~	~~5.2~~	~~25.6~~	~~317.8~~	~~5.2~~
FZ5 040	84	111	0.8	0.05813	0.00300	0.47515	0.02254	0.05928	0.00145	534.3	109.7	394.7	15.5	371.3	8.8	5.9	371.3	8.8
FZ5 071	209	405	0.5	0.05848	0.00115	0.49159	0.00894	0.06097	0.00081	547.9	42.5	406.0	6.1	381.5	4.9	6.0	381.5	4.9
~~FZ5 010~~	~~628~~	~~999~~	~~0.6~~	~~0.07913~~	~~0.00097~~	~~0.70149~~	~~0.00781~~	~~0.06430~~	~~0.00076~~	~~1175.3~~	~~24.0~~	~~539.7~~	~~4.7~~	~~401.7~~	~~4.6~~	~~25.6~~	~~401.7~~	~~4.6~~
FZ5 062	97	246	0.4	0.05512	0.00216	0.49548	0.01786	0.06520	0.00128	417.0	84.8	408.6	12.1	407.2	7.8	0.3	407.2	7.8
FZ5 022	29	110	0.3	0.05676	0.00297	0.51072	0.02466	0.06526	0.00159	481.6	112.3	418.9	16.6	407.5	9.6	2.7	407.5	9.6
FZ5 079	6	116	0.0	0.05858	0.00215	0.52970	0.01788	0.06559	0.00125	551.5	78.3	431.6	11.9	409.6	7.6	5.1	409.6	7.6
FZ5 083	114	725	0.2	0.05489	0.00101	0.49761	0.00850	0.06577	0.00084	407.6	40.5	410.1	5.8	410.6	5.1	-0.1	410.6	5.1
FZ5 067	101	178	0.6	0.06031	0.00236	0.55075	0.01972	0.06624	0.00135	614.7	82.5	445.5	12.9	413.5	8.2	7.2	413.5	8.2
FZ5 060	124	189	0.7	0.05595	0.00148	0.51314	0.01258	0.06653	0.00101	449.9	57.8	420.6	8.4	415.2	6.1	1.3	415.2	6.1
FZ5 078	155	346	0.4	0.05608	0.00108	0.51549	0.00918	0.06668	0.00087	455.2	42.0	422.1	6.2	416.1	5.3	1.4	416.1	5.3
FZ5 026	48	105	0.5	0.05309	0.00236	0.48985	0.02029	0.06692	0.00139	332.6	97.7	404.8	13.8	417.6	8.4	-3.2	417.6	8.4
FZ5 011	49	195	0.3	0.05525	0.00166	0.51219	0.01423	0.06724	0.00110	422.3	65.2	419.9	9.6	419.5	6.6	0.1	419.5	6.6
FZ5 023	80	268	0.3	0.05586	0.00161	0.51910	0.01379	0.06740	0.00109	446.5	62.8	424.6	9.2	420.5	6.6	1.0	420.5	6.6
FZ5 045	85	264	0.3	0.05573	0.00138	0.51823	0.01182	0.06745	0.00099	441.3	53.8	424.0	7.9	420.8	6.0	0.8	420.8	6.0
FZ5 085	110	119	0.9	0.05725	0.00185	0.53275	0.01586	0.06750	0.00116	500.8	70.1	433.6	10.5	421.1	7.0	2.9	421.1	7.0
FZ5 070	144	269	0.5	0.05564	0.00122	0.52666	0.01071	0.06866	0.00095	437.6	47.9	429.6	7.1	428.1	5.7	0.3	428.1	5.7
FZ5 052	138	232	0.6	0.05410	0.00130	0.51444	0.01149	0.06897	0.00099	375.0	53.2	421.4	7.7	430.0	5.9	-2.0	430.0	5.9

FZ5 082	103	134	0.8	0.05588	0.00170	0.54361	0.01530	0.07056	0.00115	447.4	66.2	440.8	10.1	439.6	6.9	0.3	439.6	6.9
FZ5 076	145	122	1.2	0.05870	0.00194	0.57263	0.01747	0.07076	0.00122	556.2	70.4	459.7	11.3	440.7	7.4	4.1	440.7	7.4
FZ5 068	235	96	2.5	0.05775	0.00217	0.56377	0.01963	0.07081	0.00133	520.2	80.7	454.0	12.7	441.0	8.0	2.9	441.0	8.0
FZ5 020	232	530	0.4	0.05450	0.00088	0.53388	0.00801	0.07105	0.00087	391.7	35.6	434.4	5.3	442.5	5.3	-1.9	442.5	5.3
FZ5 021	128	198	0.6	0.05802	0.00151	0.57208	0.01371	0.07151	0.00108	530.2	56.3	459.4	8.9	445.3	6.5	3.1	445.3	6.5
FZ5 058	806	173	4.7	0.06101	0.00321	0.60469	0.02911	0.07190	0.00186	639.4	109.5	480.2	18.4	447.6	11.2	6.8	447.6	11.2
FZ5 015	267	23	11.6	0.05555	0.00596	0.55325	0.05608	0.07223	0.00290	434.3	222.8	447.1	36.7	449.6	17.4	-0.6	449.6	17.4
FZ5 033	362	597	0.6	0.05627	0.00083	0.57538	0.00790	0.07417	0.00089	462.1	32.6	461.5	5.1	461.2	5.3	0.1	461.2	5.3
FZ5 027	162	213	0.8	0.05847	0.00155	0.60203	0.01466	0.07469	0.00117	547.3	56.9	478.5	9.3	464.3	7.0	3.0	464.3	7.0
FZ5 005	208	246	0.8	0.05545	0.00136	0.57305	0.01302	0.07495	0.00110	430.3	53.3	460.0	8.4	465.9	6.6	-1.3	465.9	6.6
FZ5 051	177	11	16.1	0.05620	0.00832	0.58967	0.08366	0.07610	0.00368	459.7	299.3	470.7	53.4	472.8	22.0	-0.4	472.8	22.0
FZ5 080	159	146	1.1	0.05936	0.00206	0.62389	0.01990	0.07625	0.00141	580.2	73.8	492.3	12.4	473.7	8.5	3.8	473.7	8.5
FZ5 084	123	188	0.7	0.05715	0.00140	0.60206	0.01362	0.07641	0.00112	496.9	53.7	478.5	8.6	474.7	6.7	0.8	474.7	6.7
FZ5 007	313	357	0.9	0.05608	0.00110	0.59309	0.01074	0.07671	0.00102	455.2	42.7	472.8	6.9	476.4	6.1	-0.8	476.4	6.1
~~FZ5 013~~	~~41~~	~~4~~	~~11.6~~	~~0.12717~~	~~0.02837~~	~~1.38583~~	~~0.28080~~	~~0.07904~~	~~0.00814~~	~~2059.3~~	~~348.6~~	~~882.9~~	~~119.5~~	~~490.4~~	~~48.6~~	~~44.5~~	~~490.4~~	~~48.6~~
FZ5 073	98	100	1.0	0.07712	0.00227	0.90090	0.02405	0.08474	0.00148	1124.2	57.7	652.2	12.8	524.4	8.8	19.6	524.4	8.8
FZ5 004	207	297	0.7	0.06597	0.00109	1.11610	0.01703	0.12271	0.00158	805.3	34.2	761.1	8.2	746.2	9.1	2.0	746.2	9.1
FZ5 035	118	104	1.1	0.07109	0.00208	1.28887	0.03446	0.13151	0.00235	959.9	58.8	840.8	15.3	796.5	13.4	5.3	796.5	13.4
FZ5 016	111	282	0.4	0.07139	0.00098	1.46660	0.01873	0.14900	0.00183	968.6	27.9	916.7	7.7	895.4	10.2	2.3	895.4	10.2
FZ5 056	108	197	0.5	0.09917	0.00115	3.12217	0.03363	0.22836	0.00276	1608.5	21.5	1438.2	8.3	1325.9	14.5	17.6	1608.5	21.5
FZ5 006	77	109	0.7	0.10473	0.00208	3.81203	0.06911	0.26401	0.00420	1709.5	36.0	1595.3	14.6	1510.3	21.4	11.7	1709.5	36.0
FZ5 001	36	38	1.0	0.10501	0.00367	3.82032	0.12133	0.26388	0.00636	1714.5	62.8	1597.0	25.6	1509.6	32.4	12.0	1714.5	62.8
FZ5 050	211	197	1.1	0.10620	0.00111	4.28817	0.04209	0.29286	0.00347	1735.3	19.1	1691.1	8.1	1655.8	17.3	4.6	1735.3	19.1
FZ5 034	41	65	0.6	0.10646	0.00197	3.40912	0.05743	0.23226	0.00352	1739.7	33.5	1506.5	13.2	1346.3	18.4	22.6	1739.7	33.5
FZ5 090	141	115	1.2	0.10844	0.00135	4.78203	0.05320	0.31981	0.00402	1773.2	22.6	1781.7	9.3	1788.8	19.6	-0.9	1773.2	22.6
FZ5 002	210	291	0.7	0.10919	0.00104	4.77621	0.04295	0.31728	0.00369	1785.8	17.2	1780.7	7.6	1776.4	18.0	0.5	1785.8	17.2
FZ5 042	19	33	0.6	0.10933	0.00270	4.59117	0.10445	0.30458	0.00569	1788.3	44.4	1747.7	19.0	1714.0	28.1	4.2	1788.3	44.4
FZ5 039	12	16	0.7	0.11001	0.00388	4.58946	0.14826	0.30260	0.00746	1799.5	62.8	1747.4	26.9	1704.2	36.9	5.3	1799.5	62.8
FZ5 038	45	52	0.9	0.11058	0.00202	4.63786	0.07815	0.30420	0.00469	1809.0	32.8	1756.1	14.1	1712.1	23.2	5.4	1809.0	32.8
FZ5 030	29	79	0.4	0.11082	0.00279	4.67576	0.10807	0.30603	0.00588	1812.9	45.1	1762.9	19.3	1721.1	29.0	5.1	1812.9	45.1
FZ5 063	91	69	1.3	0.11129	0.00173	5.12812	0.07435	0.33425	0.00474	1820.5	28.0	1840.8	12.3	1858.9	22.9	-2.1	1820.5	28.0

FZ5 014	34	90	0.4	0.11222	0.00158	5.00726	0.06552	0.32363	0.00438	1835.7	25.3	1820.6	11.1	1807.4	21.3	1.5	1835.7	25.3
FZ5 055	52	548	0.1	0.11342	0.00091	4.72113	0.03613	0.30193	0.00333	1854.9	14.4	1771.0	6.4	1700.9	16.5	8.3	1854.9	14.4
FZ5 075	46	173	0.3	0.11468	0.00122	5.10577	0.05091	0.32295	0.00387	1874.9	19.0	1837.1	8.5	1804.1	18.9	3.8	1874.9	19.0
FZ5 048	74	73	1.0	0.11474	0.00171	5.09474	0.07048	0.32206	0.00449	1875.8	26.7	1835.2	11.7	1799.8	21.9	4.1	1875.8	26.7
FZ5 043	105	128	0.8	0.11541	0.00137	4.99373	0.05519	0.31384	0.00394	1886.3	21.2	1818.3	9.4	1759.6	19.3	6.7	1886.3	21.2
FZ5 046	22	67	0.3	0.11579	0.00185	5.25710	0.07773	0.32932	0.00476	1892.2	28.4	1861.9	12.6	1835.1	23.1	3.0	1892.2	28.4
~~FZ5 081~~	~~23~~	~~47~~	~~0.5~~	~~0.11784~~	~~0.00272~~	~~3.96773~~	~~0.08264~~	~~0.24425~~	~~0.00431~~	~~1923.7~~	~~40.8~~	~~1627.6~~	~~16.9~~	~~1408.8~~	~~22.4~~	~~26.8~~	~~1923.7~~	~~40.8~~
FZ5 057	22	45	0.5	0.12333	0.00239	4.83863	0.08524	0.28457	0.00463	2005.0	34.0	1791.6	14.8	1614.4	23.2	19.5	2005.0	34.0
FZ5 025	112	99	1.1	0.12431	0.00188	5.55286	0.07744	0.32400	0.00463	2018.9	26.6	1908.8	12.0	1809.2	22.5	10.4	2018.9	26.6
FZ5 029	129	265	0.5	0.12486	0.00130	5.55731	0.05403	0.32283	0.00390	2026.8	18.3	1909.5	8.4	1803.5	19.0	11.0	2026.8	18.3
FZ5 044	167	381	0.4	0.13759	0.00143	6.85058	0.06668	0.36115	0.00441	2197.0	17.9	2092.3	8.6	1987.6	20.9	9.5	2197.0	17.9
FZ5 087	54	33	1.6	0.15660	0.00266	10.05540	0.14639	0.46564	0.00717	2419.3	28.6	2439.9	13.5	2464.4	31.6	-1.9	2419.3	28.6
FZ5 028	91	177	0.5	0.15745	0.00141	9.37986	0.08051	0.43210	0.00511	2428.4	15.1	2375.9	7.9	2315.2	23.0	4.7	2428.4	15.1
FZ5 012	154	192	0.8	0.15768	0.00139	9.63896	0.08163	0.44337	0.00523	2431.0	14.9	2400.9	7.8	2365.7	23.4	2.7	2431.0	14.9
FZ5 086	70	81	0.9	0.15793	0.00183	9.91105	0.10053	0.45509	0.00584	2433.7	19.5	2426.5	9.4	2417.8	25.9	0.7	2433.7	19.5
FZ5 009	96	232	0.4	0.16043	0.00131	9.82043	0.07778	0.44397	0.00512	2460.2	13.8	2418.1	7.3	2368.4	22.9	3.7	2460.2	13.8
FZ5 089	86	184	0.5	0.16089	0.00140	9.93879	0.07843	0.44798	0.00524	2465.0	14.6	2429.1	7.3	2386.3	23.3	3.2	2465.0	14.6
FZ5 041	79	108	0.7	0.16312	0.00205	9.85797	0.11679	0.43834	0.00605	2488.3	21.0	2421.6	10.9	2343.2	27.1	5.8	2488.3	21.0
FZ5 024	84	275	0.3	0.16697	0.00145	9.94530	0.08290	0.43202	0.00508	2527.5	14.5	2429.7	7.7	2314.8	22.9	8.4	2527.5	14.5
FZ5 036	72	425	0.2	0.16790	0.00123	9.62391	0.06828	0.41575	0.00462	2536.8	12.2	2399.5	6.5	2241.1	21.0	11.7	2536.8	12.2
FZ5 065	37	69	0.5	0.16997	0.00200	11.19758	0.12566	0.47786	0.00643	2557.4	19.6	2539.7	10.5	2517.9	28.1	1.5	2557.4	19.6
FZ5 032	10	25	0.4	0.17603	0.00322	11.84872	0.20527	0.48823	0.00876	2615.8	30.2	2592.5	16.2	2563.0	37.9	2.0	2615.8	30.2
FZ5 049	156	278	0.6	0.18509	0.00156	11.78726	0.09565	0.46192	0.00541	2699.0	13.9	2587.7	7.6	2448.0	23.9	9.3	2699.0	13.9
FZ5 054	68	132	0.5	0.18633	0.00166	12.55025	0.10787	0.48856	0.00586	2710.0	14.6	2646.5	8.1	2564.4	25.4	5.4	2710.0	14.6
FZ7 037	87	327	0.3	0.04952	0.00127	0.25439	0.00604	0.03727	0.00054	172.3	58.7	230.1	4.9	235.9	3.4	-2.5	235.9	3.4
FZ7 078	181	1309	0.1	0.05071	0.00067	0.26454	0.00327	0.03784	0.00044	227.8	30.3	238.3	2.6	239.4	2.7	-0.5	239.4	2.7
FZ7 081	304	661	0.5	0.05493	0.00101	0.28764	0.00487	0.03798	0.00049	409.5	40.2	256.7	3.8	240.3	3.0	6.4	240.3	3.0
FZ7 046	80	135	0.6	0.05130	0.00216	0.27247	0.01067	0.03853	0.00074	254.1	93.9	244.7	8.5	243.7	4.6	0.4	243.7	4.6
FZ7 002	132	317	0.4	0.05183	0.00134	0.28774	0.00688	0.04027	0.00060	277.8	58.3	256.8	5.4	254.5	3.7	0.9	254.5	3.7
FZ7 036	62	73	0.8	0.05347	0.00299	0.30316	0.01576	0.04112	0.00099	348.8	121.0	268.9	12.3	259.8	6.1	3.4	259.8	6.1

FZ7 032	92	143	0.6	0.05393	0.00192	0.30744	0.01010	0.04135	0.00074	367.8	78.0	272.2	7.8	261.2	4.6	4.0	261.2	4.6
FZ7 084	226	347	0.7	0.05233	0.00133	0.29892	0.00704	0.04143	0.00061	300.0	57.0	265.6	5.5	261.7	3.8	1.5	261.7	3.8
FZ7 025	40	168	0.2	0.05021	0.00170	0.28933	0.00913	0.04180	0.00071	204.6	76.9	258.0	7.2	264.0	4.4	-2.3	264.0	4.4
FZ7 096	56	76	0.7	0.05461	0.00356	0.31597	0.01899	0.04197	0.00122	396.3	139.5	278.8	14.7	265.0	7.5	4.9	265.0	7.5
FZ7 006	73	133	0.6	0.04927	0.00195	0.28694	0.01061	0.04224	0.00077	160.7	89.9	256.1	8.4	266.7	4.7	-4.1	266.7	4.7
FZ7 061	109	120	0.9	0.06015	0.00239	0.35178	0.01284	0.04242	0.00084	608.9	83.7	306.1	9.6	267.8	5.2	12.5	267.8	5.2
FZ7 065	145	250	0.6	0.05077	0.00152	0.29818	0.00831	0.04260	0.00067	230.4	67.9	265.0	6.5	268.9	4.2	-1.5	268.9	4.2
FZ7 009	106	136	0.8	0.05352	0.00206	0.31891	0.01137	0.04322	0.00082	350.9	84.7	281.1	8.8	272.8	5.0	3.0	272.8	5.0
FZ7 026	116	141	0.8	0.05430	0.00193	0.32714	0.01065	0.04370	0.00080	383.4	77.1	287.4	8.2	275.7	4.9	4.1	275.7	4.9
~~FZ7 013~~	~~160~~	~~208~~	~~0.8~~	~~0.07935~~	~~0.00190~~	~~0.47902~~	~~0.01021~~	~~0.04379~~	~~0.00069~~	~~1180.8~~	~~46.6~~	~~397.4~~	~~7.0~~	~~276.3~~	~~4.2~~	~~30.5~~	~~276.3~~	~~4.2~~
FZ7 094	92	272	0.3	0.05613	0.00162	0.33989	0.00902	0.04392	0.00071	457.3	63.0	297.1	6.8	277.1	4.4	6.7	277.1	4.4
FZ7 042	181	365	0.5	0.05108	0.00113	0.31737	0.00647	0.04506	0.00062	244.6	50.0	279.9	5.0	284.1	3.8	-1.5	284.1	3.8
FZ7 030	142	258	0.5	0.05553	0.00152	0.34542	0.00871	0.04512	0.00070	433.4	59.7	301.3	6.6	284.5	4.3	5.6	284.5	4.3
FZ7 014	276	361	0.8	0.05301	0.00122	0.33200	0.00707	0.04542	0.00064	329.3	51.4	291.1	5.4	286.4	4.0	1.6	286.4	4.0
FZ7 011	133	176	0.8	0.04910	0.00164	0.30912	0.00958	0.04566	0.00076	152.8	76.2	273.5	7.4	287.8	4.7	-5.2	287.8	4.7
FZ7 095	30	60	0.5	0.05256	0.00317	0.33593	0.01895	0.04636	0.00116	310.0	131.5	294.1	14.4	292.1	7.2	0.7	292.1	7.2
FZ7 028	22	33	0.7	0.05231	0.00529	0.34333	0.03310	0.04761	0.00162	299.0	215.2	299.7	25.0	299.8	10.0	0.0	299.8	10.0
FZ7 097	111	104	1.1	0.05186	0.00215	0.39638	0.01539	0.05544	0.00105	279.2	92.2	339.0	11.2	347.8	6.4	-2.6	347.8	6.4
FZ7 072	60	135	0.4	0.05555	0.00179	0.49492	0.01472	0.06463	0.00110	434.1	69.9	408.3	10.0	403.7	6.7	1.1	403.7	6.7
FZ7 091	2	70	0.0	0.05324	0.00238	0.48528	0.02026	0.06612	0.00135	338.8	97.9	401.7	13.9	412.7	8.2	-2.7	412.7	8.2
FZ7 053	95	111	0.9	0.05695	0.00205	0.52758	0.01750	0.06720	0.00125	488.9	78.2	430.2	11.6	419.3	7.6	2.5	419.3	7.6
FZ7 062	108	208	0.5	0.05576	0.00150	0.51777	0.01281	0.06736	0.00105	442.4	58.4	423.7	8.6	420.2	6.3	0.8	420.2	6.3
FZ7 031	39	46	0.8	0.05700	0.00367	0.52966	0.03153	0.06740	0.00195	491.1	136.6	431.6	20.9	420.4	11.8	2.6	420.4	11.8
FZ7 068	70	106	0.7	0.05451	0.00180	0.50960	0.01556	0.06782	0.00117	392.0	71.6	418.2	10.5	423.0	7.1	-1.1	423.0	7.1
FZ7 063	80	106	0.8	0.07603	0.00232	0.72439	0.01996	0.06911	0.00124	1095.8	60.0	553.3	11.8	430.8	7.5	22.1	430.8	7.5
FZ7 015	140	218	0.6	0.05305	0.00129	0.50618	0.01137	0.06921	0.00101	331.0	54.1	415.9	7.7	431.4	6.1	-3.7	431.4	6.1
FZ7 066	208	344	0.6	0.05499	0.00098	0.54319	0.00896	0.07164	0.00092	412.0	39.0	440.5	5.9	446.1	5.5	-1.3	446.1	5.5
FZ7 052	15	39	0.4	0.05610	0.00320	0.55637	0.02949	0.07194	0.00185	455.8	122.3	449.2	19.2	447.8	11.1	0.3	447.8	11.1
FZ7 003	26	71	0.4	0.05442	0.00214	0.54484	0.01977	0.07263	0.00144	388.2	85.3	441.6	13.0	452.0	8.7	-2.4	452.0	8.7
FZ7 047	62	122	0.5	0.05584	0.00164	0.56859	0.01548	0.07386	0.00120	445.7	64.0	457.1	10.0	459.4	7.2	-0.5	459.4	7.2
FZ7 100	168	141	1.2	0.05554	0.00154	0.57460	0.01465	0.07504	0.00119	433.8	60.2	461.0	9.5	466.5	7.1	-1.2	466.5	7.1

FZ7 074	200	315	0.6	0.05557	0.00106	0.57650	0.01020	0.07525	0.00100	435.0	41.6	462.2	6.6	467.7	6.0	-1.2	467.7	6.0
FZ7 049	77	96	0.8	0.05796	0.00181	0.60479	0.01739	0.07569	0.00130	527.8	67.0	480.3	11.0	470.3	7.8	2.1	470.3	7.8
FZ7 005	37	63	0.6	0.05827	0.00247	0.61119	0.02409	0.07608	0.00156	539.2	90.9	484.3	15.2	472.7	9.3	2.4	472.7	9.3
FZ7 060	52	89	0.6	0.05475	0.00197	0.57707	0.01932	0.07645	0.00138	401.9	78.0	462.6	12.4	474.9	8.3	-2.7	474.9	8.3
FZ7 004	215	364	0.6	0.05505	0.00091	0.61058	0.00939	0.08044	0.00101	414.4	36.3	483.9	5.9	498.8	6.0	-3.1	498.8	6.0
FZ7 039	100	184	0.5	0.05516	0.00116	0.61293	0.01192	0.08059	0.00111	418.7	45.6	485.4	7.5	499.7	6.6	-2.9	499.7	6.6
FZ7 089	55	361	0.2	0.05706	0.00089	0.65502	0.00953	0.08326	0.00103	493.4	34.4	511.6	5.8	515.6	6.1	-0.8	515.6	6.1
FZ7 033	21	48	0.4	0.08041	0.00301	0.92557	0.03124	0.08350	0.00177	1207.0	71.9	665.3	16.5	517.0	10.5	22.3	517.0	10.5
FZ7 058	86	208	0.4	0.06350	0.00107	1.08711	0.01697	0.12418	0.00161	724.9	35.4	747.1	8.3	754.6	9.2	-1.0	754.6	9.2
FZ7 045	38	38	1.0	0.08895	0.00329	1.52435	0.05061	0.12430	0.00274	1403.1	69.1	940.2	20.4	755.3	15.7	19.7	755.3	15.7
FZ7 073	22	38	0.6	0.09574	0.00332	3.35669	0.10623	0.25430	0.00588	1542.8	63.7	1494.4	24.8	1460.6	30.2	5.3	1542.8	63.7
~~FZ7 027~~	~~80~~	~~168~~	~~0.5~~	~~0.09814~~	~~0.00188~~	~~2.37707~~	~~0.04093~~	~~0.17569~~	~~0.00266~~	~~1589.0~~	~~35.4~~	~~1235.7~~	~~12.3~~	~~1043.4~~	~~14.6~~	~~34.3~~	~~1589.0~~	~~35.4~~
FZ7 098	27	32	0.8	0.09856	0.00254	4.06997	0.09846	0.29952	0.00526	1597.1	47.4	1648.3	19.7	1689.0	26.1	-5.8	1597.1	47.4
FZ7 092	137	121	1.1	0.10096	0.00124	3.95443	0.04505	0.28410	0.00358	1641.9	22.5	1624.9	9.2	1612.0	18.0	1.8	1641.9	22.5
FZ7 038	53	94	0.6	0.10166	0.00149	3.53884	0.04780	0.25250	0.00342	1654.6	27.0	1535.9	10.7	1451.4	17.6	12.3	1654.6	27.0
FZ7 035	28	69	0.4	0.10386	0.00151	4.42193	0.05999	0.30882	0.00423	1694.2	26.7	1716.5	11.2	1734.9	20.8	-2.4	1694.2	26.7
FZ7 077	41	42	1.0	0.10458	0.00205	4.28761	0.07730	0.29740	0.00477	1706.9	35.6	1691.0	14.8	1678.4	23.7	1.7	1706.9	35.6
FZ7 022	66	119	0.6	0.10483	0.00125	4.50104	0.05017	0.31144	0.00393	1711.3	21.7	1731.2	9.3	1747.8	19.3	-2.1	1711.3	21.7
FZ7 040	29	48	0.6	0.10532	0.00183	4.48663	0.07203	0.30899	0.00467	1719.9	31.6	1728.5	13.3	1735.7	23.0	-0.9	1719.9	31.6
FZ7 099	126	15	8.4	0.10613	0.00351	4.54885	0.13914	0.31090	0.00713	1734.0	59.4	1740.0	25.5	1745.1	35.1	-0.6	1734.0	59.4
FZ7 085	30	59	0.5	0.10648	0.00177	4.43077	0.06791	0.30182	0.00441	1740.0	30.1	1718.1	12.7	1700.3	21.8	2.3	1740.0	30.1
FZ7 057	42	69	0.6	0.10678	0.00163	4.52778	0.06397	0.30757	0.00431	1745.2	27.6	1736.1	11.8	1728.7	21.3	0.9	1745.2	27.6
FZ7 001	101	84	1.2	0.10875	0.00145	4.68619	0.05811	0.31256	0.00415	1778.5	24.2	1764.8	10.4	1753.3	20.4	1.4	1778.5	24.2
FZ7 093	74	102	0.7	0.11056	0.00137	4.88438	0.05668	0.32047	0.00413	1808.6	22.4	1799.6	9.8	1792.0	20.1	0.9	1808.6	22.4
FZ7 064	55	68	0.8	0.11162	0.00165	5.05765	0.06962	0.32867	0.00460	1825.9	26.6	1829.0	11.7	1831.9	22.3	-0.3	1825.9	26.6
FZ7 083	29	22	1.3	0.11172	0.00283	4.96025	0.11571	0.32205	0.00625	1827.6	45.2	1812.6	19.7	1799.7	30.5	1.5	1827.6	45.2
FZ7 023	49	142	0.3	0.11217	0.00119	4.98958	0.04983	0.32265	0.00394	1834.9	19.1	1817.6	8.5	1802.6	19.2	1.8	1834.9	19.1
FZ7 079	52	173	0.3	0.11265	0.00158	4.59532	0.05942	0.29589	0.00402	1842.6	25.2	1748.4	10.8	1670.9	20.0	9.3	1842.6	25.2
FZ7 008	170	64	2.6	0.11279	0.00168	5.44380	0.07577	0.35007	0.00496	1844.9	26.7	1891.8	11.9	1934.9	23.7	-4.9	1844.9	26.7
FZ7 076	74	323	0.2	0.11328	0.00099	4.25352	0.03499	0.27236	0.00311	1852.7	15.7	1684.4	6.8	1552.8	15.7	16.2	1852.7	15.7
FZ7 021	103	287	0.4	0.11444	0.00100	5.09153	0.04241	0.32271	0.00372	1871.1	15.7	1834.7	7.1	1803.0	18.1	3.6	1871.1	15.7

FZ7 080	69	104	0.7	0.11450	0.00134	5.34874	0.05872	0.33884	0.00429	1872.0	21.0	1876.7	9.4	1881.1	20.7	-0.5	1872.0	21.0
FZ7 051	84	105	0.8	0.11819	0.00155	5.29864	0.06459	0.32518	0.00434	1929.1	23.3	1868.6	10.4	1815.0	21.1	5.9	1929.1	23.3
FZ7 018	64	143	0.4	0.11852	0.00131	4.92751	0.05052	0.30157	0.00374	1934.0	19.6	1807.0	8.7	1699.1	18.5	12.1	1934.0	19.6
FZ7 059	50	83	0.6	0.12011	0.00167	5.43766	0.06995	0.32839	0.00452	1957.8	24.6	1890.8	11.0	1830.6	21.9	6.5	1957.8	24.6
FZ7 017	76	175	0.4	0.12020	0.00111	5.87395	0.05145	0.35445	0.00417	1959.2	16.3	1957.4	7.6	1955.8	19.9	0.2	1959.2	16.3
FZ7 090	180	132	1.4	0.12037	0.00127	5.59759	0.05551	0.33730	0.00413	1961.8	18.7	1915.7	8.5	1873.7	19.9	4.5	1961.8	18.7
FZ7 086	74	93	0.8	0.12188	0.00155	5.75268	0.06818	0.34237	0.00453	1983.9	22.5	1939.3	10.3	1898.1	21.8	4.3	1983.9	22.5
FZ7 087	3	22	0.1	0.12299	0.00291	5.92295	0.12898	0.34933	0.00666	2000.0	41.4	1964.6	18.9	1931.4	31.8	3.4	2000.0	41.4
FZ7 019	70	109	0.6	0.12355	0.00152	5.74222	0.06589	0.33711	0.00442	2008.2	21.7	1937.7	9.9	1872.7	21.3	6.7	2008.2	21.7
FZ7 024	5	14	0.3	0.12425	0.00352	6.12316	0.16044	0.35745	0.00783	2018.1	49.5	1993.6	22.9	1970.1	37.2	2.4	2018.1	49.5
FZ7 043	190	314	0.6	0.13341	0.00099	6.67689	0.04814	0.36303	0.00406	2143.3	13.0	2069.6	6.4	1996.5	19.2	6.8	2143.3	13.0
FZ7 029	55	66	0.8	0.13838	0.00173	7.44932	0.08715	0.39046	0.00526	2207.1	21.5	2166.9	10.5	2125.0	24.4	3.7	2207.1	21.5
FZ7 016	220	295	0.7	0.13915	0.00108	7.60758	0.05739	0.39657	0.00451	2216.6	13.5	2185.8	6.8	2153.2	20.8	2.9	2216.6	13.5
FZ7 012	72	72	1.0	0.14378	0.00246	7.50989	0.11856	0.37887	0.00611	2273.2	29.2	2174.2	14.2	2071.0	28.6	8.9	2273.2	29.2
FZ7 010	278	350	0.8	0.14690	0.00102	8.58431	0.05835	0.42386	0.00470	2310.1	11.8	2294.9	6.2	2278.0	21.3	1.4	2310.1	11.8
FZ7 034	51	83	0.6	0.14734	0.00165	8.15185	0.08594	0.40131	0.00520	2315.3	19.1	2248.0	9.5	2175.1	23.9	6.1	2315.3	19.1
FZ7 075	47	38	1.2	0.15017	0.00312	7.89172	0.15060	0.38118	0.00705	2347.9	35.0	2218.7	17.2	2081.8	32.9	11.3	2347.9	35.0
FZ7 069	157	246	0.6	0.15048	0.00117	8.48152	0.06390	0.40884	0.00466	2351.3	13.3	2283.9	6.8	2209.6	21.3	6.0	2351.3	13.3
FZ7 082	55	96	0.6	0.15245	0.00167	8.99400	0.09315	0.42792	0.00552	2373.6	18.5	2337.4	9.5	2296.4	24.9	3.3	2373.6	18.5
FZ7 048	90	213	0.4	0.15412	0.00142	9.71635	0.08598	0.45729	0.00554	2392.1	15.6	2408.3	8.2	2427.6	24.5	-1.5	2392.1	15.6
FZ7 044	116	225	0.5	0.15691	0.00118	9.61857	0.07055	0.44463	0.00505	2422.6	12.7	2398.9	6.8	2371.3	22.5	2.1	2422.6	12.7
FZ7 088	136	244	0.6	0.15693	0.00116	9.50963	0.06828	0.43956	0.00494	2422.8	12.4	2388.5	6.6	2348.7	22.1	3.1	2422.8	12.4
FZ7 056	35	49	0.7	0.15790	0.00570	9.53380	0.31952	0.43796	0.01331	2433.3	60.0	2390.8	30.8	2341.5	59.7	3.8	2433.3	60.0
FZ7 070	85	132	0.6	0.15881	0.00147	9.77393	0.08654	0.44643	0.00541	2443.0	15.5	2413.7	8.2	2379.4	24.1	2.6	2443.0	15.5
FZ7 055	85	57	1.5	0.16017	0.00194	10.20047	0.11720	0.46194	0.00633	2457.5	20.3	2453.1	10.6	2448.1	27.9	0.4	2457.5	20.3
FZ7 054	106	243	0.4	0.16043	0.00119	9.97300	0.07239	0.45092	0.00510	2460.2	12.5	2432.3	6.7	2399.3	22.7	2.5	2460.2	12.5
FZ7 020	49	122	0.4	0.16118	0.00152	8.75126	0.07816	0.39381	0.00480	2468.1	15.9	2312.4	8.1	2140.5	22.2	13.3	2468.1	15.9
FZ7 067	42	76	0.6	0.16253	0.00181	10.13311	0.10727	0.45222	0.00595	2482.2	18.7	2447.0	9.8	2405.1	26.4	3.1	2482.2	18.7
FZ7 071	113	49	2.3	0.16572	0.00209	11.04809	0.13277	0.48356	0.00682	2514.9	21.0	2527.2	11.2	2542.8	29.7	-1.1	2514.9	21.0
FZ7 041	1	6	0.2	0.17207	0.00695	11.08715	0.41836	0.46737	0.01590	2577.9	65.9	2530.5	35.1	2472.0	69.9	4.1	2577.9	65.9
FZ7 007	48	160	0.3	0.20667	0.00191	16.11013	0.14571	0.56540	0.00714	2879.6	14.9	2883.4	8.7	2888.9	29.4	-0.3	2879.6	14.9

FZ7 050	64	282	0.2	0.22615	0.00155	18.12497	0.12389	0.58132	0.00657	3025.0	10.9	2996.4	6.6	2954.1	26.8	2.3	3025.0	10.9
